# Nematode eggshells: A new anatomical and terminological framework, with a critical review of relevant literature and suggested guidelines for the interpretation and reporting of eggshell imagery

**DOI:** 10.1051/parasite/2023007

**Published:** 2023-03-15

**Authors:** Alan Thomas Bond, David George Huffman

**Affiliations:** 1 Warnell School of Forestry and Natural Resources, University of Georgia 180 E. Green St. Athens GA 30602 USA; 2 Department of Biology (Wildlife Ecology and Aquatic Resources), Freeman Aquatic Biology Bldg., Texas State University San Marcos TX 78666 USA

**Keywords:** Trichinelloidea, nematode eggshell, ultrastructure, vitelline layer, lipid-rich layer, CPG layer

## Abstract

A literature review for a recent ultrastructural study of a trichinelloid eggshell revealed consistently occurring errors in the literature on nematode eggshell anatomy. Examples included nematodes of medical, veterinary, and agricultural importance in several orders. Previous researchers had warned of some of these errors decades ago, but a comprehensive solution was not offered until 2012 when a clarifying new anatomical and developmental interpretation of nematode eggshells was proposed by members of the *Caenorhabditis elegans* Research Community. However, their findings were explained using arcane acronyms and technical jargon intended for an audience of experimental molecular geneticists, and so their papers have rarely been cited outside the *C. elegans* community. Herein we (1) provide a critical review of nematode eggshell literature in which we correct errors and relabel imagery in important historical reports; (2) describe common reporting errors and their causes using language familiar to researchers having a basic understanding of microscopy and nematode eggs; (3) recommend a new hexalaminar anatomical and terminological framework for nematode eggshells based on the 2012 *C. elegans* framework; and (4) recommend new unambiguous terms appropriate for the embryonated/larvated eggs regularly encountered by practicing nematodologists to replace ambiguous or ontogenetically restricted terms in the 2012 *C. elegans* framework. We also (5) propose a resolution to conflicting claims made by the *C. elegans* team versus classical literature regarding Layer #3, (6) extend the *C. elegans* hexalaminar framework to include the polar plugs of trichinelloids, and (7) report new findings regarding trichinelloid eggshell structure.

## Abstract (fr)

Une revue de la littérature pour une étude ultrastructurale récente de la coque de l’œuf d’un trichinelloïde a révélé des erreurs récurrentes dans la littérature sur l’anatomie de la coque de l’œuf des nématodes. Les exemples comprenaient des nématodes d’importance médicale, vétérinaire et agricole dans plusieurs ordres. Des chercheurs avaient mis en garde contre certaines de ces erreurs il y a des décennies, mais une solution complète n’a été proposée qu’en 2012, lorsqu’une nouvelle interprétation anatomique et développementale clarifiant la structure des coques des œufs de nématodes a été proposée par des membres de la communauté de recherche de *Caenorhabditis elegans*. Cependant, leurs découvertes ont été expliquées à l’aide d’acronymes mystérieux et d’un jargon technique destiné à un public de généticiens moléculaires expérimentaux, et leurs articles ont donc rarement été cités en dehors de la communauté de *C. elegans*. Ici, nous (1) fournissons une revue critique de la littérature sur les coques des œufs de nématodes dans laquelle nous corrigeons les erreurs et réétiquetons les images dans des rapports historiques importants; (2) décrivons les erreurs de description courantes et leurs causes en utilisant un langage familier aux chercheurs ayant une compréhension de base de la microscopie et des œufs de nématodes; (3) recommandons un nouveau cadre anatomique et terminologique hexalaminaire pour les coques des œufs de nématodes basé sur le cadre de *C. elegans* de 2012; et (4) recommandons de nouveaux termes non ambigus appropriés pour les œufs embryonnés/larvés régulièrement rencontrés par les spécialistes de nématodes en exercice pour remplacer les termes ambigus ou à restriction ontogénétique dans le cadre de *C. elegans* de 2012. Nous proposons également (5) une résolution des affirmations contradictoires de l’équipe *C. elegans* par rapport à la littérature classique concernant la couche 3, (6) étendons le cadre hexalaminaire de *C. elegans* pour inclure les bouchons polaires des trichinelloïdes, et (7) signalons de nouvelles découvertes concernant la structure de la coque des œufs des trichinelloïdes.

*The present was an egg laid by the past*

*that had the future inside its shell*

Zora Neale Hurston

## 1 Introduction

The study of the nematode eggshell, which had advanced rather slowly since the mid-20th Century, was abruptly moved forward in 2012 by a single publication that presented remarkable new findings on the hierarchical assembly of the *Caenorhabditis elegans* [68] eggshell. The Olson *et al.* (2012) [83] paper revealed new information regarding the composition and developmental sequence of eggshell layers and offered a comprehensive new hexalaminar anatomical and terminological framework for the nematode eggshell to replace the classical trilaminar framework. A review of the Olson *et al.* (2012) [83] paper and a second draft of the framework was later included in a chapter of WormBook Online, “The *C. elegans* eggshell” by Stein and Golden (2018) [103]. While Stein and Golden (2018) [103] implied that their findings had refuted previous eggshell findings for other taxa, no specific applications to the eggshell anatomy of other taxa were attempted in either paper, and the terminological schemes of both papers pertained to premitotic events of early development and contained several terms with a long history of ambiguity and misapplication.

In the 10 years that have passed since the Olson *et al.* (2012) [83] paper appeared, this exciting and revolutionary paper has only been cited slightly more than 100 times – almost all of them by other scientists in the *C. elegans* Research Community. We recently searched Google Scholar for papers since 2012 containing “nematode AND eggshell AND (lipid-layer OR lipid-rich-layer);” a search string likely to return most research on nematode eggshell development, composition and anatomy. The search returned 108 hits; remarkably, only 1 of these had cited either Olson *et al.* (2012) [83] or Stein and Golden (2018) [103], and several had carried forward errors that the new *C. elegans* framework had corrected years earlier. Despite the parsimonious new framework and clarifying revelations provided by the 2012 Olson report, papers restating errors that had been corrected, sometimes decades earlier (for instance, referring to a membrane internal to the Chitinous Layer as the vitelline membrane [108]) are continuing to appear in respected journals.

We were drawn into this adventure during an ultrastructural study of the eggshell of *Huffmanela huffmani* [74], an obscure histozoic nematode of fish that is endemic to three springs in central Texas and a fish farm near Tampa, Florida, USA [23]. Our transmission electron microscopy (TEM) images had revealed structures in the *H. huffmani* eggshell that we were unable to identify using the classical “trilaminar” anatomical/terminological eggshell framework that had been used for decades to describe the nematode eggshell. The only references we could find to help us definitively identify the mysterious structures and spaces we were seeing were those by Olson *et al.* (2012) [83] and Stein and Golden (2018) [103] in which their new hexalaminar framework accommodated most of the undefined structures we had been seeing in our imagery.

Herein we offer a modified and expanded hexalaminar framework adapted from the *C. elegans* framework to describe our findings and then extend those findings to reinterpret published eggshell and polar-plug imagery from other trichinelloids, and ultimately, from other orders of the phylum at large. We also declare that the terminology used in the classical trilaminar framework, variations of which have been heretofore used as interpretive models in almost all nematode eggshell studies, is oversimplified, ambiguous, and inadequate to accommodate layers and sublayers clearly observable in most published TEMs of nematode eggshells from orders for which TEMs are publicly available. Thus, we recommend that the classical trilaminar framework, in all its variations, should be abandoned and replaced with some variation of the Olson *et al.* (2012) [83] hexalaminar framework, with order-specific adaptations where warranted.

The concerns we are alluding to are not trivial ivory-tower issues. Phytoparasitic nematodes number around 1400 species [71] and have been estimated to cause global crop losses as high as USD $1.7 × 10^11^ annually [41]. Zooparasitic nematodes of mammals alone exceed 8900 species and affect about 3.5 × 10^9^ humans globally [2]. The eggshells of some important nematode parasites are among the most resistant protective shields known in biology; and knowing how to defeat these resistant shields, or to interfere with the complex processes that lead to eggshell development in nematodes, can lead to practical and effective solutions to some of these problems. As Bird and Bird (1991) [16] stated, “… information on the structure and chemistry of the eggshell is important both from the fundamental and applied points of view.” However, before such research findings can be effectively communicated, an anatomically accurate and unambiguous terminological model must be available for researchers to use in their reports.

The classical trilaminar framework, variants of which have been almost universally accepted by nematodologists for well over 75 years, fails the tests for anatomical accuracy and unambiguous communication, and should be replaced with a more parsimonious framework that can accommodate recent findings, account for all consistently occurring eggshell structures, and retrospectively correct the misleading image interpretations that have hindered advancement in the field for decades. Herein we offer a revised and expanded third draft of the revolutionary Olson *et al.* (2012) [83] hexalaminar framework as a proposed new interpretive and reporting framework for nematode eggshell research.

## 2 Terminology, history, and goals

### 2.1 Terminology, general

This review contains many cross references to internal and external imagery. In order to reduce likelihood of confusion, all cross references to internal imagery or tables will use the labels “Figure” or “Table” and all cross references to external imagery or tables will use the labels “fig.” or “pl.” or “tab.”

All terms recommended for the new terminological scheme will be spelled-out each time with initial caps. New Latin terms never used for nematode eggs will be italicized. Some figures will be labeled with abbreviations recommended for terms of the new hexalaminar framework. A glossary is included for the reader’s convenience (Section 6).

Nouns and adjectives referencing developmental stages of “eggs” have been simplified herein as follows: all developmental stages of an ovum prior to union with a sperm cell are collectively referred to as “oocyte/meiotic”; stages following sperm penetration and cortical remodeling up to the first mitotic cleavage as “zygote/zygotic”; first cleavage to vermiform appearance as “embryo/mitotic”; and worm-like appearance and behavior as “larva/larval.”

All persons who have, at some time and to some degree, engaged in the scientific study of nematodes from any lifestyle or domain of the biosphere, including species that are free-living in marine, freshwater, and terrestrial environments, as well as species that engage in some form of symbiotic relationship with any other animal or plant species, are herein collectively refer to as “nematodologists,” while begging forgiveness in advance from specialists who might have preferred a more respectable term for their vocational appellation.

### 2.2 Terminology associated with the classical trilaminar framework

**Figure 1:**
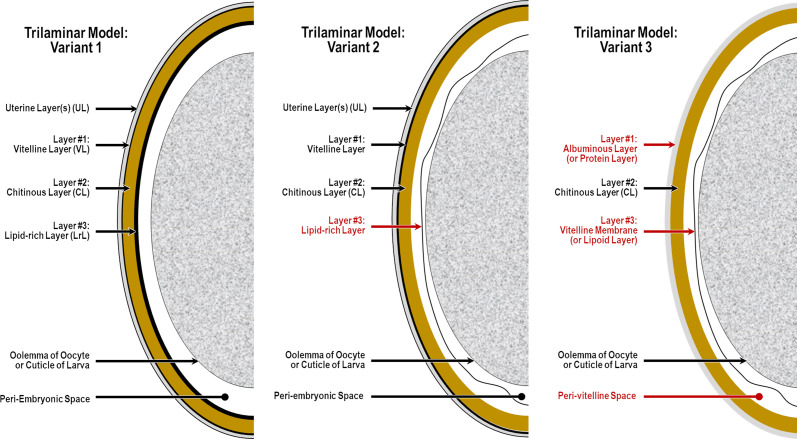
Schematics of three of the most common variants of the classical trilaminar framework for nematode eggshells, listed in order of estimated frequency of use at this time. Red labels represent noteworthy changes from the variant to the left. *Notes*: (1) there are two fundamentally different structures labeled as “the Lipid-rich Layer” among variants of the trilaminar framework, both of which have been shown experimentally to be lipid-rich in tested species; (2) there are two fundamentally different structures labeled as “the vitelline layer;” (3) Variant #3 skips the primal first eggshell layer to form and considers the exogenous Uterine Layer to be #1; (4) all layers (except for the Uterine Layer) in any variant of the trilaminar model are apparently present in all mitotic-stage eggshells, but advocates of one variant do not recognize some layers labeled in other variants; and (5) the only consistencies among all these trilaminar variants are that they all claim three layers and all that we have seen place the Chitinous Layer at position #2.

Beginning in the 19th Century [80] and continuing into recent writings [45], most authors studying nematode eggshells have assumed that there are three distinct endogenous layers (formed by the embryo) as defined in what has been classically referred to as the “trilaminar model,” the most frequently used variants of which we represent schematically in Figure 1. This trilaminar assumption has since been almost universally applied, in various permutations, as the default anatomical and terminological framework for communicating research findings from nematode eggshell studies.

#### 2.2.1 Layer #1: Vitelline Layer

This is the name usually given to the outer-most endogenous layer in the most popular Variant 1 of the classical trilaminar eggshell scheme. Although perhaps not entirely endogenous (the scaffolding for the layer may be an exogenous product of ovarian cells), it is so extensively remodeled by the oocyte at the moment of sperm penetration [53, 54] that it subsequently qualifies as “endogenous.” It is a thin layer, and very difficult to discern without TEM. It is the outside surface of an undisturbed egg viewed with scanning electron microscopy (SEM), unless it is covered by debris or maternal secretions. In most TEM imagery of fully developed nematode eggshells we have studied, this membrane persists through hatching as a uniformly electron dense and uniformly thin membrane that appears to have been electro-plated onto the usually smooth surface of a pre-existing chitinous layer. Quite the contrary, this membrane appeared over the oolemma almost immediately after it was penetrated by the first sperm [103] to prevent entry by other sperm (“fast block” to polyspermy) and is now known to be the very first structure of the incipient eggshell. Indeed, in most variants of the classical trilaminar and the new hexalaminar anatomical models, this membrane correctly becomes the prime reference structure by means of which all other components of the eggshell are logically related.

#### 2.2.2 Layer #2: the Chitinous Layer

This is usually the thickest layer of the eggshell. Deposition of the Chitinous Layer onto the internal surface of the vitelline layer begins almost immediately after fertilization and is the backup (“slow block”) polyspermy barrier. The Chitinous Layer is semipermeable but usually rigid and serves as an enclosure to protect the developing larva from physical trauma [103].

#### 2.2.3 Layer #3: the “Lipid-rich” Layer

This is the innermost layer of the trilaminar eggshell in the most commonly seen variant, especially in recent research from a trilaminar perspective. As the name implies, this layer has been thought to contain lipids in all nematodes. It is still considered by many authors (outside the *C. elegans* Research Community) to be equivalent to the hypothetical “permeability barrier” that protects the nematode embryo/larva from osmotic and desiccation threats and provides eggs of some species with near legendary resistance to desiccation, toxins and anthelmintics [102]. Some authors, having been constrained to three layers by the trilaminar model, have inadvertently labeled as the Lipid-rich Layer, structures we now know to be two or even three ontogenetically different eggshell layers, sometimes in the same paper and in eggs of the same species.

### 2.3 Problems with the classical trilaminar framework

Although most nematodologists refer to three layers in the classical trilaminar framework, there continues at the time of this writing to be considerable disagreement on the nature, function, and labeling of the three layers chosen by an author to represent the trilaminar anatomical model. Chitwood (1938) [28], a proponent of Variant 2 in Figure 1, opened a discussion of nematode eggshell structure with, “It is now generally recognized that the so-called shell consists of 3 layers of different chemical composition, these being known as (1) the albuminous layer, (2) the shell proper …, and (3) the fibrous layer (vitelline membrane).” Wharton (1980) [117], a proponent of Variant 1, opened a section on eggshell structure with, “The nematode egg-shell may consist of anything from 1 to 5 layers. The most frequently observed pattern consists of an inner lipid layer, a middle chitinous layer and an outer vitelline layer.” Unfortunately, the additional two layers Wharton referred to are exogenous layers, and do not pertain to any of the layers we were seeing internal to the Lipid-rich Layer.

As the resolution of microscopical and histochemical analyses has improved, it has become progressively more difficult to force all discernible layers of the nematode eggshell into this simple framework, and limited attempts to start a conversation about identifying and correcting problematic aspects of the model have been unsuccessful. For instance, in 1971, Yuen (1971) [124] provided a useful review of previous failed attempts to standardize early terminology and made some reasonable suggestions for reform that were also largely ignored by nematodologists. Some subsequent TEM workers warned of various problems with the optical properties of the Chitinous Layer that were introducing artifacts into the eggshell anatomy literature, etc. Very little changed until 2012 when the Olson *et al.* (2012) [83] study shed definitive new light onto the ontogenetic program and compositional layering of the nematode eggshell, resolved many of the inherent problems in the classical trilaminar framework, and offered a comprehensive new hexalaminar anatomical model with three additional layers that would accommodate most of the structures unexplainable by variants of the trilaminar model. Amazingly, it, too, has seen nearly zero citations outside the *C. elegans* Research Community as editors of life science journals continue to accept for publication manuscripts referring to nematode eggshells using obsolete concepts, hopelessly ambiguous terminology, and anatomical conclusions proven wrong, sometimes decades earlier; thus, adding additional error to a body of literature already bloated with ambiguity. Full disclosure – one of us (DGH) is among the perpetrators of such errant narratives, and has contributed prose to articles that added to said ambiguity.

As we searched the available literature on nematode eggshells, trying to make sense of what we were seeing in our own TEM imagery, three problematic issues began to emerge:

- variants of the trilaminar framework are still almost universally used as the standard framework for describing nematode eggshell architecture, but no single one of the variants can account for all of the layers that are consistently seen external to the embryo in published TEMs from several nematode orders;
- the hexalaminar anatomical model offered by Olson *et al.* (2012) [83] has the potential to become a much more parsimonious and comprehensive fit to every published TEM of nematode eggshells we have studied than is any variant of the classical trilaminar anatomical model; and
- difficulties with the classical trilaminar framework began to emerge as early as the mid-20th Century, and the time for starting a serious conversation about a replacement anatomical/terminological framework for nematode eggshells is long overdue.

Other more detailed interpretive and reporting problems associated with the trilaminar framework will be addressed as we discuss our draft adaptation and extension of the hexalaminar eggshell framework based on the Olson *et al.* (2012) [83] findings.

### 2.4 Focus and proposed terminological standards

Our immediate focus is to apply the new hexalaminar framework to our TEMs of the eggshell of *H. huffmani*, and then show how the framework fits quite well to other trichinelloids and surprisingly well to available eggshell TEMs of most other nematode groups we have reviewed, including *C. elegans*. We were initially skeptical that an anatomical model based on the eggs of one somewhat bizarre species (*C. elegans*) from an evolutionarily advanced nematode class, and which spends only a few hours in the egg, might be useful for describing the eggs of a group of worms from a primitive nematode superfamily (Trichinelloidea) which might spend several years inside the egg. However, as we began to examine published TEM imagery, the centripetal depositional sequence and general appearance of layers described by Stein and Golden (2018) [103] appeared to be surprisingly consistent across the TEM eggshell imagery we have studied from most economically and medically important nematode groups and appeared to be consistent with almost everything we observed in published TEMs of trichinelloids.

The new hexalaminar framework for nematode eggshells includes a total of six endogenous layers of unique origin and composition surrounding the embryo. The research focus of the Olson *et al.* (2012) [83] eggshell study appears to have been ontogenetic sequence, genetic control and process, and resulting composition. Thus, every compositionally distinct layer in the depositional sequence of the hexalaminar framework was numbered and then named in the context of oogenesis, fertilization, and early eggshell development. Stein and Golden (2018) [103] later attempted to add a seventh exogenous but unnumbered layer to the model and modified the terminology somewhat, but still retained an ontogenetic flavor to the terminological scheme and also continued to use some terms that are historically ambiguous and, in some cases, anatomically and functionally misleading.

**Figure 2:**
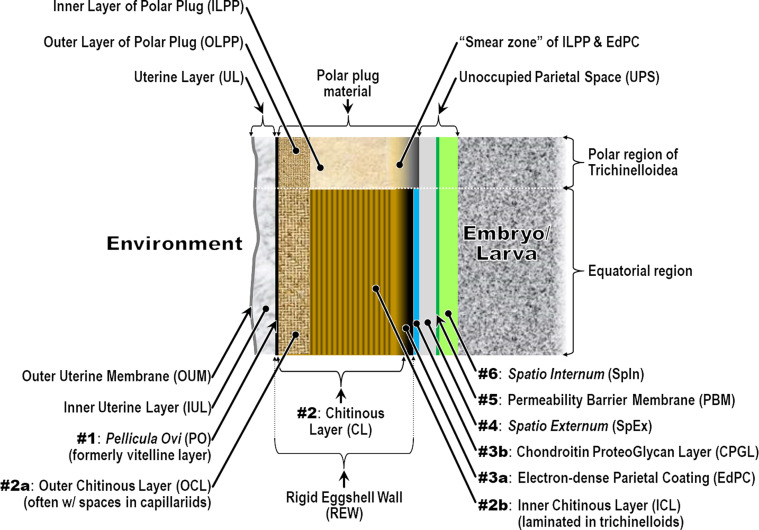
Schematic of proposed new anatomical/terminological framework representing a generalized nematode eggshell and trichinelloid polar plug. Framework was adapted from the Olson *et al.* (2012) [83] hexalaminar framework for *C. elegans* as modified by Stein and Golden (2018) [103], except that the Lipid-rich Layer (Layer #3a) was retained from the classical trilaminar model.

We have adopted the general pattern of the Olson *et al.* (2012) [83] findings for our draft framework (Figure 2) but replaced early ontogenetic or historically conflated terms with unambiguous terms that are more descriptive of how each of the layers appears in the later embryonated/larvated eggs encountered by most practicing nematodologists. In some cases, our self-imposed guidelines to avoid historically conflated terminology required us to resort to fresh Latin terms that are more descriptive and have never been applied to nematode eggs. Our proposed new terminological scheme for nematode eggshell architecture is presented in Section 4 and then explained and justified, layer by layer, in depositional sequence. We follow the ontogenetic numbering model of Stein and Golden (2018) [103], but divided Layers #2 and #3 into two sublayers each.

In our proposed new terminological scheme, we will use the term “eggshell” *sensu lato* to refer collectively to all layers of the embryonated/larvated nematode egg, whether the layer is shell-like, fluid-like, or membranous, or originates from endogenous or exogenous sources. When referring to the rigid wall of the eggshell that determines its shape (sometimes referred to by nematodologists as the “true shell” or “wall”) we will use the term Rigid Eggshell Wall (REW). Our anatomical model addresses all layers external to the outermost plasma membranes of the embryo (or cuticle of the larva) that are (1) known to be of differing chemical composition relative to radially adjacent materials or (2) consistently appearing to be bounded by materials of differing textural properties or electron density in TEMs.

### 2.5 Goals

Our goals for this work are as follows.

1. To propose and justify a comprehensive new and anatomically appropriate hexalaminar framework for communicating research findings regarding eggshell anatomy and function that corresponds well to eggshell TEMs of frequently investigated orders that we studied in the literature, and especially to Trichinelloidea.
2. To enable future investigators working with the thick-shelled eggs of trichinelloids to distinguish actual anatomical features of studied eggshells from artifacts one might see in light microscopy (LM) and SEM studies, and which are still routinely included in published drawings and descriptions of trichinelloid eggs.
3. To reveal several structures we have discovered in our TEM studies that are not previously documented for *Huffmanela* eggshells nor, to our knowledge, the eggs of any other trichinelloids, and to interpret these structures from the perspective of the new hexalaminar framework.
4. To provide retrospective re-interpretations of some illustrative examples of previously published LM, SEM, and TEM imagery of the eggshells of *Huffmanela* and selected species from other orders, based on our draft hexalaminar framework. These re-labeled tracings of published images could serve as guides for how to properly label new imagery from future research, and also provide a more parsimonious and consistent reinterpretation of what we now know is improperly labeled imagery in classical and current eggshell literature.

Note that our goals are not taxonomic or histochemical. We do not anticipate that this report will challenge the validity of any nominal species or higher taxa, although some species may ultimately be reassigned as a result. Neither do we present new experimental findings regarding the chemical composition or ontogenetic processes involved in the formation or functions of the various eggshell layers. Our broader focus, beyond reporting our own TEM findings, is primarily on (1) the re-interpretation of published microscope imagery in consideration of the comprehensive new model of Olson *et al.* (2012) [83] (as modified by our new findings), (2) providing a terminological scheme optimized for more precise and accurate reporting of eggshell anatomy, and (3) reducing the number of artifacts included in future drawings and labeling of eggshell imagery.

## 3 Methods

### 3.1 Imagery

All photos, except where indicated otherwise, are from our laboratory. Interpretive tracings were made from PDF copies of journal articles obtained via library subscription or Inter-Library Loan services of the Texas State University Library System, or from publicly accessible online sources. Color choices in tracings and drawings follow colors consistently utilized by Stein and Golden (2018) [103] in their refinement of the Olson (2012) hexalaminar framework, except that brown is substituted for their red to represent the Chitinous Layer.

### 3.2 Permits and source of specimens

Eggs of *Huffmanela huffmani* were collected from the swimbladders of sunfishes (*Lepomis* spp.) captured from the Upper San Marcos River in Hays County, Texas (29.887870, −97.934541), Anson Spring at the headwaters of the South Concho River in Tom Green County, Texas (31.135874, −100.493819), and Clear Creek Springs in Menard County, Texas (30.905299, −99.958649). Researchers complied with approved collection, transportation, holding, and euthanasia methods as specified in Texas State University IACUC protocol #73, and wild fish were collected under Texas Parks & Wildlife Diversity Permit #SPR-0913-124.

### 3.3 Transmission electron microscopy

Small specimens of egg-laden tissue were cut from the swim bladders and fixed in 2% glutaraldehyde for 2 h. Each specimen was washed twice for 15 min in a 0.1 M cacodylate solution and then placed in osmium tetroxide to post-fix for 2 h. Another series of cacodylate washes was performed, after which each specimen was dehydrated through a graded series of ethanol. Once a specimen was in absolute ethanol, it was placed into a TEM embedding capsule. LR White Hard resin in absolute ethanol (1:1) was added to the capsule so as to cover the specimen and the capsule was incubated for approximately 12 h at room temperature. The resin/ethanol mixture was then pipetted out, and straight LR White Hard resin was added, and the capsule was incubated for another 3 h at room temperature. A final exchange of resin was added to the capsule before it was heated at 65 °C for at least 24 h to harden the resin. Once polymerized, the resin block was shaped and placed into an ultramicrotome (Leica Reichert Ultracut S). Sections were cut with a glass knife to approximately 70 nm thickness and collected on copper grids. The sections were then viewed using a JOEL JEM 1200EXII.

**Figure 3:**
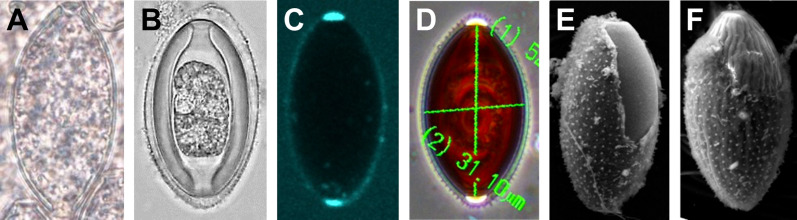
Eggs of *Huffmanela huffmani* at approximately the same scale under various conditions. (A) LM of a recently fertilized (as indicated by the presence of polar plugs) egg of *Huffmanela huffmani* freshly deposited into host tissue with the Uterine Layer poorly developed and overlying a thin Chitinous Layer; (B) LM of a slightly older egg with embryo having now bent into the “comma stage,” and which has developed (since being deposited in host tissue) a much thicker but still hyaline Chitinous Layer and the characteristic Surface Papillae on the Uterine Layer; (C) confocal of an unstained fresh egg with the Outer Layer of the Polar Plug autofluorescing brightly (no dye); (D) phase contrast of an older, larvated egg showing now-darkened Chitinous Layer, regular spacing of Surface Papillae, and transparent, highly refractile nature of the Outer Layer of Polar Plug; (E) SEM of normal egg with Uterine Layer partially torn away showing normally smooth outer surface of the Rigid Eggshell Wall; (F) Egg with aberrant wrinkles in Chitinous Layer that affect Uterine Layer. (A)–(D): live eggs in spring water or saline.

We had many false starts and lost egg parts due to failures during sectioning. The Chitinous Layer of the fully developed *H. huffmani* eggshell is often very thick (Figure 3B) and extremely hard, and this can cause unexpected losses of parts from the sections. The most common problems were caused when the knife encountered the Chitinous Layer broadside and moved the wall down-cut, tearing it loose from the up-cut part of the section and compressing the down-cut part into folds. Glass knives quickly dull and wear out before a block has been sectioned causing streaks and the crumbling or smearing of the chitin. Even experienced ultramicrotomists should test and refine technique using eggs of a readily available thick-shelled species such as *H. huffmani* before attempting to section *Huffmanela* eggs from a limited museum collection.

### 3.4 Scanning electron microscopy

Swim bladders containing eggs were shredded using a pair of probes in a dish of saline. Patches of tissue that lacked eggs were removed, and loose eggs were pipetted out of the dish and fixed and post-fixed using the same method as for the TEM samples. Once the eggs had completed an ethanol dehydration series, they were placed in porous pots and dried in a critical point dryer using acetone. Eggs were then removed from the pots and placed on carbon tape; at which time they were sputter coated with carbon. Once coated, the eggs were viewed under a JEOL JSM-6010.

## 4 Results and discussion

In this section we present, describe, and discuss, from the outermost to innermost endogenous layers, the application of our new draft hexalaminar framework of the generalized nematode eggshell (Figure 2). For each layer, we provide examples of how our framework is superior to the classical trilaminar framework, provide a critical review of the relevant literature pertaining to each, and comment on problems associated with common technique protocols. For most layers, we recommend new terms to replace the ambiguous terms from the trilaminar framework and the ontogenetic terms from the Stein and Golden (2018) [103] modification of the Olson *et al.* (2012) [83] framework. We then recommend best practices less likely to result in artifacts continuing to be incorporated into future reports of eggshell anatomy (with emphasis on those caused by optical phenomena and procedural practices).

As we describe the problems with the trilaminar framework, we will start with the equatorial region of the eggshell to establish a context for a subsequent review of problems we have discovered related uniquely to the polar regions of trichinelloid eggs. Figure 3 is a collection of *H. huffmani* egg images from our laboratory that will serve as reference examples for some discussions. Tracings from published egg imagery of other species will be introduced to provide an anatomical context for complex prose we must sometimes use and to correct labeling in historically important images.

After all the layers have been addressed, we will adapt the framework to the polar regions of trichinelloids, and then summarize some serious challenges to certain aspects of the Olson Team’s interpretation of their experiments related to Layer #3 of the *C. elegans* eggshell, and the impact their conclusions might have on nematodologists’ perceptions regarding eggshells of the phylum at large.

### 4.1 Layer #1: The *Pellicula Ovi*; formerly “outer vitelline layer”

Prior even to ovulation, the plasma lemma of a *C. elegans* oocyte becomes covered by a thin external membrane [53] that is apparently attractive to sperm [69], contains chitin-binding proteins [53], and will become Layer #1 (the *Pellicula Ovi*) of the eggshell after fertilization. Johnston and Dennis (2012) [53] refer to this extracellular membrane before and after fertilization as the vitelline membrane. Almost immediately after penetration of the oocyte plasma lemma by the first sperm, a series of very rapid modifications, sometimes referred to as “egg activation,” affect the entire surface of the cell [69]. The entire external membrane is remodeled and quickly takes on a crenulated appearance and becomes resistant to penetration by the many other sperm that began probing the oocyte after it entered the spermatheca. At this point, what was formerly a layer receptive to sperm has been extensively remodeled by cortical granule exocytosis from the oocyte into a sperm-resistant Layer #1 of the incipient eggshell. We cannot confidently say whether the original coating originates from the oocyte or from the maternal tissues surrounding early oocyte or both, but the extensive remodeling of the layer by the oocyte after fertilization is sufficient for the oocyte to claim ownership of the layer and thus make it Layer #1 of the endogenously produced eggshell.

From this point forward, we recommend referring to this layer in nematodes as the *Pellicula Ovi* (formerly vitelline layer or membrane). Thus, the first function for the *Pellicula Ovi* (and only function we know of) is to serve as what has been called the “fast block” to polyspermy [53]. Coincident with this, chitin synthase in the quickly reorganized plasma membrane of the oocyte begins secreting precursors of the Chitinous Layer (Layer #2) in between the plasma membrane and the *Pellicula Ovi*. This chitin apparently binds with the chitin-binding domains in what is now the *Pellicula Ovi* and becomes Layer #2, the Chitinous Layer. This coating of chitin that is quickly laid down across the entire parietal surface of the *Pellicula Ovi* apparently [53] provides a secondary block to any sperm that managed to get through the fast block while it was being remodeled into the *Pellicula Ovi*. Thus, the outermost layer of Layer #2 (Chitinous Layer) initially functions as backup for the fast-block function of the *Pellicula Ovi* and is sometimes referred to as the “slow block” to polyspermy [53].

#### 4.1.1 The origin of Layer #1

The outermost layer of the Rigid Eggshell Wall and first layer of the eggshell to appear is the *Pellicula Ovi* (formerly the outer vitelline layer). Olson *et al.* (2012) [83] and many others have used the term “vitelline layer” to refer to Layer #1.

Melesse and Bembenek (2019) [69] provide a detailed step by step account of the molecular events following fertilization that result in the formation of Layer #1 in *C. elegans*. However, not all investigators agree on how this membrane is formed [18, 88, 124], and multiple attempts to define a unified origin narrative and compositional and structural profile for Layer #1 across the phylum have yielded even more varied results. Thus, while there seems to be widespread agreement regarding the primary functions of Layer #1 related to fertilization, until more nematode species are systematically investigated regarding the origin, structure, and composition of Layer #1, ideally by a single team, it would probably be best to conclude that the details regarding the nature of Layer #1 are not as conserved across the phylum as some may have assumed. On the other hand, every nematode egg TEM we have seen that shows the outer surface of the Rigid Eggshell Wall also shows a uniformly thin and uniformly electron dense layer lining the outside surface, and that layer apparently serves as a required scaffold on which the rest of the eggshell is constructed [83]; thus, its designation as Layer #1.

#### 4.1.2 Nomenclatural chaos

Unfortunately, the terms “vitelline layer,” “vitelline membrane,” and “vitelline coat,” which are often used to refer to Layer #1 (including in the *C. elegans* framework), have been used interchangeably in nematodology literature to refer to at least four distinctly different structures of differing compositions, origins, and unrelated functions in the nematode eggshell; thus, the term “vitelline” used as a modifier for any structure in the nematode egg turns out to be probably the most ambiguous anatomical term in nematode literature. A new name is needed for this structure to clarify the functional and anatomical ambiguities in current and historical literature, and to provide for the unambiguous storage and retrieval of future findings regarding Layer #1. It should be noted that we are not the first to recommend complete abandonment of the term “vitelline membrane,” for it has caused decades of confusion in developmental studies in nematodes and other groups as well [4, 36]. As we shall see, there are other issues with nematode eggshell terminology, but the term “vitelline” in the nematode literature is by far the most problematic and should be resolved first.

#### 4.1.3 Examples of conflation of “vitelline” in nematode literature

*1st Confusing Use:* The first (and perhaps most appropriate) application of the term vitelline membrane is in reference to the outermost membrane of the pre-fertilization oocyte. This membrane has the ephemeral role of attracting sperm (of the species) and then allowing entry of (usually) only one [54]. Almost immediately after entry of the first sperm, that same layer that attracted the sperm is remodeled to become the first line of defense against entry by any of the remaining sperm (which could otherwise result in lethal polyspermy). This second function for Layer #1 only lasts for a few minutes in nematodes and then the function of polyspermy barrier is assumed by Layer #2, the Chitinous Layer, which quickly forms on the inner surface of Layer #1 [54]. These transient roles for Layer #1 are not trivial by any means, but they are fleeting. While the remnant of Layer #1 may last for years on the outer surface of a viable nematode eggshell, it has no known function (except perhaps in trichinelloids) after its inner surface has become coated with chitin. Some authors have acknowledged the change of roles of Layer #1 after fertilization by changing its name at that point from “vitelline membrane” to “vitelline layer” [18]. However, both modifiers, “layer” and “membrane,” have also been used interchangeably by various authors to refer to Layer #1, sometimes in successive sentences in the same report [97]; so, switching from membrane to layer would only compound the terminological confusion.

*2nd Confusing Use:* Unfortunately, investigators continue to use the term “vitelline layer” in a second, now-proven-wrong sense [103], to refer to a membrane *interior* to the Chitinous Layer [108]. The confusion apparently derives from an earlier theory that the polyspermy barrier is formed *inside* the oolemma [60], and that this new membrane subsequently becomes a malleable membrane between Layer #2 and the embryo. This “inner vitelline layer,” sometimes also called the “embryonic” or “larval” membrane, has nothing whatsoever to do with protecting the oocyte from polyspermy and has now been demonstrated by Olson *et al.* (2012) [83] to be, instead, Layer #5 in depositional sequence, the Permeability Barrier Membrane. To further exacerbate this matter, some investigators who are using the term “vitelline membrane” in reference to a membrane interior to the chitin do not even employ the modifiers “inner” or “underlying,” thus rendering their findings difficult to properly interpret. Indeed, unless authors of a report have used one of the modifiers “inner” or “outer” when referring to the “vitelline layer” of a nematode egg, the reader should check the callouts included on the imagery to determine for certain whether they are using the term to refer to Layer #1 or Layer #5 of the hexalaminar anatomical model. For another example, suppose a researcher is searching for information regarding the composition of Layer #1 and sees a paper with this actual title, “The lipid components in the vitelline membrane of *Ascaris lumbricoides* eggs” [38]. Unless this paper is read very carefully, one might conclude that the authors had provided convincing evidence that ascaryl alcohols are present in Layer #1, when in fact, they are referring to Layer #5!

*3rd Confusing Use:* The ambiguity gets more complex with the third historical application of “vitelline layer.” In the first published TEM study of a *Huffmanela* eggshell, Žďárská *et al.* (2001) [125] used “vitelline membrane” to refer to the outermost layer of the *H. huffmani* egg, which we now know is a Uterine Layer (Section 4.2), and which is deposited by the uterus onto the *outer* surface of Layer #1! This error, unfortunately, was repeated in subsequent reports for other species of the genus [64, 77, 91, 121]. The egg specimens studied by Žďárská *et al.* (2001) [125] had extremely electron-dense Chitinous Layers (nearly black in their TEMs) and the authors overlooked a uniformly thin, uniformly electron dense, membrane-like layer closely appressed to the external surface of the Chitinous Layer in their fig. 4. Since most variants of the trilaminar anatomical model specify that the vitelline layer is immediately external to the chitin, Žďárská *et al.* (2001) [125] then referred to what they saw as the outermost layer of the *H. huffmani* egg as the vitelline layer. While the SEM imagery of Žďárská *et al.* (2001) [125] has been helpful in revealing the ultrastructure of this outer envelope, the layer clearly does not even remotely resemble Layer #1 of any other nematode egg for which Layer #1 has been imaged or described. It does, however, resemble the exogenous Uterine Layer that coats the outside surface of Layer #1 of nematode eggshells in other orders where it has been imaged (pl. 4B of Wharton (1979) [112], pl. 1C of Wharton (1979) [113], pl. 5 of Wharton (1980) [117]).

**Figure 4:**
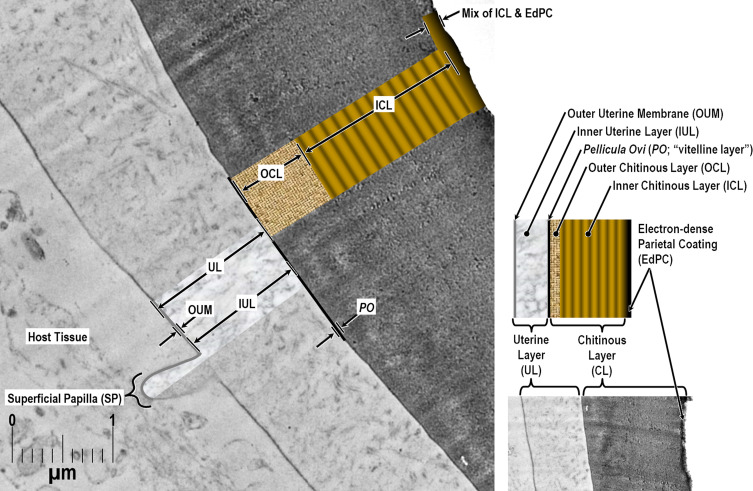
Hi-res TEM imagery of the Uterine Layer, *Pellicula Ovi*, and Chitinous Layer of the *Huffmanela huffmani* eggshell (fine lamellated features of Inner Chitinous Layer smeared by dulled knife).

When we first noticed this paradox, we were reluctant to equate the “vitelline layer” of Žďárská *et al.* (2001) [125] to the “Uterine Layer” of other nematode groups for two reasons: (1) we knew of no other species in the Trichinelloidea with eggs described as having a Uterine Layer, and (2) in all other species for which we have seen a Uterine Layer imaged, the Uterine Layer is subtended by a much thinner electron-dense Layer #1 between the Uterine Layer and the outermost boundary of the much thicker Chitinous Layer [113, 115, 117]. Such a layer appeared to be altogether missing from the Žďárská imagery and also from our recent TEM imagery of *H. huffmani* eggs. Perplexed for several weeks by this paradox, we more closely re-examined our TEM imagery of *H. huffman*i eggs and, after some adjustments to luminance curves, a distinct and homogeneous layer (Figure 4) of the same general appearance as that described for Layer #1 of other species emerged and became consistently obvious (see other examples in figs. 2 and 4 of Appleton and White (1989) [6], and figs. 2, 3, 6, and 7 of Wharton and Jenkins (1978) [120]). We then re-examined the TEM images from Žďárská *et al.* (2001) [125], and after adjusting the luminance curves in an image copied from an uncompressed pdf of their fig. 4, the previously unnoticed layer became clearly visible in their imagery as well. If one closely examines their fig. 4 between labels OC and V, and again, between labels V and C of their fig. 8, the same can be seen without adjustment, once the observer knows what to look for. Using the 200 nm scale bar on their fig. 4 and assuming a section cut in an approximately transverse plane, the presumptive Layer #1 was estimated to be about 10–12 nm thick. In our own *H. huffmani* imagery, Layer #1 was estimated to be about 10 nm thick. Thus, the outer envelope described for many *Huffmanela* species is a Uterine Layer, not a vitelline membrane, and the true Layer #1 was hiding right where it is supposed to be – between the base of the Uterine Layer and the outer surface of the Chitinous Layer.

*4th Confusing Use:* The term “vitelline coat” is sometimes used to refer to special materials temporarily deposited onto the surface of Layer #1 by the ovary itself and might be responsible for sperm attraction, among other things. However, the use of the modifier “vitelline” to refer to this layer makes it possible for the casual reader to conflate it with one of the other 3 conflated uses above.

Any attempt to rescue the term “vitelline” for use in reference to any structure in the nematode eggshell would require untangling the associated confusion and contorted application history and also require much explanation with references going back nearly two centuries; however, this would not stop the 4-way potential for confusion from being carried forward by continued use of the term. This problem will only be resolved when use of the term “vitelline [whatever]” in reference to any part of an eggshell of any nematode species is discouraged. The term should henceforth only be used as we have used it here; to refer to former terms that were historically misapplied to structures of current interest, and the structures of current interest must now have new and unique names unencumbered by past misapplication.

#### 4.1.4 A recommended solution

Our main criteria for a replacement term to refer to Layer #1 were that (1) it must be meaningful in its application to an embryonated/larvated egg rather than just to the prezygotic phase, and (2) it must have never been used in the literature to refer to any other part of a nematode eggshell. Regarding criterion 1, Dumont and Brummet (1980) [36], while working with the development of *Fundulus* eggs, recognized that a different name was needed for the layer that encloses the embryo until hatching. They chose “chorion” for this purpose. However, that term has been used for eggs of widely divergent groups, with the presumption that all such layers must, somehow, be homologous, and it has also been used to refer to the Chitinous Layer of nematode eggs. Nonetheless, Layer #1 seems to be universally present in nematode eggshells, is usually persistent, and should have an unambiguous label that is more descriptive of its location during its long tenure rather than to its embryonic origin or an ephemeral function.

Unfortunately, choosing a replacement term was complicated by the fact that previous workers have been elegantly creative in their choice of English words used as alternatives or modifiers for this first layer of the nematode eggshell, and there appears to be no reasonably descriptive English choices left untainted by misuse. Thus, the term we are offering that meets the two criteria for replacement of “vitelline” in our proposed terminological scheme is *Pellicula Ovi* (pronounced “pay-LICK-yoo-lah OH-vee”), which is Latin for, roughly, “*membrane of the egg*.” A Google Scholar search for “pellicula-ovi AND nematode” returned 0 hits. The term has occasionally been used to refer to the membranes of the chicken egg immediately internal to the limy shell, but at least there will be no ambiguity associated with the use of this term in a nematode eggshell context. We understand that there will be considerable resistance to this recommendation (Section 4.1.2), especially a new Latin term, but, regardless, a new term meeting the above criteria is long overdue. We also understand that zoologists who have spent their careers working on early developmental stages of the nematode egg may be particularly resistant and reject the suggestion that “vitelline” be abandoned. Unfortunately, this will result in condemning their future findings to comingle with the confusion of the past misuses of the term. This could be avoided by replacing “vitelline” with a unique term (we recommend *Pellicula Ovi*) and simply indicating, the first time it is used, as “… formerly ‘vitelline Layer.’”

### 4.2 The Uterine Layer

The Uterine Layer is unnumbered, exogenous in origin, and deposited on the outer surface of the *Pellicula Ovi* by the mother worm after fertilization. It often persists as a prominent and sometimes ornate feature documented in TEMs of embryonated/larvated eggs of some species in several orders [117]. Some authors have noted in their TEMs of genera not known to possess a Uterine Layer, that there is an extremely thin, fuzzy, irregular coating of “particulate matter” of unknown composition or origin on the *Pellicula Ovi* and sometimes on the outer surface of the Uterine Layer as well [114]. Such “coatings” are also most likely of an exogenous origin, perhaps incidental, and could be of ovarian, uterine, vaginal, or microbial-biofilm origin; however, not much is known about these, and we will not be discussing such coatings further.

Since *C. elegans* does not have an obvious Uterine Layer, the layer was not included in the Olson *et al.* (2012) [83] framework. However, Stein and Golden (2018) [103], in their review of the Olson framework opened their passage on the topic by referring (on p. 17) to a theory of “a post-ovulatory envelope … or a fertilization membrane;” a “thin crenellated covering on top of the vitelline layer” that is either “disassembled following the synthesis of the eggshell” or “remains to stably protect the unfertilized oocyte.” They then referred to this structure with, “This so-called uterine layer has been observed for numerous nematode species.” This statement is unfortunate, since the layer Stein and Golden (2018) [103] are referring to in this passage is not the true Uterine Layer that “has been observed for numerous nematode species.” Indeed, what they are referring to in *C. elegans* is often referred to as “fine particulate matter” that is sometimes seen in high resolution TEMs to be coating the *Pellicula Ovi* or even the Uterine Layer itself. See fig. 5 of Wharton (1980) [117] for some examples of what most nematodologists are referring to with their use of the term “Uterine Layer.”

Wharton (1979) [115] pointed out that the mucoid exogenous layers of ascarids and heterakids are not architecturally or compositionally comparable to those of oxyurids in structure or composition, and implied that they should not be referred to as Uterine Layers. However, we do not expect there to be a uniform consensus of origin, composition, structure, and function to emerge from subsequent study of these exogenous layers, and anticipate that they are much more evolutionarily adaptive and less conserved than are the endogenous parts of the eggshell. Thus, we think there is no problem in retaining the term Uterine Layer to refer to any exogenous layers added to the external surface of the *Pellicula Ovi* by the mother worm after fertilization, including those of ascarids, heterakids, and now, the “envelopes” of *Huffmanela* spp. which were earlier misidentified as the “vitelline layer” [125] and herein revealed to be exogenous.

One might argue that we are not justified in claiming that *Huffmanela* is the first known trichinelloid genus with a Uterine Layer since we have done no chemical tests on the outer envelope or studied its origin and development. However, all of the compositionally distinct layers of the nematode eggshell between the *Pellicula Ovi* and the embryo/larva are formed endogenously in sequence, starting with the Chitinous Layer deposited on the inside surface of the *Pellicula Ovi* and progressing centripetally [83]. In contrast, any layer external to the *Pellicula Ovi* must necessarily have been deposited there exogenously *after* fertilization, with the reproductive tract of the mother worm as the probable origin.

TEMs of some Uterine Layers show a thin outer electron-dense membrane similar in appearance and thickness to the *Pellicula Ovi* (Figure 4 and Wharton (1979) [114]) which overlays a much thicker electron-lucid matrix (Figure 4 and Wharton (1980) [117]). This same pattern is repeated in the “outer envelope” of the *H. huffmani* egg which Žďárská *et al.* (2001) [125] had labeled as the “vitelline layer.” In our new framework, we will refer to the thin, membranous, electron-dense outer membrane of the Uterine Layer as the Outer Uterine Membrane, and the underlying and much thicker (in developed eggs) electron-lucid matrix as the Inner Uterine Layer. Please note that while the Outer Uterine Membrane/Inner Uterine Layer model seems to be quite common among nematodes with a Uterine Layer, ascarids have only one homogenous layer to the Uterine Layer, while some others, like *Syphacia obvelata*, have three or four layers [114].

**Figure 5:**
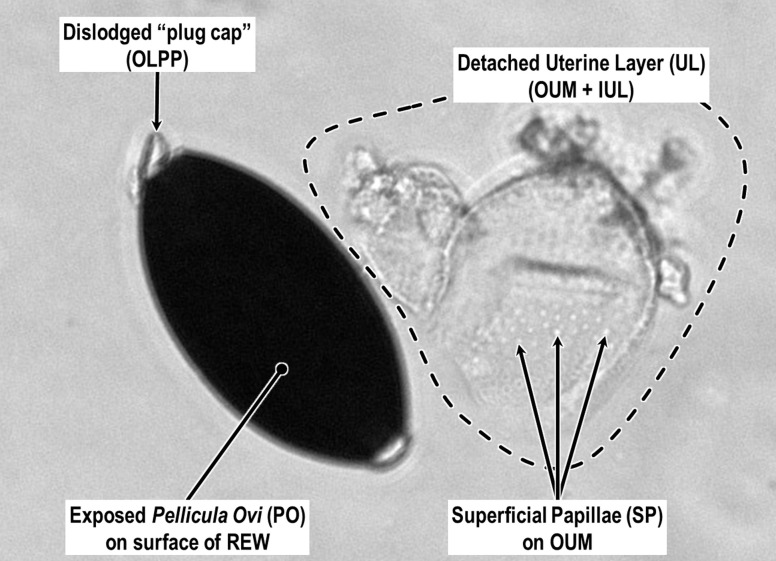
LM photomicrograph of *H. huffmani* egg showing one polar cap (Outer Layer of Polar Plug) dislodged (arrow) and the entire Uterine Layer detached and laying to the side.

Sometimes the Inner Uterine Layer of *Huffmanela* species becomes separated from the *Pellicula Ovi* and the intervening space fills with ambient fluid (our Figure 3B, figs. 1A–E, M, N, 2C, G of Justine (2004) [55]; fig. 1C of Moravec *et al.* (1998) [78]; and fig. 12 of Ruiz *et al.* (2013) [96]) or becomes completely detached as in our Figure 5. We recommend referring to the fluid-filled space as the Sub Uterine-Layer Space, with the understanding that it is not a deposited “layer,” but merely a common detachment artifact resulting from traumatic disturbance. This detachment phenomenon seems to be quite common in *H. huffmani* and several other species of the genus, but is not commonly imaged or mentioned in other groups reported as having Uterine Layers.

**Figure 6:**
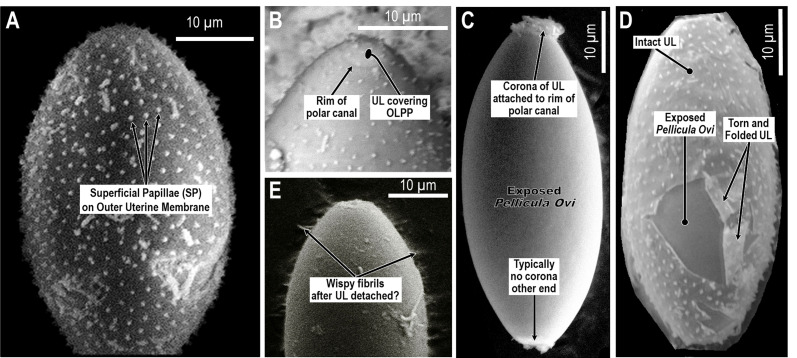
SEMs of *Huffmanela huffmani* eggs: (A) consistent nearest-neighbor spacing of Superficial Papillae; (B) Superficial Papillae covering the Outer Layer of Polar Plug of an egg that apparently had hatched prior to SEM prep showing near normal continuation of egg’s ellipsoidal contour with minimal depression of the Outer Layer of Polar Plug into canal; (C) one of several eggs seen lacking any Uterine Layer except for a corona of Uterine Layer attached to the canal rim of, typically, only one of the polar canals; (D) torn Uterine Layer folded back upon itself exposing smooth *Pellicula Ovi* beneath; (E) wispy filaments, fibrils, and granular material of unknown function attached to *Pellicula Ovi* following apparently recent removal of Uterine Layer.

Geometrically complex and ornate architectures sometimes seen on nematode eggs are often manifestations of the Uterine Layer [114, 117]. At least one species of *Huffmanela* (*H. japonica* [78]: figs. 2C, D; 3B–E) appears to have the Inner Uterine Layer sculpted into columns and spaces similar to those described for *Aspiculuris tetraptera* [81] in fig. 1 of Wharton (1979) [115]. However, *H. japonica* does not appear to have pores in the Outer Uterine Membrane connected to the spaces as in some other orders. The Superficial Papillae (sometimes referred to as “spines” or “superficial projections”) on the eggs of some species of *Huffmanela*, including *H. huffmani* (Figures 3 and 6), are also manifestations of the Uterine Layer. The papillae are simple extensions of the Uterine Layer and seem to have the same internal ultrastructure as the rest of the Uterine Layer. In our scheme, we will use the descriptive term Surface Papillae to refer to these and similar ornamentations. Curiously, the Outer Uterine Membrane between the Superficial Papillae of *H. huffmani* in our Figures 6B and 6D look quite different from those in fig. 2 of Žďárská *et al.* (2001) [125] and fig. 2 (left) of Huffman and Moravec (1988) [47]. However, the obvious micro-ridges in the Outer Uterine Membrane between the papillae in the latter references (*vs.* the smooth surface between Superficial Papillae in most of our SEM imagery) are probably caused by differences in drying protocols (the ridges probably being compression wrinkles in the Outer Uterine Membrane following dehydration of the underlying Inner Uterine Layer), since the eggs from all three image sets came from the same population.

**Figure 7:**
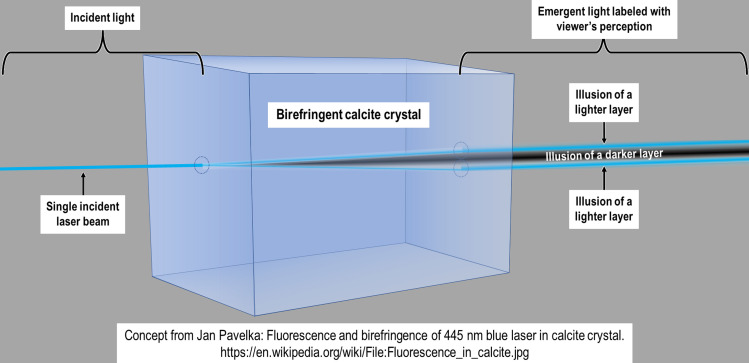
A simple example of a collimated beam of light being split into two parallel beams by a birefringent material. The various beams are explained in the link but the important thing to understand is that this unexpected optical phenomenon is being produced by one monochromatic source of light incident on one geometrically simple but birefringent object. A trichinelloid eggshell is much more complex with thousands of protein “pipes” surrounding a birefringent core of chitin and with the pipes arranged parallel in layers and each layer rotated around a perpendicular axis by about 10° relative to the next. Adapted from Jan Pavelka: Fluorescence and birefringence of 445 nm blue laser in calcite crystal https://en.wikipedia.org/wiki/File:Fluorescence_in_calcite.jpg.

**Figure 8:**
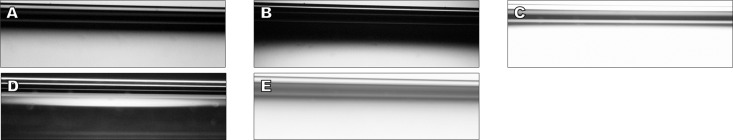
Mystery objects viewed under LM (see Section 4.3.5 when ready to assess diagnosis).

When the Superficial Papillae are viewed down-axis in LM whole mounts, they often appear to be sharply pointed (“spines”) or even open at the tip, but this is probably another type of optical illusion since clear SEM and TEM images show blunt papillae with no openings. Note also that TEM sections show no compositional or structural cause for the Superficial Papillae consistently appearing in SEM as white against a gray background (*i.e.*, Figure 6A), so the “white tips” probably represent electrical-charge artifacts. Like most other genera for which the Uterine Layer has been studied, the Uterine Layer of *H. huffmani* changes markedly from early embryonic to old and empty eggshells. Note the progression displayed in figs. 4, 7, 8 and 10 of Žďárská *et al.* (2001) [125], all of which occurred post-oviposition.

The spacing and shape of papillae in the Uterine Layer vary among *Huffmanela* species that have them. For instance, *H. huffmani* has Superficial Papillae appearing to be arranged in longitudinal or spiral rows (Figures 6A and 6B) while those of *H. banningi* [74] (from pl. IV, fig. 2 of Moravec and Campbell (1991) [76]) appear as in concentric circles, with both species having Superficial Papillae uniformly spaced and longer than wide. Some other species, like *H. oleumimica* [96] in figs. 14 and 15 of Ruiz *et al.* (2013) [96] and the *Huffmanela* spp. in fig. 2C–2D of Attia *et al.* (2021) [8], have stump-like Superficial Papillae that are wider than long and irregularly spaced. However, the “unevenly dispersed, minute, stub-like eggshell spines” reported for *H. oleumimica* may not be surface projections (the Uterine Layer and *Pellicula Ovi* are both smooth in all other LM and SEM imagery and drawings), but possibly refraction artifacts caused by cocci adhered to the surface of the Uterine Layer (some seem to be arranged in short strings). Techniques can also flatten otherwise elongate papillae, causing them to appear as stump-like (compare figs. 5D, E of Bullard *et al.* (2022) [23]).

There has been considerable speculation regarding what controls the development of the sometimes geometrically complex, regularly repeating structures seen in the Uterine Layer of some nematodes. Crofton (1966) [32] insisted that the cells of the uterine lining are involved in molding the complex ornamentations. Christenson and Jacobs (1950) [29] speculated that the “knobs” of the *Ascaris* egg were formed when droplets of mucoid substance contacted the *Pellicula Ovi* and congealed in place. Ubelaker and Allison (1975) [107] responded that the uterine layer of *Ascaris suum* [42] did not continue over the operculum, and therefore probably was not formed by a random deposition process from the uterine lining as Christenson and Jacobs (1950) [29] had suggested. However, *Aspiculuris tetraptera* has an operculum and a Uterine Layer, and while the Uterine Layer maintains its basic structure over the operculum, it is modified and much thinner over the operculum as in pl. 2D of Wharton (1979) [115]. Wharton (1979) [114] provided a persuasive argument, suggesting that the various complex geometries in the Inner Uterine Layers of some nematodes are the result of a self-organizing property that emerges from a quasi-crystalline beginning after the material had been deposited onto the *Pellicula Ovi* by the uterine lining, with the uterus not participating in the subsequent organizational process. Indeed, the latter theory is probably the only one that would work with *Huffmanela*, since very early, apparently zygotic eggs are oviposited into host tissues before the Uterine Layer has developed any structural complexity (Figure 3A), and most development of the exogenous and endogenous eggshell components of this species take place outside the mother worm and in contact with host tissues (fig. 7 of Worsham *et al.* (2016) [123]).

The Outer Uterine Membrane of even well-developed eggs of *H. huffmani* are sometimes seen to be in intimate physiological association with host cytoplasm as in fig. 7 of Žďárská *et al.* (2001) [125]. Therefore, the individual *Huffmanela* egg appears to be an intracellular parasite (see p. 593 of Moravec *et al.* (1998) [78]). However, eggs of some *Huffmanela* species appearing to be normal can also be found in interstitial spaces between host cells [91], so cytoplasmic contact is apparently not required for normal eggshell or larval development. Another issue that would complicate the intracellular parasite theory is that the Permeability Barrier Membrane, which would prevent any exchange of even the smallest organic molecules between the host cell and the embryo, is known to be completed in *C. elegans* just after anaphase II is completed [83]. If this is also true for *Huffmanela*, and its eggs are physiological parasites, then the eggs must spend some considerable time in an arrested state of meiosis I after oviposition into host tissue before the Permeability Barrier Membrane has formed, which is the only way the encased larva could draw any organic nutrients from the host tissue. Interestingly, Worsham *et al.* (2016) [123] found that eggs of *H. huffmani* do not begin to appear in an infected fish until 7.5 months post infection, but must spend another 2.5 months in host tissue before they can survive in the environment, suggesting that the genesis of the Permeability Barrier Membrane just might be delayed in *H. huffmani* for an extended time after oviposition.

According to Wharton (1979) [114], the Outer Uterine Membrane of several nematode species appears to be laid down first, and the Inner Uterine Layer is then somehow forced beneath the Outer Uterine Membrane by secretory cells of the uterine lining. Some species clearly have pores in the Outer Uterine Membrane and this seems like a feasible explanation for the secondary deposition of the subtending Inner Uterine Layer in those cases, but in *Huffmanela*, no pores have been reported in the Outer Uterine Membrane in any stage of development, rendering unlikely the conjecture that the Inner Uterine Layer of *Huffmanela* could be deposited under the Outer Uterine Membrane.

Sometimes, perhaps when the Uterine Layer of *H. huffmani* is stripped away by rough handling, SEM imaging shows branched strands of thin fibrils radiating from the remaining surface of some eggshells (Figure 6E). These fibrils appear to be remnants of the electron-dense fibers in TEM imagery of the Inner Uterine Layer (see dark lines embedded in random orientation in Figure 4) and may be homologous to embedded and superficial fibers or filaments described for some other *Huffmanela* spp., as in figs. 1G–J, 2 of Justine (2004) [55], figs. 4H–K, M of Justine (2007) [57], fig. 1 of Justine (2011) [58], and also in the Uterine Layers of distantly related species of other orders reported by other investigators [60, 117]. The fibrous uterine secretions onto the *Pellicula Ovi* of *H. filamentosa* [55] appear not to have been encased in an Outer Uterine Membrane or embedded in a matrix, and seem to have been secreted continuously as eggs pass through the secretion zone of the uterus; thus, the eggs get linked together in a chain, like beads of a necklace as in fig. 1G of Justine (2004) [55].

*Huffmanela* appears to be the first genus in the superfamily Trichinelloidea reported to have eggs with a uterine membrane. *Cystoopsis* may be another, as suggested by Huffman and Moravec (1988) [47] and fig. 130F of Moravec (2001) [75], but we have not seen original egg micrographs of sufficient resolution that would allow a determination. Since the outer membrane in drawings of *Cystoopsis* eggs clearly surrounds the egg and both poles, TEM would likely show that there is a *Pellicula Ovi* between this presumptive Uterine Layer and the Chitinous Layer, and thus would confirm that the outer layer of the *Cystoopsis* egg is a Uterine Layer. Studies of *Trichuris* eggshells have frequently called attention to a very thin layer of “particulate matter” on the outer surface of the *Pellicula Ovi*, but such layers, that have also been reported on the surface of some other eggshells do not appear to have been secreted by the uterus, and may be a result of the remodeling of the oocyte surface at fertilization or a remnant of its original surface.

### 4.3 Layer #2: The Chitinous Layer

#### 4.3.1 Overview

The Chitinous Layer is composed of chitin fibers that are tightly associated, in various species-specific degrees, with proteins. Its formation is initiated immediately after the *Pellicula Ovi* is formed following fertilization (within 5 min in *C. elegans* [67]). Chitin synthase multimers in the oocyte membrane synthesize and translocate chitin in between the *Pellicula Ovi* and the oolemma [103]. The material of the Chitinous Layer in *C. elegans* is not dense and compact like molded plastic, but more of a mesh-like material, sometimes with sponge-like spaces [103]. However, it is strong enough to resist normal deformation forces encountered in the reproductive tract, and maintenance of the physical constraint imposed by its shape (prolate spheroid) is apparently required for proper development of the larva [103]. The synthesis of chitin is stimulated until it is abruptly terminated when the chitin enzymatic system is suddenly internalized as the oocyte nucleus enters anaphase I [83, 103].

#### 4.3.2 Features of the outer surface of chitin

The Chitinous Layer is usually not visible from the outside of an undisturbed nematode egg due to it being formed internal to the *Pellicula Ovi*; however, the latter is so thin relative to the Chitinous Layer that fine-scale features of the external surface of the Chitinous Layer are often apparent via SEM. We have seen *H. huffmani* eggs having a perfectly smooth outer *Pellicula Ovi* and other conspecific eggs from the same batch and with the same technique history having prominent longitudinal rugae, sometimes manifesting through the Uterine Layer. The two SEM images in Figure 3, one smooth and one rugate, were from the same SEM field. We have also seen this in LM imagery of freshly freed eggs of *H. huffmani* in physiological saline. We do not know what causes this, but clearly such features have little or no diagnostic value and should not be included in descriptions unless they are consistent in frequency, orientation, and fineness/coarseness. Much finer longitudinal rugae may be more diagnostic [57] and fine transverse rugae have been described as consistent within a collection [30, 77, 95], and are probably more diagnostic.

Surfaces of some capillariid eggs present a different problem – the Outer Chitinous Layer is often formed into an open, woven mesh of strands sometimes supported by columnar “pillars” of Inner Chitinous Layer and producing interesting surface patterns in SEM that are also observable in LM. Grigonis and Solomon (1976) [43], in their TEM of undisturbed eggs of *Calodium hepaticum* [10, 73], which were sectioned while still embedded in liver tissue, clearly show that the mesh-like network of beams in the Outer Chitinous Layer and at least most of the “pores” between them are completely covered by a thin and delicate *Pellicula Ovi*. This exposed layer is often incidentally scrubbed off capillariid eggs during handling, resulting in artifactual patterns in apparent porosity (or lack thereof) across the exposed surface. Examples of this can be found in the SEM imagery and conclusions of fig. 3 of Magi *et al.* (2012) [65], Borba *et al.* (2021) [19], and Sukontason *et al.* (2006) [104]. Interpretation of the patterns described by Borba *et al.* (2021) [19] did not adequately take into account that the eggs in these studies had been sonicated and subsequently subjected to other handling and chemical treatments that had caused the *Pellicula Ovi* to be scrubbed off randomly in some areas of some eggs more so than in others. Note in the SEMs of *Eucoleus boehmi* [105] in Magi *et al.* (2012) [65] that the delicate *Pellicula Ovi* had been almost entirely scrubbed off from the eggs in all images, except for a small section apparently remaining over the lower-right quadrant of the Polar Plug in their fig. 3B. The SEM imagery of Sukontason *et al.* (2006) [104] was used to categorize eggs by percentage into three categories based on the porosity of the surface. They seemed to understand that the “adhering surface material” they referred to was actually the *Pellicula Ovi*, but still interpreted eggs with a partial covering as having only partial “beam and pillar network” in the chitin. Also, the dimpled “orange-peel” surface feature they referenced is an artifact of the SEM drying having pulled water out from under the *Pellicula Ovi* between the beams, causing the dimples.

In the Borba *et al.* (2021) [19] study on trichinelloid surface patterns, the word “surface” occurs 28 times, but the terms “vitelline” or “membrane” do not occur once, even though prior reports (figs. 3, 4 of Grigonis and Solomon (1976) [43], fig. 1B of Wharton (1980) [117]) have indicated that the actual surface of an undisturbed *C. hepaticum* egg is the *Pellicula Ovi* (vitelline membrane) and that it completely covers most “pores” of the type discussed in the Borba *et al.* (2021) [19] paper. A very clear example of patchiness in the *Pellicula Ovi* apparently resulting from incidental removal by vortexing is shown in fig. 1C, D of Macchioni *et al.* (2013) [63]. Thus, the six diagnostic types of surface patterns [19] described among the 12 species of capillariids are based on a mixture of mostly real features (geometry, weave patterns, feature dimensions and granularity of the exposed Outer Chitinous Layer) and artifactual manifestations (patterns of “porosity” with some random portions of the eggs still covered by *Pellicula Ovi*). There does seem to be consistent species-specific differences between the studied capillariids, but caution should be used in applying these patterns until a procedure is established for effective removal of the *Pellicula Ovi* without damaging the Outer Chitinous Layer, and the SEM work should be backed up with TEM to reveal the underlying structure that causes the surface features. Tangential ultramicrotomy sections through the eggshells of these same species and *C. hepaticum* could be very helpful. Borba *et al.* (2021) [20] followed the SEM study with a comprehensive machine-learning study of 28 species and 8 genera of capillariid eggs drawn from museum collections and based on LM-resolvable characters – this would be a perfect collection of eggs on which to perform a break-through TEM analysis across those 28 species. Until verified by TEM, it is speculation to assume that all capillariids with a weave-like pattern of “beams” exposed on the surface of chitin have pillars supporting the network at intersections of the beams, as in *C. hepaticum*.

#### 4.3.3 How many layers?

The Chitinous Layer is often quite thick in fully developed trichinelloid eggshells, and refraction halos caused by a variety of interacting optical phenomena often cause confused interpretations in LM studies of wholemount eggs. One such common misinterpretation has resulted in the “shell wall” (referring to the Rigid Eggshell Wall, Figure 2) of trichinelloid eggs often being described with “wall two-layered.” In LM views of trichinelloid eggs in wholemount, the Chitinous Layer actually does appear to be two- or even three-layered (see LM photos in our Figure 3B and in fig. 3 of Dill *et al.* (2016) [34] and fig. 6 of Traversa *et al.* (2011) [106]); but these are pseudo-layers caused by the birefringent nature of chitin and curvature of a refractile layer. That two-layered appearance, especially if the “inner layer” presents as a refractile hyaline lining that extends into the polar canal as in these images, is anatomically inconsistent with the TEM imagery of any trichinelloid eggshell we have seen.

To understand the birefringence phenomenon in its simplest possible expression, consider the model of a calcite crystal (which is naturally birefringent) in Figure 7. A single collimated beam of light entering one side of the crystal will be split into two parallel beams upon exit. The situation is much more complicated in light microscopy of a trichinelloid eggshell with light of mixed wavelength passing obliquely through a multi-layered sheet of birefringent chitin that is curved lengthwise and widthwise at varying radii of curvature, resulting in light and dark bands that add and subtract in complex ways to produce an unpredictable number of unexpected illusions of layers that are not really there.

However, one does not need to invoke birefringence to demonstrate the complexity of the situation when viewing, under LM, objects having curved but transparent walls, as does the nematode egg. Suppose an investigator was viewing the photomicrographs in Figure 8 and needed to determine how many different classes of objects are represented in these seven images, and how many compositional boundaries (layers, membranes, etc.) are in each image. Note that these are real images of real objects photographed through microscopes in our laboratory (images identified in Section 4.3.5).

Sadly, we are not the first to expose these oft-repeated misinterpretations of LM imagery (wall two-layered, etc.). As early as 1936, Schmidt (1936) [99] performed some ingeniously demonstrative experiments with chitin and polarized light and demonstrated how the views of chitin via transmitted light could produce dramatically different appearances and illusions under different conditions. Leon Jacobs then documented these illusory properties. Again in 1950, Christenson & Jacobs [29] pointed out that the “double-walled layering” (still being documented in the 21st Century!) is only an illusion. Monné and Hönig (1954) [72] provided a detailed discussion of the various light tricks played by the trichinelloid Chitinous Layer and gave a technical explanation for the illusions which they succinctly introduced with, “the birefringence phenomena exhibited by the [nematode egg] shell are rather complicated.” Inatomi (1960) [50] had noted in 1960 that the Chitinous Layer of *Trichuris vulpis* [40] eggs appeared to be distinctly double layered in whole mounts under LM, but never in corresponding TEM imagery, and warned that the “wall two-layered” appearance under LM must, therefore, be a refraction artifact. Again in 1979, Wharton (1979) [112] noted that much of the LM work on whole mounts of nematode eggs has been confused, and offered a warning against drawing anatomical conclusions about nematode eggshells from LM studies without ultrastructural [TEM] confirmation. The need for verification by TEM is particularly relevant when new or unusual structures are unexpectedly encountered in nematode eggshells studied via LM. Yet, well into the 21st Century, trichinelloid eggs continue to be described as having a two-layered “wall” (referring to what appears to be dark outer and hyaline inner layers) in studies based exclusively on whole-mount imagery from LM.

Despite the decades of explicit warnings and caveats in the literature reporting on nematode eggshells, investigators continue to include line drawings from their LM imagery that award false legitimacy to a non-existent “hyaline inner layer” lining the parietal surface of the Chitinous Layer and extending into the polar canal. See tab. 1 of Ruiz *et al.* (2013) [96] for extensive references to outer and inner shell-wall layers that have been reported or drawn by various authors in multiple species of *Huffmanela*, noting that in some cases the outer layer is darker, and in others, the inner layer is darker. We have seen these illusions and have even been able to reverse the light/dark:inner/outer pattern in the same egg by adjusting the sub-stage lighting and/or the level of the focal field.

**Figure 9:**
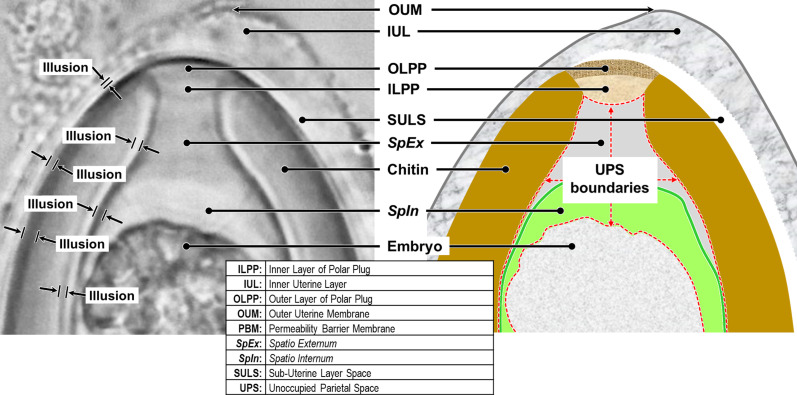
Egg of *Huffmanela huffmani* with mitotic embryo in comma stage as viewed under brightfield LM (from Figure 3B) showing several illusions commonly encountered in LM study of trichinelloid eggs in wholemount (left) and other real boundaries (right), some of which are rarely discernible via LM. Note optical illusion of light and dark linings of the Rigid Eggshell Wall extending up into canal. See Section 4.6.1 for TEM guide.

Consider the various illusions caused by refraction halos in the *Huffmanela huffmani* photomicrograph in our Figure 9 and the proper interpretation of the photo in the accompanying tracing. An excellent example of problems caused by combinations of such misleading optical illusions can be found in the description of the eggs of *H. oleumimica* by Ruiz et al. (2013) [96]. Their fig. 9 is a line drawing representing the salient anatomical features that appear to be present in the photomicrograph in their fig. 12 (wholemount of an early larvated egg). However, if one were to count along an equatorial radius in their fig. 12 from the left-most dark line to the egg’s center, there are no-less than 17 distinct light and dark traces that appear to be either membranes with intervening spaces or compositional boundaries running from pole to pole in that egg. At the time their manuscript was published, the authors had no reliable and widely accepted standards to go by to help them determine which of those lines should go into a drawing to represent real structures and which should be left out as illusions. Thus, since adults of *Huffmanela* spp. are rarely found, the morphological “features” that appeared (via LM) to be present in those eggs were used to provide some of the diagnostic characters on which to base the species description of *H. oleumimica*. Among those characters were an unknown number of optical artifacts that do not represent actual structures. The description of *H. oleumimica* was by no means superficial, but indeed, is included herein as an example because it is probably the most extensive and comprehensive LM study of *Huffmanela* eggshells ever reported and is based on perhaps hundreds of images and thousands of observed visual fields; and yet, because the drawings relied heavily on “characters” revealed via LM from wholemounts that were not verified by TEM, the drawings include an unknown number of artifacts. To complicate these issues further, the three technologies most often used in LM studies (basic bright-field LM, phase, and differential interference contrast (DIC)) will give noticeably different impressions of the same trichinelloid egg in the same wholemount. We have no doubt that *H. oleumimica* is a valid species; what we question is the validity of the anatomical conclusions inferred by the drawings, and their utility for future taxonomic description and identification. Three caveats should be noted at this point.

*Caveat 1:* The walls of some nematode eggshells actually *do* consist of two distinctive layers that can be visually distinguished via LM! For instance, the chitin of at least some capillariids is distinctly two-layered (*C. hepaticum* as seen in figs. 1a and 4 of Grigonis and Solomon (1976) [43]), and these two layers do sometimes resolve correctly under LM and should be drawn as such. We will refer to the outer of the two actual layers as the Outer Chitinous Layer (just under the *Pellicula Ovi*), and to the inner, often thicker layer (often lamellated in trichinelloids) as the Inner Chitinous Layer (Figure 2). It is important that future investigators understand that these two actual layers do not resolve in LM whole mounts into a darker outer layer and a refractile hyaline inner layer extending into the polar canal. Note also that the two layers in fig. 1a (LM) of Grigonis and Solomon (1976) [43] are consistent with their TEMs, but in the LM of fig. 1b, the inner hyaline “layer” labeled “a,” and continuing into the polar canal, is not a real layer, but a refraction halo that is inconsistent with their own TEM in fig. 4.

*Caveat 2*: The eggs of many other nematodes, including the trichinelloids such as *Huffmanela* and *Trichuris*, also have two layers of chitin: the Outer and Inner Chitinous Layers (Figure 2 and Section 4.3.4) as described in the TEM study of *H. huffmani* by Žďárská *et al.* (2001) [125] in their fig. 6. However, those two layers do NOT resolve clearly under LM in these worms, and never manifest as an outer, darker layer and an inner hyaline layer, with the latter extending into the Polar Canal – the latter always being an illusion. Therefore, when authors describe the eggshell “wall” (Rigid Eggshell Wall) as two-layered (based on LM) and draw a hyaline inner layer extending into the polar canal, they are referring to and drawing an optical illusion that, therefore, does not accurately represent any real structure in any trichinelloid eggshell for which we could find TEMs. Examples include a description of the eggshells of *Huffmanela* sp. in Attia *et al.* (2021) [8] as having, “… two layers. The outer brown and inner clear translucent….”; and egg drawings of *Cystoopsis* in fig. 133B, C of Moravec (2001) [75]; of *H. canadensis* in fig. 1 of Moravec *et al.* (2005) [77]; and others. This continues to happen despite TEM imagery of eggs of several trichinelloid species (fig. 9 of Preston and Jenkins (1985) [89] and fig. 6 of Wharton and Jenkins (1978) [120]) clearly showing that any actual layers internal to the main chitin “wall” must necessarily continue across the inner (proximal) surface of the polar plugs, and will never enter the polar canal between the polar plug material and the chitinous wall, as suggested in these and other photomicrographs and drawings therefrom. The situation with the *Huffmanela* plug appears to be somewhat different (Section 4.8.4); but nonetheless, there is obviously no hyaline layer running along the parietal surface of the Chitinous Layer and up into the Polar Canal to separate the wall from the plug.

*Caveat 3*: The Chitinous Layer of trichinelloid eggs, when examined by LM, almost *always does appear* to have a hyaline inner layer lining the inside of a darker outer layer, with that hyaline “layer” appearing to extend all the way up into the polar canals, forming the illusion of a hyaline lining in the canal. A good example of this is shown in an LM photo of a *Trichuris vulpis* egg in fig. 6 of Traversa *et al.* (2011) [106]. Add to that conundrum the fact that all investigators studying nematodes use some form of LM as their initial investigative technology. The question then becomes, “should drawings from LM studies show what the eggs actually look like under LM?” Perhaps so, but the misleading refraction halos should be called out for what they are – illusions, instead of describing the wall as two-layered, based on misleading LM imagery. It would be better to report, “walls of wholemount eggs appearing as two-layered under brightfield and phase microscopy.” All drawings of trichinelloid eggs based on LM should be qualified as such. Better still, omit drawings of LM wholemount eggs altogether, and include instead several photomicrographs of a representative egg at sequential optical planes from surface to mid-sagittal, with the diaphragm open to reduce depth of field. Such photos are more informative of what the next investigator will see when looking at eggs of the species via LM, while even good drawings based on bright-field LM study will be misleading, at best. If drawings are made, it might be better to use the vector-based strategy used by Justine (2004) [55] for *Huffmanela* eggs. The resulting drawings might seem oversimplified, but they do not include optical artifacts.

Although thin sectioning reduces the magnitude of the refraction problems by reducing the amount of curvature the light must pass through, the birefringence of the Chitinous Layer is still sufficient to give false suggestions of layering in the eggshells (*i.e.*, fig. 3 of Dill *et al.* (2016) [34], apparently based on paraffin microtomy). It is doubtful that a hypothetical TEM of an egg from Dill’s collection would confirm the apparent sandwiching of a layer of lighter brown chitin between two darker brown layers, as appears to be happening in almost all these eggshells.

#### 4.3.4 Clarified findings from TEM

Žďárská *et al.* (2001) [125] reported that the Chitinous Layer of the *H. huffmani* eggshell is composed of three sub-layers that are of visibly different textures in TEM imagery (in some regions of some eggs at some planes of section). The outer-most layer was dubbed the “external homogeneous part,” then the thick “middle lamellar part,” and finally the “inner net-like part.” We now know that the production of chitin is halted simultaneously with the release of cargo from oocyte cortical granules to form Layer #3 [83]. There is apparently some overlap of these depositional and remodeling processes in the assembly of most nematode eggshells, and Layer #3 is being produced by cortical granule cargo while the chitin layer is consolidating, and that could create an intermingling of the chitin with the Electron-dense Parietal Coating (Figure 2 and Section 4.4.2) of the inner surface of the Rigid Eggshell Wall. After reviewing our own TEMs of eggs from the same population and the TEMs of other reports on eggshells of other orders, we see no justification for Žďárská’s inner net-like chitin to be counted as a separate layer of chitin.

The material of the Electron-dense Parietal Coating also appears to penetrate into the parietal chitin to some degree in almost all eggshells we have seen of all orders, resulting in a radial gradient of electron density from dark to light centrifugally and causing a smudgy, indistinct boundary between the Electron-dense Parietal Coating and the Inner Chitinous Layer, in contrast to the distinct boundary between the Electron-dense Parietal Coating and the *Spatio Externum* (Figure 2 and Section 4.5.1). This could either be caused by the intermingling mentioned above or by some of the Electron-dense Parietal Coating material being mobilized by solvents and OsO_4_ during specimen processing [12]. These processes might be what causes the electron density gradient usually associated with the boundary between the Electron-dense Parietal Coating and the Inner Chitinous Layer; thus, the gradient could possibly be a pervasive technique artifact.

**Figure 10:**
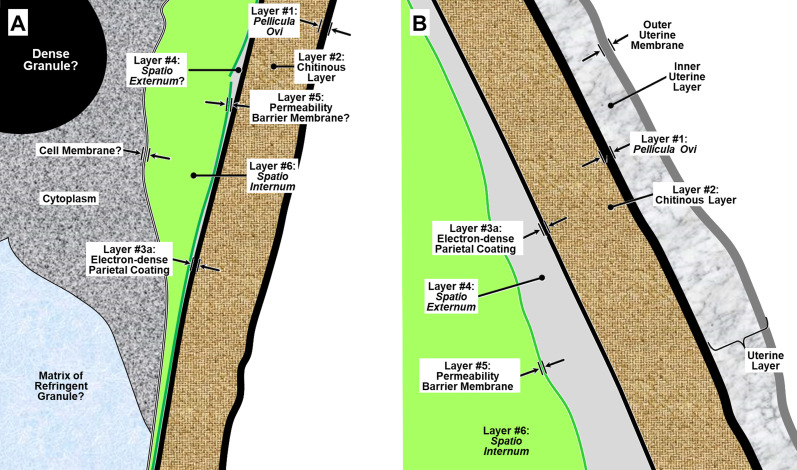
Interpretive tracing of (A) fig. 1a, (B) fig. 1d from Perry and Trett (1986) [86]; relabeled according to a new hexalaminar framework based on an adaptation of Olson *et al.* (2012) [83]; 1a *Heterodera schachtii*, 1d *H. glycines*.

The first two layers of chitin (the Outer and Inner Chitinous Layers; Figure 2) perceivable in TEMs of most trichinelloids and some genera of other orders are quite real, are usually easily resolvable in TEM, and have correlates in eggshells of other trichinelloids as well as some other nematode orders for which we have reviewed published TEMs. The layering is, unfortunately, overlooked by most authors, even when obvious, because chitin is defined as a single layer in the classical trilaminar anatomical model. For instance, Mansfield *et al.* (1992) [66], in a study of the eggs of *Haemonchus contortus* [94] (a trichostrongyloid), clearly show in their fig. 1 and several other images that the chitin consists of two layers of differing electron density, and Bird and McClure (1976) [17] show in pl. 1A and 1B that *Rotylenchulus* and *Tylenchulus* (tylenchids) have two layers of chitin, but neither paper mentions this sub-layering. Occasionally authors will refer to an outer and inner layer of chitin and label their imagery accordingly, but rarely discuss the issue further. In the Perry and Trett (1986) [86] study of *Heterodera* spp. (Tylenchida), they did label two layers of chitin: an inner, thicker and electron lucid Inner Chitinous Layer and an outer, thinner, and electron dense Outer Chitinous Layer (their figs. 1a–1d). However, there is no evidence in any of their TEM images of a *Pellicula Ovi* external to what they labeled as Outer Chitinous Layer, and they proposed that the *Pellicula Ovi* “degenerates within the uterus after eggshell formation.” That could possibly work for their fig. 1a, but their fig. 1d shows what appears to be a bi-laminar Uterine Layer and does not appear to be subtended by a *Pellicula Ovi* between what they labeled as Outer Chitinous Layer and the Uterine Layer; thus, the degeneration they referred to would have to happen almost instantaneously without leaving a trace before the Uterine Layer was laid down over it. Also, Burgwyn *et al.* (2003) [25], in their study of *Heterodera*, could find no evidence of chitin in the same layer Perry and Trett had labeled as Outer Chitinous Layer, so Perry and Trett (1986) [86] probably mislabeled a thick and dark *Pellicula Ovi* in both species as Outer Chitinous Layer. Burgwyn *et al.* (2003) [25] suggested that the outer layer of the *H. glycines* eggs is a mucoprotein layer (also visible in their fig. 3, but unmentioned) that had been provided by the cyst of the plant host. However, this would not likely result in a regular bilaminar structure as seen in the referenced photos and is therefore likely a Uterine Layer – the bilaminar structure potentially having arisen as an emergent property after oviposition. The broken, irregular appearance of the presumptive Outer Uterine Membrane in both photos could be the result of rough handling during the grinding process that released the eggs from the plant cyst [25]. We offer, in Figure 10, a revised interpretation of the Perry and Trett (1986) [86] eggshell imagery of *Heterodera schachtii* [98] and *H. glycines* [49] based on our adaptation of the Olson hexalaminar framework.

The outermost sub-layer of chitin identified by Žďárská *et al.* (2001) [125] as the “outer homogeneous layer” of *Huffmanela huffmani* is comprised of a single, thin band of chitin (labeled OC in their fig. 4) between the *Pellicula Ovi* and the first lamella. This is the first chitin deposited by the oocyte immediately after sperm penetration [103]. This sub-layer of chitin in *H. huffmani* (Figure 4) is not homogeneous as implied by Žďárská *et al.* (2001) [125], but is a mosaic of varied electron density with fibers that seem to be randomly arranged in a matrix with no evidence of lamellar fine structure, and so in our terminological scheme it is simply named the Outer Chitinous Layer. This layer is apparently homologous to the weave-like or mesh-like outer chitinous layer visible in SEMs of some other trichinelloids [19, 43, 104].

**Figure 11:**
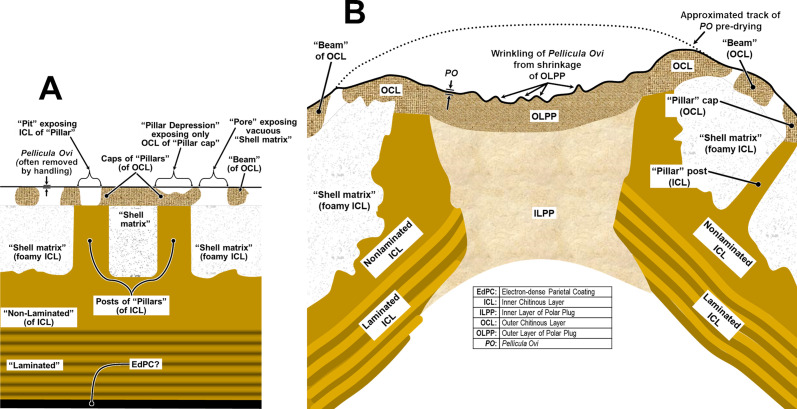
Tracings of TEM imagery of the *Calodium hepaticum* eggshell from Grigonis and Solomon (1976) [43]. (A) From their fig. 2a showing a reinterpreted and relabeled schematic representation of the authors’ interpretation of what they saw in their TEM imagery; (B) tracing (reinterpreted and relabeled) of their fig. 4 of a midsagittal TEM section of the polar region.

The Chitinous Layer of *Calodium hepaticum* is particularly intriguing, and appears to have three structurally different layers, as shown in fig. 4 of Grigonis and Solomon (1976) [43] (and our interpretive tracing in Figure 11B). The anatomical model they used to label their TEMs is drawn schematically in their fig. 2. The anatomical model they drew of this meshwork (their fig. 2B) suggests a geometrically regular arrangement of the beams that is not supported by any of the SEMs of other authors (see fig. 1C, D of Macchioni *et al.* (2013) [63]), which show more or less random weaving of strands (“beams”) of different thicknesses. Note that in the anatomical model of the outer chitin of *C. hepaticum* in fig. 2B of Grigonis and Solomon (1976) [43], the “beams” and “pillars” were interpreted by them to be composed of dissimilar materials, which would correspond to the Outer and Inner Chitinous Layers, respectively. After some study of their figs. 3, 4 and 8, however, we noted that the caps of the “pillars” appear (in their fig. 8) to be composed of the same material as the “beams” (Outer Chitinous Layer in our Figure 11B) while the posts of the pillars appear to be composed of the same material as their “NON-LAM” layer (Inner Chitinous Layer), but only up to where the pillars flare out at the level of the beams. We present a revised and relabeled tracing of their figs. 2a and 4 in our Figure 11. Thus, the outer membrane of the *C. hepaticum* eggshell (labeled “OM” in their figs.) is the *Pellicula Ovi* (our Figure 2 and Section 4.1.4); the “beams” and “pillar” caps appear to be composed of Outer Chitinous Layer material; and all wall material internal to the woven mesh material appears to be composed of Inner Chitinous Layer (which includes the posts of the “pillars,” the pith-like “shell matrix,” and the “NON-LAM” and laminated sections), and the laminated section is lined internally by the Electron-dense Parietal Coating (our Figure 2 and Section 4.4.2). Note that the Electron-dense Parietal Coating is not discernible in their published TEMs (or those of Inatomi (1962) [52]), but was drawn in their schematic as a dark “inner membrane.”

In SEM views of the *C. hepaticum* surface when the surface has been removed by rough handling (see especially fig. 1D of Macchioni *et al.* (2013) [63] and the anatomical model in our Figure 11A), the surface appears to be randomly peppered with three general types of depressions: (1) shallow “pillar depressions” in the weave material of the Outer Chitinous Layer that do not penetrate to the Inner Chitinous Layer, (2) more distinct and deeper pits that seem to expose a flat layer of Inner Chitinous Layer (tops of “pillar” posts beneath the weave of the Outer Chitinous Layer), and (3) “pores” that seem to have no bottom because they expose a space above the pithy shell matrix. After studying the TEM imagery in Grigonis and Solomon (1976) [43], we have concluded that probably all of the open depressions, pits, and pores exposed in the SEM imagery of capillariids in Borba *et al.* (2021) [19], Grigonis and Solomon (1976) [43], Sukontason *et al.* (2006) [104], and others, were originally completely covered by a relatively smooth but delicate *Pellicula Ovi* at the time of oviposition.

It is important to note at this point that the porous and often geometrically complex Outer Chitinous Layer of some capillariids superficially resembles (under LM) the Uterine Layer of some other orders. In both cases, the thicker basal layer of the eggshell of undisturbed eggs is covered by a thin electron-dense outer layer. Compare, for instance, (1) the Outer Chitinous Layer of *Calodium hepaticum* (a trichinelloid) in fig. 3 of Grigonis and Solomon (1976) [43] with (2) the Uterine Layers of the oxyurids *Hammerschmidtiella diesingi* in fig. 1 and pl. 1C of Wharton (1979) [113] and *Aspiculuris tetraptera* ([81] in pl. 1B of Wharton (1979) [115]); all are generically similar in gross appearance, but that of *C. hepaticum* is completely different from the others in composition and origin. The best way the viewer can determine whether the imagery is of a Uterine Layer or an Outer Chitinous Layer in such potentially confusing eggshells is with TEM (or perhaps DIC), where, if one is viewing a Uterine Layer, there will be a uniformly thin membrane-like layer of uniform texture (the *Pellicula Ovi*) separating the overlying Inner Uterine Layer from the underlying Outer Chitinous Layer. Note that there is no such distinct layer in the boundary between the nonlaminated and laminated layers of *C. hepaticum* in fig. 6 of Grigonis and Solomon (1976) [43]. As stated earlier, the *Pellicula Ovi* is *THE* foundational layer to which all other layers of the nematode eggshell are referenced.

While the distinction between the Outer and Inner Chitinous Layers cannot be resolved via LM in most *Huffmanela* species, some species from sharks are an exception. The eggs of *Huffmanela* from sharks in figs. 6A, B of Attia *et al.* (2021) [7] and in figs. 1D–G, and 2B of Bullard *et al.* (2012) [24] have an Outer Chitinous Layer that is characterized by radial strands and is readily distinguished from the Inner Chitinous Layer via LM. We must restate for emphasis, however, that while the Outer and Inner Chitinous Layers are true, physical layers that are obvious in TEMs of some genera, and may be present even when not visually obvious, these sub-layers of chitin are NOT correlated with the illusion of an outer dark and inner hyaline layer that so often appear in photomicrographs and drawings from LM study of trichinelloid wholemounts, and which has led to the erroneous conclusion, “wall two-layered.”

The Inner Chitinous Layer appears to be laminar in TEMs of most thick-shelled trichinelloids and appears as alternating electron-dense and electron-lucid lamellae in all views. However, these are not actually alternating layers of materials differing in composition and electron density, as the name and appearance (and most of the literature) suggest. If one looks closely at a TEM showing banding in the Inner Chitinous Layer of a trichinelloid eggshell (Figure 4) and considers the chitin between the darkest parts of two adjacent bands – those dark bands are simply where the chitin/protein fibrils are perpendicular to the electron beam, and the intervening lighter regions between the dark bands are where the fibrils are parallel to the beam.

**Figure 12:**
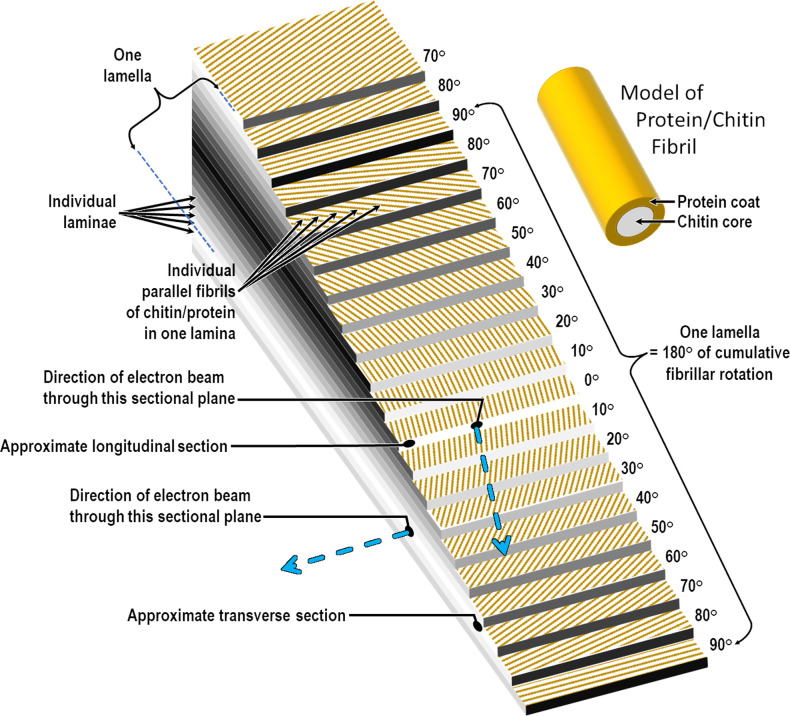
Anatomical model of Bouligand helical architecture of the laminated zone of the Chitinous Layer in trichinelloid eggshells (adapted from Wharton [111, 117]). Note that the laminae that appear dark in transverse section will appear light in longitudinal section and *vice versa*.

The chitin between the darkest part of one band and the darkest part of the concentric band next to it is referred to as one lamella and, in turn, consists of many extremely thin, true physical layers called laminae (see schematic in Figure 12). The chitin in the laminated zone of a trichinelloid eggshell does not consist of chitin molecules embedded randomly in a protein matrix. Instead, the chitin occurs in the form of short, straight microfibrils of chitin about 3 nm in diameter completely surrounded in a tube of protein, with the diameter of the entire fibril being about 10 nm [117]. The fibrils of the laminated zone are oriented in a plane that is approximately tangential to the curvature of the *Pellicula Ovi* above it. A layer of parallel fibrils one-fibril thick constitutes a lamina. The laminae are stacked on top of each other except that all the fibrils of one lamina are oriented about 10° off parallel relative to all the fibrils in the next concentric lamina in a progressive Bouligand (1972) [21] helical architecture, as explained in detail in Wharton (1978) [111]. The rotational angle (and the fibril metrics) are apparently species specific [111, 117].

Since pure chitin is rather electron lucid relative to the protein, a fibril, when viewed with TEM from the end appears as a dark circle with a light core (like looking down a pipe to the light at the other end), but when viewed from the side appears as a dark line. Thus, each apparent lamella represents a stack of actual laminae, with the parallel fibrils in each successive concentric lamina rotated slightly relative to the previous, for an accumulated total of 180° of rotation per lamella. The darkness or lightness of a lamina is determined by how the electron beam passes through protein *vs*. chitin, so when the orientation of the fibrils in a lamina is parallel to the electron beam, the lamina appears semi-lucid, but when perpendicular to the beam, it appears dark. When a transverse section includes the entire collar, as in fig. 7 of Wharton and Jenkins (1978) [120], a double spiral is produced, but if a section is tangential to the surface as in fig. 14 of Wharton (1978) [111], the bands appear in a single spiral. Wharton (1978) [111] provides more in-depth explanations with references to literature on the exoskeleton of arthropods, which share Bouligand architecture with the eggshells of nematodes.

Note that there is substantial variation in the thickness of the dark *vs.* light bands in TEMs of lamellated trichinelloid Chitinous Layers. Before calling attention to such differences, one should note that there are several geometrically complex issues operating to cause such differences, including thickness of sections (artifactual), thickness of protein wall of fibrils *vs.* chitin core (genetic), etc. One should be intimately familiar with the esoteric geometry of Bouligand effects before drawing conclusions regarding what might appear to be species-specific attributes of the lamellated zone of trichinelloid eggshells.

#### 4.3.5 Optical conclusions

**Figure 13:**
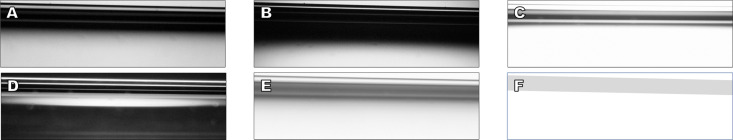
Same as Figure 8. A series of illusions created when a simple glass tube (tip of Pasteur pipette sloping down slightly to right) is viewed with light microscopy under varying conditions: (A) bright field compound scope @100×, substage condenser up; (B) same as A but condenser down; (C) same as A but stronger light; (D) same as A but phase contrast; (E) same tube but under a dissecting scope @40× and substage mirror at 45°; (F) is a drawing of the actual situation used to generate images A–E, with the gray bar in F representing the exact position of the compositional boundaries that generated A–E.

Figure 8 was presented earlier (Section 4.3.3) as a set of mystery images without explaining how many objects of what kinds had been imaged; the figure is reproduced below with the key in Figure 13F. All imagery therein except for F is one side of a straight, thin-walled tube of clear glass. Figure 13F is a drawing showing where the only two glass/air and air/glass boundaries actually are. If one were to purposely view the tip of a Pasteur pipette under a compound microscope using LM, the mind would ignore the various parallel light and dark lines in images A–E because it knows it is observing a thin-walled tube of transparent but refractile material; thus, there could only be two actual compositional discontinuities (air/glass and glass/air) and no black lines in the structure. However, if one is presented first with the light micrographs in Figure 8 and then challenged to determine how many different structures are being imaged and the actual number of compositional discontinuities (“layers”) in the walls of each structure, even if told that the first four were of the same object at the same scale, the analytical mind is highly unlikely to propose that a single-layered thin glass wall of uniform composition is being represented in all the images.

So, now that the reader knows there are only two air/glass boundaries in the “wall” of this transparent object, suppose a researcher had to rely on one or more of these images to determine the thickness of the wall. That would require choosing which dark or light “layers” represent real compositional boundaries. Image F in Figure 13 is a drawing that shows exactly the thickness of the structure imaged in A–E. How accurate would the drawings or measurements of the mystery object have been using bright-field LM or phase?

Now consider the same challenge, but with the walls consisting of an unknown number of concentric compositional boundaries between transparent materials having differing indices of refraction and with some materials birefringent – the problem of accurately representing the physical structure of the object being imaged by brightfield LM becomes almost intractable. It should now be clear to the reader that few compositional or even structural boundaries in a trichinelloid eggshell can be accurately identified by LM.

Thus, egg drawings of trichinelloid eggshells based on even the most meticulous study of whole mounts via simple bright-field or phase LM are almost always misleading. Differential interference contrast (DIC) imagery is more representative of eggshell reality than traditional bright field or phase LM. However, even DIC is fooled by thick, curved chitinous walls, especially those of trichinelloids with Bouligand rotational layering of lamellae. Even the maximum width of the egg and thickness of the Rigid Eggshell Wall are difficult to determine definitively with LM due to tricks played by the birefringent layers of chitin that are curved longitudinally and latitudinally.

The thickness of the Chitinous Layer of many trichinelloid eggshells presents peculiar interpretive problems that cannot be resolved by any new naming scheme. These problems arise when investigators attempt to interpret the structure of the nematode eggshell using traditional bright-field light microscopy (LM), which results in the incorporation of optical illusions into anatomical descriptions and drawings of trichinelloid eggshells. Almost all descriptions of eggs of *Huffmanela* (and most other trichinelloid species) are based on LM and some more recent descriptions include surface views via scanning electron microscopy (SEM). Because of optical properties and technique problems that are largely peculiar to trichinelloid eggshells, the features often incorporated into the egg descriptions are a virtually intractable mix of real structures, refraction artifacts, and technique-induced physical alterations. As will be shown below, even line drawings and morphometry specifications of trichinelloid eggs based on LM are invariably contaminated with artifacts which propagate into findings and conclusions.

With all that said, photographic images of eggs, even from bright-field LM, are much better than drawings based on LM for representing what the next researcher or diagnostician is likely to see when viewing eggs of the same species under LM.

### 4.4 Layer #3: Much confusion lives here

#### 4.4.1 Background

The most popular variant of the classical trilaminar anatomical model (Figure 1, Variant 1) consists of (1) the outermost *Pellicula Ovi* (formerly VL, vitelline layer), (2) the usually much thicker Chitinous Layer, and (3) the innermost layer containing lipids, and thought by some workers to be responsible for the permeability barrier. Many names have been given to this third layer, but the most frequently used have been Lipid-rich Layer or simply lipid layer. We will use the term Electron-dense Parietal Coating for all subsequent references to this layer and later show that it is part of Layer #3 in the new hexalaminar framework. There is widespread agreement (based on decades of accumulated findings from various angles and strong research support) that the Electron-dense Parietal Coating contains lipids in numerous species and provides the egg with at least some permeability resistance. Most authors who include the LrL (Lipid-rich Layer) in their TEM callouts have tagged as such the electron dense matter coating the parietal surface of the Chitinous Layer, but others have considered what is now known to be Layer #5 (Permeability Barrier Membrane in Figure 2 and “vitelline layer” in Variant 3 of Figure 1) as the lipid containing layer of the trilaminar anatomical model [28, 31, 38], and a few others tagged one of the concentric spaces of the Unoccupied Parietal Space (Layers #4 or #6 in Figure 2; see also Adamson (1983) [1], pl. 1c and 1d of Bird and McClure (1976) [17], pl. V.3 of Ibrahim (1982) [48]). All this has caused considerable confusion to accumulate in the nematode literature regarding the preeminently important function of permeability barrier in the nematode eggshell and has resulted in conflation of decades of important research due to a lack of terminological standards. Unfortunately, it gets worse.

The Olson hexalaminar framework, which emerged from in-depth studies of the development and composition of the *C. elegans* eggshell, specifies that almost all lipids between the *Pellicula Ovi* and the embryo/larva (of *C. elegans*) are in Layer #5 (the Permeability Barrier Membrane which divides the old peri-embryonic space into concentric outer and inner compartments), and that Layer #3 of the classical trilaminar model (the Lipid-rich Layer, now Electron-dense Parietal Coating in our new scheme; Figure 2) should be replaced in the *C. elegans* hexalaminar framework with a newly defined Layer #3, the chondroitin proteoglycan layer (CPG Layer) [83, 103]. Unfortunately, the *C. elegans* team did not address the findings from different laboratories that used a variety of investigative approaches (see Section 4.9.2) to demonstrate that Layer #3 contains lipids in many species. Now, reports coming out of other laboratories in the *C. elegans* Research Community are referring to Layer #5 as “*the* Lipid-rich Layer” (italics ours), a compound term previously used extensively in reference to Layer #3 and popularized as such by decades of previous work. While proponents of Variant 2 of the trilaminar model (Figure 1) also referred to a membrane (at Layer #5 of the hexalaminar anatomical model) as the Lipid-rich Layer, Variant 1 (which usually specifically assigns the Lipid-rich Layer label and lipid composition to an Electron-dense Parietal Coating applied to the Chitinous Layer as Layer #3) is much more commonly used among nematodologists outside the *C. elegans* Research Community. “The lipid layer consists of an electron-dense region *on the inner surface* of the chitinous layer” ([114]; emphasis ours). See also fig. 4G of Bird and Bird (1991) [16], fig. 1 of Mansfield *et al.* (1992) [66], fig. 1 of Wharton (1980) [117], schematics in fig. 4.7 of Wharton (1986) [119], although authors sometimes assigned the “Lipid-rich Layer” label to Layer #5 or combinations of Layers 3–5 of the hexalaminar model as in fig. 7A and fig. 8A, B respectively of Bird (1976) [15]. This already convoluted history associated with the Lipid-rich Layer has now been confounded by indirect suggestions by Olson *et al.* (2012) [83] in their landmark paper and in the Stein and Golden (2018) [103] review of the former that prior findings crediting Layer #3 as the Lipid-rich Layer of the eggshells of other species were misapplied to that layer and were based on low resolution extraction analyses. Such inferences (absence of lipids in Layer #3) are completely inconsistent with the many papers reporting contrary findings from rigorous research regarding the composition of Layer #3 in other species. Continuation of this practice (referring to Layer #5 as the Lipid-rich Layer and inferring that Layer #3 contains CPGs *rather* than lipids) will further complicate matters for researchers working with other species and trying to assemble a cohesive narrative regarding the various layers of nematode eggshells among diverse taxa. Below we will demonstrate that this CPGs *vs.* lipids situation is most likely not a matter of either/or. Indeed, it appears now that there are two sub layers at position #3 (at least in *C. elegans*), one sub-layer that always appears to contain lipids (Layer #3a) and sometimes another that appears to contain CPGs (Layer #3b), with both apparently resulting from the same exocytotic discharge of cortical granule cargo at anaphase I (Section 4.4.2).

After considerable study, multiple attempts at dialog with several researchers involved in nematode eggshell anatomy, composition, or development, and some occasionally contentious personal communications with researchers on both sides of this Layer #3 issue, we have come to two conclusions that might represent a resolution. Firstly, there are several sound arguments corroborating that, in most nematodes, the Electron-dense Parietal Coating (formerly Lipid-rich Layer): (1) exists as an entity intimately attached to the parietal surface of the Chitinous Layer; (2) contains lipids and usually proteins; (3) may serve as a partial permeability barrier, at least for larger molecules in at least some species; and (4) seems to be almost universally present in eggshell TEMs across the phylum, including, as it turns out, *C. elegans*. Secondly, we concluded that there are also good arguments that the CPG Layer: (1) exists just internal to the Electron-dense Parietal Coating (at least in *C. elegans*); and (2) that it is composed of mostly CPGs 1 and 2. However, that second conclusion must be tempered with the understanding that (3) there are virtually no arguments or data (at this writing) in support of there being CPGs at position #3 in the eggs of any other nematode clade (except for some otherwise unexplained fibrils just internal to the Electron-dense Parietal Coating in TEMs of some species); (4) the eggshell of *C. elegans* does have an Electron-dense Parietal Coating between the CPG Layer and the Chitinous Layer, although usually faintly stained (especially in Olson’s [83] imagery); and (5) many TEMs of *C. elegans* in the literature from other laboratories do NOT show a visible band-like layer similar to the distinct CPG Layer evident in Olson’s [83] imagery. Therefore, while these issues are being sorted out by future researchers, we will keep both layers at position #3 in our recommended modification of the *C. elegans* hexalaminar framework and will refer to the Electron-dense Parietal Coating as Layer #3a, and to the CPG Layer (when detectable) as Layer #3b.

#### 4.4.2 Layer #3a: the Electron-dense Parietal Coating

After a survey of the literature dealing with the hypothetical permeability barrier of nematode eggshells, much of which occurred prior to 1990, we have chosen the excellent TEM imagery and detailed descriptions of Foor (1967) [39] as the clearest and easiest to understand account of the processes that seem to be at work in the deposition of an Electron-dense Parietal Coating on the parietal surface of the Chitinous Layer of *Ascaris lumbricoides* [61]. Please note that we are using “parietal surface” in the general anatomical sense of the proximal (inner) surface of the wall of a cavity, as in the parietal peritoneum which lines the inner wall surface of the coelomic cavity of vertebrates.

As soon as the maternal chromosomes in the oocyte of *C. elegans* separate at anaphase I, chitin synthesis is terminated and a series of large membrane-bound vesicles that had accumulated in the cortical cytoplasm begin to express their cargos between the oolemma and the Chitinous Layer, thus separating the entire oolemma from the Chitinous Layer [13]. During the 20th Century, these large (often 3–5 μm) membrane-bounded multiphasic vesicles (often containing various layers and several distinct types of inclusions) were often referred to as “refringent granules.” We will follow recent literature and use the term cortical granules to collectively refer to all such vesicles that at some time or another migrate to the cortical cytoplasm of the oocyte and await a signal to discharge their cargos outside the oolemma. Also present in the oocyte cytoplasm of almost all nematode clades we have seen are smaller inclusions (about 1 μm) that are electron opaque and globular, and which are often referred to as “dense granules.” The TEM appearance of the cortical granules varies with species: those of *Ascaris lumbricoides*, as depicted by Foor (1967) [39], are different from those of *Heterakis gallinarum* [100] as depicted by Lee and Lešťan (1971) [60], etc.

**Figure 14:**
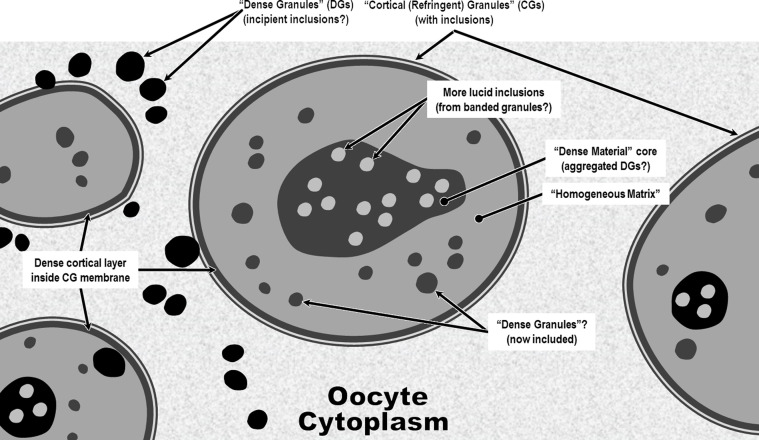
Tracing of an oocyte of *Ascaris lumbricoides* from fig. 6 from Foor (1967) [39] showing several almost mature cortical granules with diverse inclusions, apparently including dense granules, “core dense material,” which in turn includes less dense inclusions perhaps from banded granules, that appear as dense granules with a white belt (see Figure 15).

The content of the cortical granules of *A. lumbricoides* (Figure 14) consists mostly of a relatively “homogeneous matrix” of semi-lucid electron density with at least two and probably three types of small inclusions, and also a cortical layer of electron-dense material just under the bounding membrane. Note that the smaller dense granules that often appear as inclusions in the cortical granules seem not to be bounded by a bilaminar membrane in Foor’s very crisp TEMs, but may show faint evidence of a monolaminar membrane. Thus, it appears that the dense granules may be of a largely hydrophobic composition, and that they aggregate once inside the cortical granule to form a larger body that Foor referred to as a “core of dark material” inside the cortical granule. Embedded in the core are less-dense inclusions that may be from the lucid material that forms white bands around “banded granules” [39].

**Figure 15:**
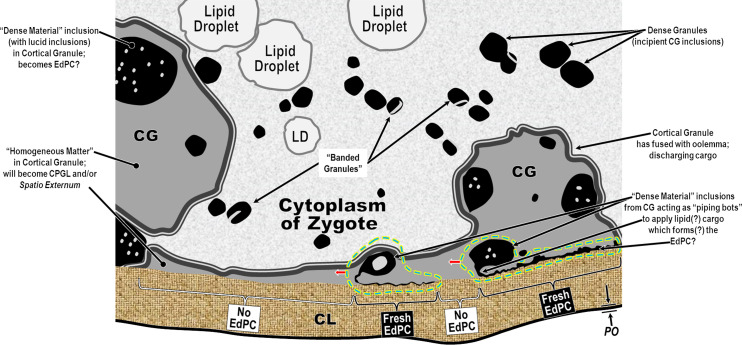
Interpretive tracing of fig. 15 from Foor (1967) [39]. Fertilized egg cell of *Ascaris lumbricoides* after anaphase I showing fusion of cortical granules with oolemma and discharge of cargos that separates the oolemma from the Chitinous Layer. Note how the different components of the cortical granules contribute to different layers, with the “homogeneous matter” forming the *Spatio Externum* while the Dense Material inclusions seem to be forming the Electron-dense Parietal Coating by applying their cargo as a dark coating on the exposed, freshly completed parietal surface of the Chitinous Layer.

When the cortical granules fuse with the oolemma, they eject their contents in between the oolemma and the parietal surface of the Chitinous Layer forming a fluid-filled compartment completely surrounding the oolemma, and which is destined to become Layer #4 of the new hexalaminar framework, the *Spatio Externum* (Section 4.5.1). The dense granules in each cortical granule then appear to migrate across the expanding space, and once they contact the chitin, are apparently triggered to begin “piping” their cargo onto the parietal surface of the Chitinous Layer, apparently in the same fashion as a cake decorator uses a squeeze bag and piping tool to apply calligraphy and decorations onto the surface of a cake. See fig. 15 of Foor (1967) [39] (traced in our Figure 15) for the most fortuitous and revealing imagery we have seen regarding this process. One can clearly see that the dense granules (now acting as autonomous “piping bots”?) appear to have left a trail of “dark material” behind them as they move along the parietal surface of the bare chitin (apparently moving from right to left in this image). In Foor’s [39] own words, “their dense particulate matter seems to collect underneath the chitinous layer.” That same electron-dense coating (that the dense granules appear to be painting onto the parietal surface of the Chitinous Layer) is discernible, to varying degrees, in almost all TEMs of Chitinous Layers we have seen, including most we have seen of *C. elegans* (figs. 2Cc, Cd of Bai *et al.* (2020) [9], fig. 4C of Benenati *et al.* (2009) [14], fig. 2C of Chen *et al.* (2017) [27], pls. V.3, V.4 of Ibrahim (1982) [48], and although rather faint, fig. 1C of Olson *et al.* (2012) [83]). This same process seems to have been captured in the formation of the eggshell of the ascarid *Syngamus trachea* (Montagu, 1811) Chapin, 1925 in fig. 7 of Bruňanská (1993) [22].

**Figure 16:**
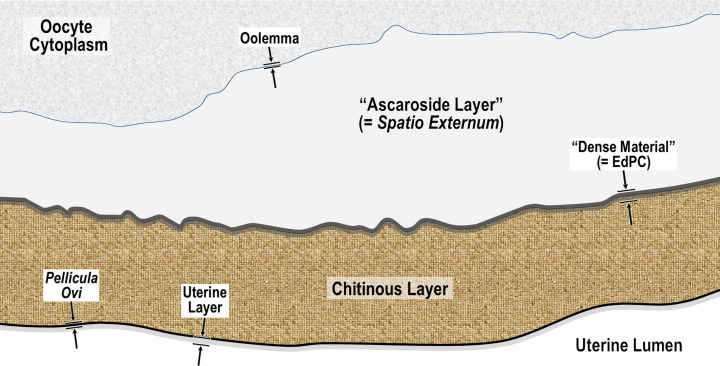
Interpretive tracing of fig. 16 from Foor (1967) [39]; a primary oocyte of *Ascaris lumbricoides* at approximately Telophase I.

**Figure 17:**
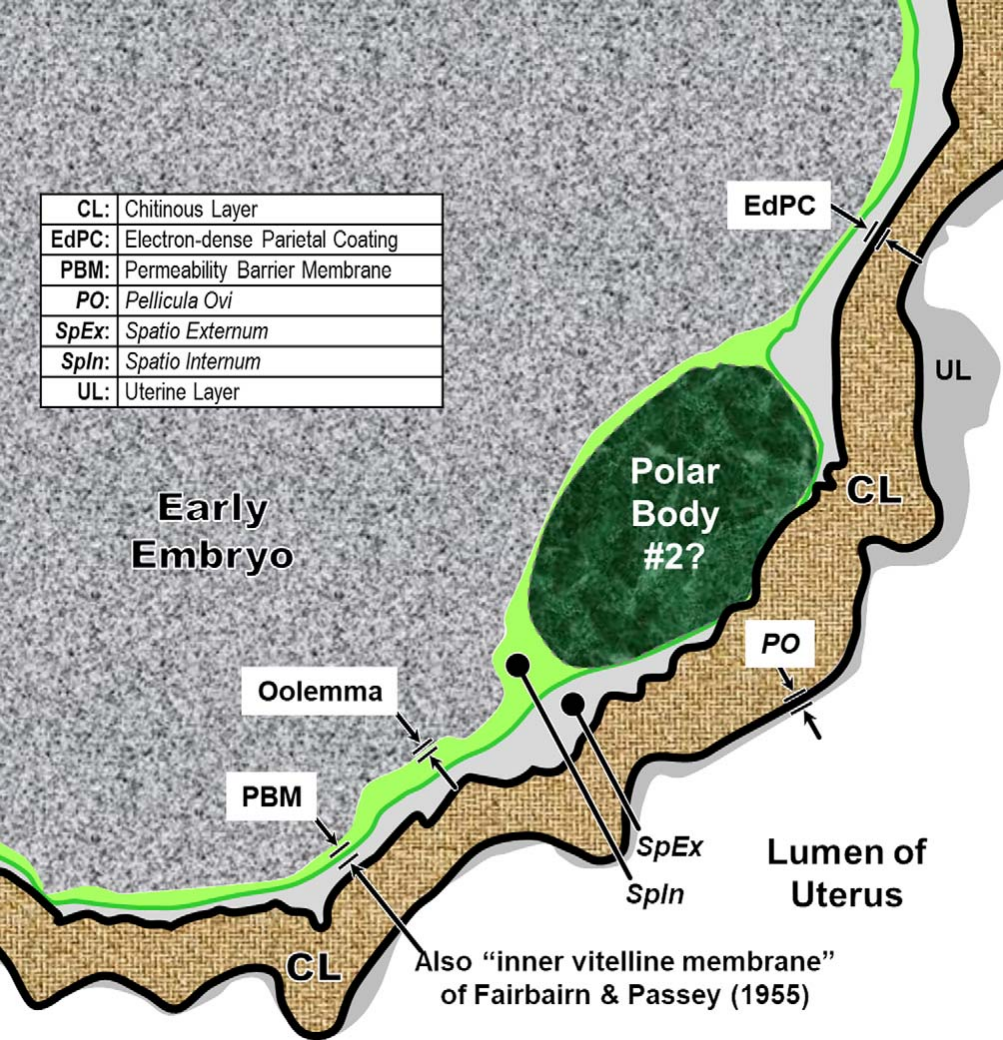
Interpretive tracing of completed eggshell of *Ascaris lumbricoides* from fig. 17 of Foor (1967) [39]. Please consult original and note that the ejected nucleus had been labeled as polar body #1 but it appears to be inside the Permeability Barrier Membrane (in the *Spatio Internum*) rather than in the *Spatio Externum* (which Foor had called the “ascaroside layer”). Since the polar body is in the inner of the two compartments, it should probably be considered as polar body #2 [83].

After the cortical granules have discharged all their cargo and the dense granules have applied their cargo to the entire parietal surface of the Chitinous Layer (but prior to anaphase II), one can see in Foor’s fig. 16 (see our interpretive tracing in Figure 16) that the cortical cytoplasm is no longer clogged with cortical granules, and most of the dense granules in the space we call the *Spatio Externum* (Layer #4) appear to have expended themselves coating the parietal surface of the Chitinous Layer with what Foor called “dense material” (others, “dark matter”), which in turn apparently becomes the substance of the Electron-dense Parietal Coating. Note that there are no Layers #5 or #6 in Figure 16, because those layers appear after anaphase II, by a yet unknown process. The eggshell development process for *A. lumbricoides* is shown completed in Foor’s fig. 17 (our Figure 17).

**Figure 18:**
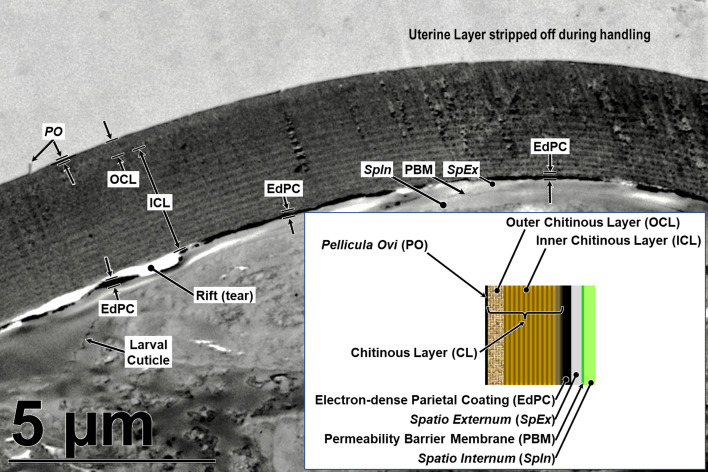
Transverse section of a larvated egg of *Huffmanela huffmani* showing the Electron-dense Parietal Coating layer having been separated from the chitin when the knife pushed the chitinous shell away from the rest of the section.

Žďárská *et al.* (2001) [125], in figs. 5 and 6 of their TEM study of *H. huffmani*, called attention to an “an inner electron-dense net-like” inner margin of the Chitinous Layer and labeled a stippled area next to it as an “electron-lucid lipid layer.” They also claimed that the lipid layer intermingled with the net-like chitin. The parietal surface of the Inner Chitinous Layer never appears to be net-like in our own TEM imagery of *H. huffmani* eggs from the same population, and the layer immediately internal to the Chitinous Layer appears as a thin, usually irregular, very electron dense layer closely adhered to the inner surface of the chitin (Figure 18), and is remarkably similar to the layer of “dense material” in fig. 16 of Foor’s [39] *Ascaris* imagery (traced in our Figure 15). Indeed, in almost every crisp osmium-stained TEM image of a developed nematode eggshell we have seen that includes the parietal surface of the Chitinous Layer, that surface has a coating or stain that is noticeably darker than the rest of the Chitinous Layer, and that coating corresponds positionally and visually to the layer apparently being applied to the Chitinous Layer of *A. lumbricoides* by the tiny “piping-bots” in fig. 15 of Foor (1967) [39] (traced in our Figure 15). This layer is also visible in most TEMs we have seen of *C. elegans*. The lining is less obvious in *C. elegans*, but it is there, and to the best of our knowledge, its presence there has not been addressed by the Olson *et al.* (2012) [83] team.

**Figure 19:**
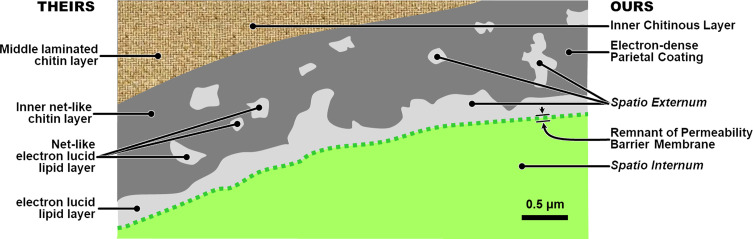
Interpretive tracing of TEM from fig. 5 of Žďárská *et al.* (2001) [125]. Permeability Barrier Membrane was apparently partially destroyed by solvents used in processing or by age-related deterioration in a dead egg. Labels used by Žďárská *et al.* (2001) [125] on left, our relabeling on right.

After more study of the *H. huffmani* TEMs in figs. 5 and 6 of Žďárská *et al.* (2001) [125], our own TEMs, and those of other published trichinelloid eggshells, all in consideration of the Olson *et al.* (2012) [83] hierarchical assembly model, we have decided that what Žďárská *et al.* (2001) [125] was probably referring to with “electron-dense net-like chitin” and “electron lucid lipid layer” in their figs. 5 and 6 (our Figure 19) is an Electron-dense Parietal Coating intermingling with the electron-lucid *Spatio Externum*. A similar spongy Electron-dense Parietal Coating seems to be present in fig. 1c of Perry and Trett (1986) [86].

Looking closely at fig. 5 of Žďárská *et al.* (2001) [125] (see interpretive tracing in our Figure 19 as a guide), one can see a diffuse remnant of what is probably the Permeability Barrier Membrane (Layer #5) separating the stippled material of the *Spatio Externum* (Layer #4) from the clearer *Spatio Internum* (Layer #6). The same stippling effect can be seen in the *Spatio Externum vs.* clearer *Spatio Internum* of *Trichuris trichiura* in fig. 2 of Appleton and White (1989) [6] (in which the *Spatio Externum* is labeled as lipid layer).

If *H. huffmani* does have an Electron-dense Parietal Coating, as suggested in our own Figures 4 and 18, why would Žďárská *et al.* (2001) [125] have confused it with an extension of the chitin? Probably because the electron density of the chitin of those eggs Žďárská *et al.* (2001) [125] examined was much darker than eggshells of any other nematode we have seen imaged, and this reduced any contrast differential relative to the Electron-dense Parietal Coating. That same reason may explain why they also missed seeing the *Pellicula Ovi* on the outer surface of the chitin and referred to the Uterine Layer as the vitelline layer.

**Figure 20:**
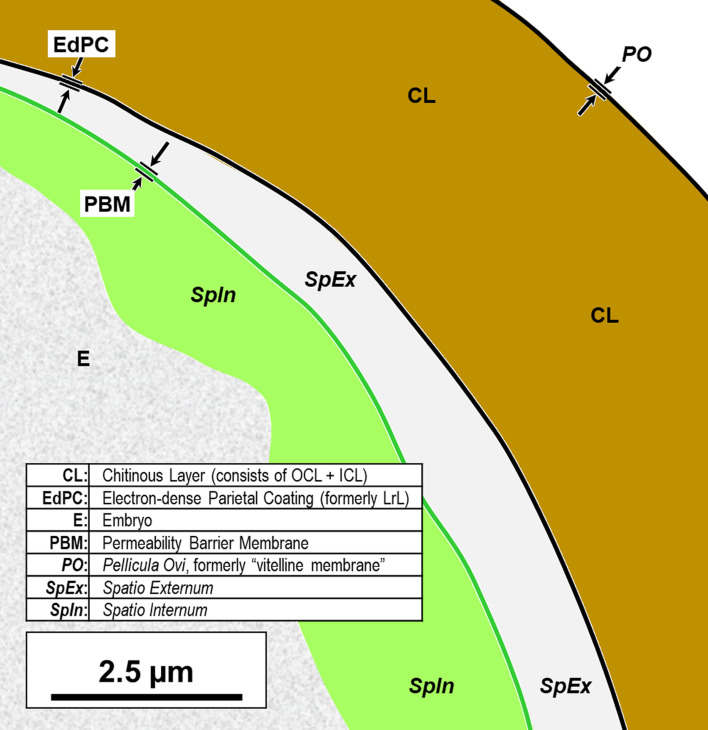
Interpretive tracing adapted from fig. 2 of Appleton and White (1989) [6] showing a transverse section of an intra-uterine egg of *Trichuris trichiura*. Note in original imagery the obvious compositional difference between the *Spatio Externum* and the *Spatio Internum*. Original labels replaced with terms from the proposed new terminological scheme.

**Figure 21:**
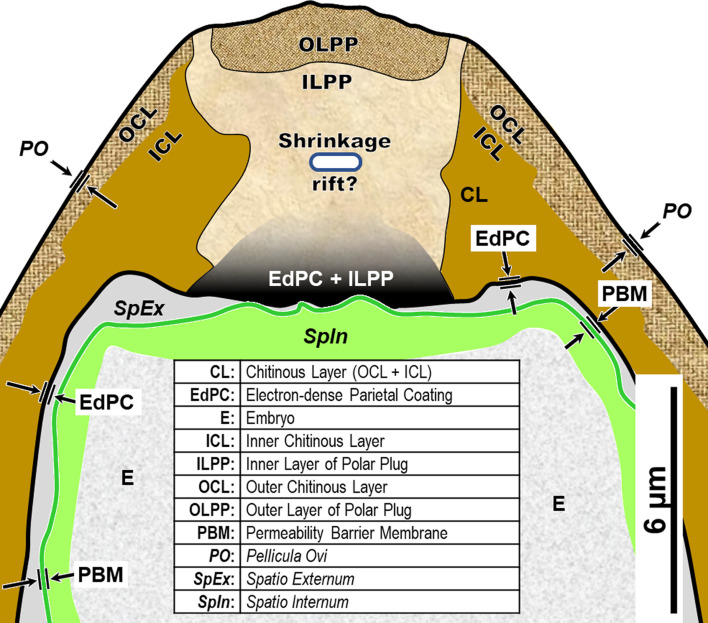
Interpretive tracing adapted from fig. 4 of Appleton and White (1989) [6] showing a paraxial section of the polar region of an intra-uterine egg of *Trichuris trichiura*. Note in the original image the textural difference between the *Spatio Externum* and the *Spatio Internum* and the electron density difference between the Outer Layer of the Polar Plug and the Inner Layer of the Polar Plug. Original labels replaced with terms from the proposed new terminological scheme.

In some of the eggshell TEM studies we reviewed, all six layers of the Olson anatomical model are clearly present, but the authors analyzing the images were apparently so strongly influenced by decades of standardization on the classical trilaminar framework that they inadvertently chose one of the four layers internal to the Chitinous Layer to label as the Lipid-rich Layer and did not address the other three structural layers that we can retrospectively see were clearly present in their imagery. One of the best examples of this is the Appleton and White (1989) [6] study of *Trichuris trichiura* [61]. The reader interested in developing a functional understanding of the new hexalaminar eggshell framework of trichinelloids is strongly encouraged to acquire a PDF of that paper and consult their figs. 2 and 4 and compare with our interpretive tracings in Figures 20 and 21. The authors overlooked an electron-dense layer coating the parietal surface of the Chitinous Layer that is equivalent to the Electron-dense Parietal Coating in position and appearance (what was then the Lipid-rich Layer) and instead labeled a layer that is positionally equivalent to the *Spatio Externum* as the “lipid layer.” They also overlooked a bold line (now known to be the Permeability Barrier Membrane) and the *Spatio Internum* between that line and the embryo. The layer traced by the black line in our tracings (Figures 20 and 21) most likely corresponds to the Electron-dense Parietal Coating seen in the same position in other species, and is the layer most authors of that time labeled as the Lipid-rich Layer (Variant 1 of Figure 1). Likewise, in pl. 6C of Adamson (1983) [1], an egg of the oxyurid *Gyrinicola batrachiensis* [110], one can see an unlabeled but obvious electron-dense and smudgy layer coating the parietal surface of the Chitinous Layer, and which almost certainly is the Electron-dense Parietal Coating in that it occurs at the parietal surface of the Chitinous Layer where it borders the *Spatio Externum.* Electron dense strands in the *Spatio Externum* were labeled as the lipid layer and probably contained lipids, but the much bolder and very electron-dense parietal coating of the Chitinous Layer (*i.e.*, the Electron-dense Parietal Coating) was not labeled. Please understand – this passage and others similar to it are not attempts to ridicule prior authors for what they missed; quite the contrary, it is intended to provide evidence sufficient to demonstrate that the trilaminar model (1) fails to accommodate all the layers that are usually clearly present in TEMs (2) has left the cited authors with no choice but to pick one of four layers to label as Lipid-rich Layer, and (3) should be replaced with a new model using terms that can unambiguously represent all those layers.

**Figure 22:**
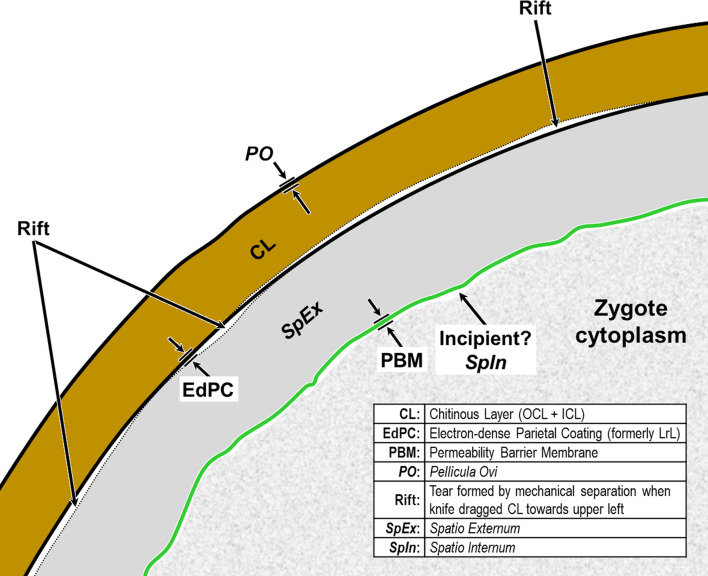
Interpretive tracing of fig. 2 from Wharton and Jenkins (1978) [120] showing a TEM of a “transverse section of the egg of *Trichuris suis* within the uterus of a gravid female” showing locations of various egg layers labeled under the new scheme. Note in original the obvious Electron-dense Parietal Coating and Permeability Barrier Membrane (neither addressed by the authors), and circular fibers in the *Spatio Externum* (in original).

**Figure 23:**
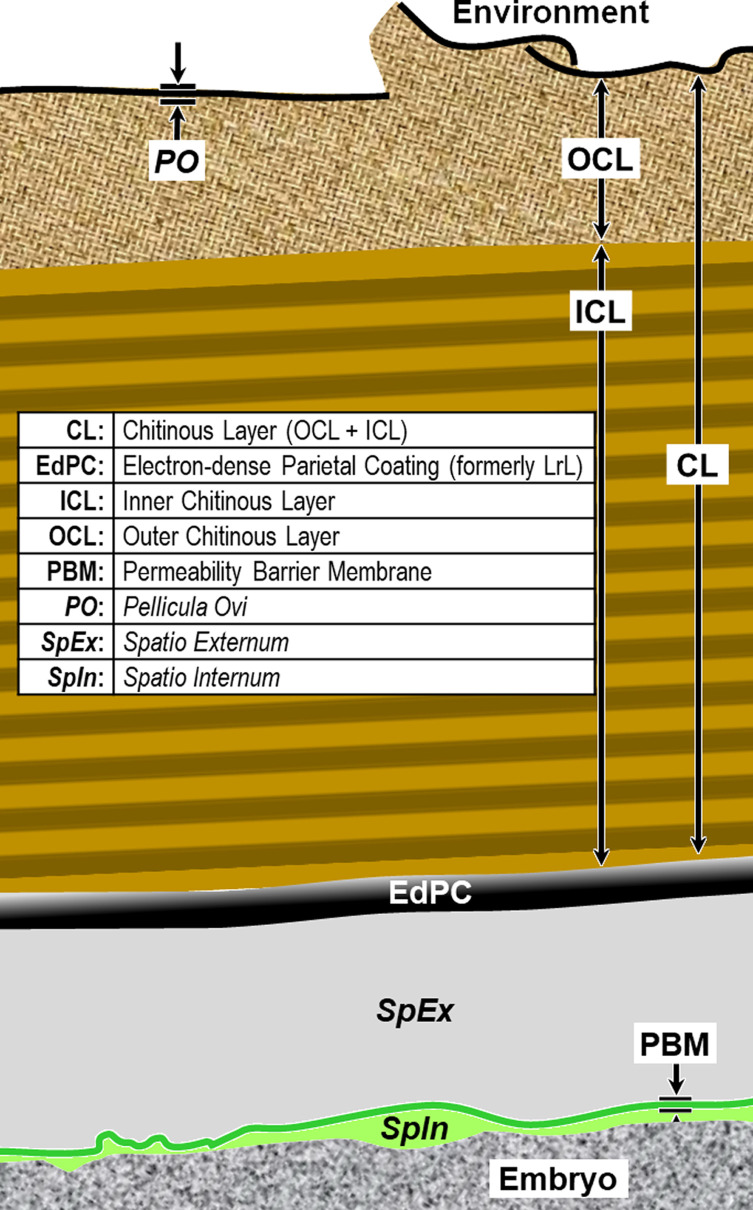
Interpretive tracing adapted from left half of freeze-etch replica of a transverse fracture of a *Trichuris suis* eggshell from fig. 9 of Wharton and Jenkins (1978) [120]. *Note:* (1) in the original imagery the distinct difference in texture between the Outer and Inner Chitinous Layers; (2) the thick band of tightly packed parallel fibrils (labeled “LL”) appears to be the *Spatio Externum*, with parallel fibrils oriented circumferentially; (3) careful inspection of the boundary between the *Spatio Externum* and the Chitinous Layer in the original reveals a distinct band of finely stippled texture that was unmentioned and unlabeled by the authors – most likely the Electron-dense Parietal Coating; (4) the broken dark line near the lower margin of the *Spatio Externum* would then be the Permeability Barrier Membrane but was not mentioned in the description; (5) beneath (internal to) the Permeability Barrier Membrane would be the *Spatio Internum*, which is shallow equatorially (as in Figure 22). Labels revised from original to reflect proposed new scheme.

In the Wharton (1979) [116] study of the eggshell of *Porrocaecum ensicaudatum* (Zeder, 1800) (an ascarid), Wharton’s fig. 4 is a transverse section showing a *Pellicula Ovi* over a thick Chitinous Layer with a bold and very distinct Electron-dense Parietal Coating, then a *Spatio Externum* clogged with electron-dense strands, then a partially disrupted Permeability Barrier Membrane surrounding what appears to be an expansive *Spatio Internum*. Because of the trilaminar expectation, everything internal to the Chitinous Layer was collapsed into “…irregular electron-dense material loosely attached to the inner surface of the chitinous layer.” In the Wharton and Jenkins (1978) [120] study of the eggs of *Trichuris suis* [100], their fig. 2 is a transverse section of an early egg prior to the formation of the *Spatio Internum* (see interpretive tracing in our Figure 22). It clearly shows an irregular electron-dense lining of the inner surface of the Chitinous Layer that had become separated from the chitin in several places like in our Figure 18, but the layer was not labeled by the authors in any of their images, including a freeze etch fracture in their fig. 9 (see our interpretive tracing in Figure 23). A similar situation with almost identical textures occurs in fig. 9 of Preston and Jenkins (1985) [89]. Inatomi (1962) [52] also indicated in Inatomi’s fig. 6 that there were thin membranes lining the outer and inner surfaces of the polar plug of *T. vulpis* (the *Pellicula Ovi* and Electron-dense Parietal Coating, respectively), but the inner lining was not further addressed (see Section 4.6.4). One author (whose identity will remain protected in anonymity) labeled three distinctly different hexalaminar egg layers with some variation of “Lipid-rich Layer” in different ultrastructure images of the same species in the same paper. These examples should provide sufficient evidence that it is time to re-examine all nematode eggshells from a fresh perspective. Indeed, while one could argue that it might be premature to generalize our adaptation of Olson’s [83] *C. elegans* framework across the phylum, there are many published eggshell images of diverse genera from several orders for which our adaptation of Olson’s [83] framework is a much more parsimonious fit than is the classical trilaminar framework, which is still in almost universal misapplication today!

#### 4.4.3 Layer #3b: the Chondroitin-Proteoglycan Layer (CPG Layer)

Olson *et al.* (2012) [83] provided indisputable experimental evidence that CPGs are expressed in between the Chitinous Layer and the oolemma by the cortical granules of *C. elegans*. They also determined that CPG production is required for Layer #3b to form, and that CPG-1 tends to remain attached to the Chitinous Layer and CPG-2 does not. This might suggest that the dense granules contain CPG-1 in addition to lipids, and that both CPGs-1 & -2, but primarily -2, are present in the more electron-lucid and fluid matrix of the cortical granules. However, the appearance and implied behavior of the dense granules in fig. 15 of Foor (1967) [39] (see our Figure 15) are what one would expect of a globule of lipid in a hydrated environment, and there are other persuasive indications that the dense granules and the Electron-dense Parietal Coating contains lipids in other nematode species. We offer a very preliminary speculation that the chitin-binding sites and hydrophobic/lipophilic groups on the CPG-1 molecule are being used to bind the lipids in the dense granules to the parietal surface of the Outer Chitinous Layer.

Since the basic structural sequence of the endogenous layers of the nematode eggshell seems to be conserved across studied groups in the phylum, the variation in appearance of the Electron-dense Parietal Coating from taxon to taxon and variations in findings regarding the genesis and composition of Layer #3 are probably not a matter of either/or, presence/absence differences. Instead, the variations probably have more to do with variations in species-specific adaptations regarding the relative proportions of the various ingredients of eggshells. Indeed, while the dark lining of the parietal surface of the Chitinous Layer (Electron-dense Parietal Coating) is very faint in *C. elegans* (and a few other species), the same layer is obvious in most species, and thick, bold, and extremely electron dense in still others. For instance, it has been demonstrated that the intensely bold electron density of Layer #3a in *Haemonchus contortus* (much darker than in *C. elegans*) is subject to radical modification by lipid solvents [66], a feature that would not be characteristic of a layer whose composition is not dominated by lipids, while Wharton (1979) [114] determined that lipids are almost completely absent from the faintly staining Electron-dense Parietal Coating of *Syphacia obvelata* (Rudolphi, 1802), a situation probably similar to *C. elegans*. Mansfield’s [66] experiments also explored the effects of Proteinase K on the Electron-dense Parietal Coating, and it had an even stronger effect on reducing electron density of the Electron-dense Parietal Coating, which would be consistent with the polypeptide backbone of CPG-1 being at least part of the protein component of the Electron-dense Parietal Coating. Olson *et al.* (2012) [83] noted that CPG-1 binds to chitin and speculated that it might be less soluble in the aqueous *Spatio Externum* than CPG-2. This might mean that CPG-1 has some hydrophobic side groups which would bind to the lipids of dense granules that randomly contact the CPG-1s, thereby drawing the lipids out of the dense granules and also moving them along across the surface of the hypothetically CPG-coated Chitinous Layer to form the Electron-dense Parietal Coating. However, it is important to remind the reader that very few TEMs of any other species we have seen show a definite band of material having semi-lucid electron density between the Electron-dense Parietal Coating and the fluid of the *Spatio Externum* that might be homologous to the band of CPGs in the *C. elegans* imagery in figs. 1 and 3 of Olson *et al.* (2012) [83]. Therefore, when the Olson team implied that previous authors were wrong in their claims that Layer #3 of most nematode species contains lipids (see Section 4.9.2 for direct quotes), they were probably drawing premature conclusions that could not be supported by their data, especially when they implied that the layer at position #3 that most authors referred to as the Lipid-rich Layer (now Electron-dense Parietal Coating) contained CPGs instead of lipids. The electron-lucid, band-like appearance of the CPG Layer in *C. elegans* eggs that Olson *et al.* (2012) [83] are referring to (in their figs. 1C, 3C) is a completely different layer from the Electron-dense Parietal Coating that the previous authors were working with when they concluded that the Electron-dense Parietal Coating contains lipids. Furthermore, we have closely re-examined every published TEM of the *C. elegans* wildtype eggshell we could find, and in almost all of those images there is a faint but consistent indication that *C. elegans* does, indeed, have an Electron-dense Parietal Coating on the parietal surface of the Chitinous Layer that is similar in appearance to the Electron-dense Parietal Coating imagery of other species, but fainter, and the Olson laboratory apparently did not notice it because it is so faint in their imagery. Indeed, in their interpretation of the POD-2 fatty acid interference experiment, they observed interference with the development of Layer #5 (the Permeability Barrier Membrane) but no interference with the development of the CPG Layer (Layer #3b) and inadvertently applied their conclusions to the Electron-dense Parietal Coating (Layer #3a) by concluding that Layer #5 is the layer where previous worker’s evidence of a Lipid-rich Layer should be assigned rather than to Layer #3.

On page 739 of Olson *et al.* (2012) [83], we find the following passage in the left column (bolding and square brackets ours):

Given our finding that **the inner eggshell layer** [Layer #3] is not the permeability barrier, we hypothesized that depletion of these [lipid-synthesis] genes affects formation of the permeability barrier [Layer #5] **rather than of the inner eggshell layer**. Consistent with this idea, depletion of fatty acid biosynthetic/modification enzymes did not affect chitin or mCherry::CPG-1 localization [the CPG Layer].

Unfortunately, they had overlooked the faint line in their TEMs of the *C. elegans* eggshell between the Chitinous Layer and the CPG Layer. That faint line is the *real* “inner eggshell layer” of the *C. elegans* egg, and they had erroneously conflated the “inner eggshell layer” of the trilaminar framework with their newly discovered CPG Layer, the development of the latter having not been affected by the blocking of fatty acid synthesis. Thus, the Olson team erroneously concluded that there is no evidence of lipids in Layer #3a (Electron-dense Parietal Coating) because Layer #3b (CPG Layer), which they had confused with Layer #3a (Electron-dense Parietal Coating), was not affected. This error was not caught by Stein and Golden (2018) [103] in their otherwise thorough review of the Olson team’s claims and that error is now being propagated by other laboratories participating in the *C. elegans* Research Community. Indeed, some of them are now referring to Layer #5 (the Permeability Barrier Membrane) as the lipid layer and even as the “lipid-rich layer” [9, 26, 35, 45], thus confounding the already confused situation by conflating their reference to the Permeability Barrier Membrane with the decades of literature referring to Layer #3 as the Lipid-rich Layer.

In a peripherally related series of experiments with the effects of the *seip-1* gene on the formation of the Permeability Barrier Membrane, Bai *et al.* (2020) [9] reported that *seip-1*-deficient *C. elegans* oocytes did not have a functional Permeability Barrier Membrane because the *seip-1* gene appears to be required for proper allocation of the various lipid species in the lipidome of *C. elegans*. However, in their fig. 2C, where they show TEMs of wild-type and *seipin-1* deficient *C. elegans* eggshells, we noticed that there is clearly a faint electron-dense layer between the Chitinous Layer and the CPG Layer that resembles the Electron-dense Parietal Coating of almost all TEMs we have seen of nematode eggshells, and Bai *et al.* (2020) [9] did not label it in their figures or mention it in their prose. The Electron-dense Parietal Coating is more obvious in the *C. elegans* TEMs of Bai *et al.* (2020) [9] than in those of Olson *et al.* (2012) [83], and one can see that the TEM of the Electron-dense Parietal Coating layer in the wildtype eggshell (fig. 2Cc) is crisper and bolder than in the TEM of the *seip-1*-deficient eggshell (fig. 2Cf) where the Electron-dense Parietal Coating appears to have merged with and diffused into the outermost portion of the CPG Layer. The apparent sensitivity to *seipin-1* of the processes that form that electron-dense layer between the Chitinous Layer and the CPG Layer could mean that the faint line does, indeed, represent the overlooked Electron-dense Parietal Coating of *C. elegans* and also that it does, indeed, contain at least some lipids in *C. elegans*. The layer may be vestigial in *C. elegans* but it is there, nonetheless (see also refs. to other *C. elegans* TEM imagery in Section 4.4.2). We hope this discrepancy will be addressed in future eggshell research on the species.

After struggling to find TEM evidence of the occurrence of the CPG Layer in other nematode eggs, we have decided it would be premature to suggest the abundant presence of CPGs in Layer #3 is universal across the phylum until this possibility is investigated thoroughly in several other orders. Additionally, we even wonder how permanent the CPG Layer is in *C. elegans*, because many TEMs we have seen of the *C. elegans* eggshell from other labs do NOT show any evidence of a band of CPGs between Layer #3a and Layer #4 similar to what is clearly shown and labeled as CPG Layer in the TEMs of Olson *et al.* (2012) [83] and some others. With all that said, we anticipate that future findings will ultimately demonstrate that CPGs and lipids are both contributing to species-specific attributes of Layer #3 and of the *Spatio Externum* across the phylum. We also anticipate that the Electron-dense Parietal Coating will not be found to serve as the main permeability barrier in the eggshells of most nematodes, and that the reports of Fairbairn and Passey (1955) [38], Olson *et al.* (2012) [83] and others assigning Layer #5 to the role of the principal permeability barrier will stand for most species. That may change, however, if species with a dark and bold Electron-dense Parietal Coating (such as *Haemonchus contortus*) are run through experiments similar to the Olson *et al.* (2012) [83] regimen. Much more research is needed on Layers #3a and 3b before we can expect breakthroughs in our understanding of how nematode eggs have resisted so many of our attempts to defeat them. We don’t even yet know the functional role the CPG Layer plays in *C. elegans*, nor details of the origin of the Permeability Barrier Membrane that is apparently responsible for most of the legendary resistance of some nematode eggs.

### 4.5 Layers 4–6: The Unoccupied Parietal Space

A quick glance at a fertilized nematode egg will reveal a deformable space between the Rigid Eggshell Wall and the embryo/larva. This space, which first appears at meiosis I will ultimately become the space that the larval worm actively displaces as it moves around inside the constraints of the Rigid Eggshell Wall (see video at https://youtu.be/M2ApXHhYbaw). We recommend the term Unoccupied Parietal Space (Figure 2) for referring collectively to this space and its contents at all stages of development.

Closer inspection of mitotic eggs reveals that the Unoccupied Parietal Space is divided into two concentric compartments by a thin, malleable, electron-dense membrane (*i.e.*, fig. 9 of Inatomi (1962) [52]; fig. 9 of Preston and Jenkins (1985) [89]; and fig. 2 of Appleton and White (1989) [6]. Several conflated, ambiguous terms (peri-vitelline space, peri-embryonic space, extra-embryonic space, etc.) have been used to refer to various combinations of the subcomponents of the Unoccupied Parietal Space, but perfunctory application of all of those terms in the classical and current nematode literature has caused nearly intractable conflation that continues through 2022. The confusion is compounded by the insistence of Stein and Golden (2018) [103] that all the sequentially formed endogenous components of the premitotic eggshell should be referred to as “layers,” including the fluid-filled spaces. This is understandable from an early ontogenetic context, but is unacceptably counterintuitive for use as a descriptive term for general nematodologists to apply to fluid-filled spaces and malleable membranes of postzygotic eggs. There is no general consensus regarding how to apply any of these terms to an embryonated/larvated nematode egg; thus, continued use of them to refer to the Unoccupied Parietal Space or either compartment therein would further confound the already confusing literature and clutter the returns of future keyword-based literature searches.

The membrane that physically separates the two compartments of the Unoccupied Parietal Space has been definitively demonstrated by Olson *et al.* (2012) [83] (and several previous authors) to also be of profound functional importance to the chemical ecology of the enclosed embryo/larva, preventing passage of all but small molecules from entering the fluid bathing the developing embryo/larva. Thus, it is important that future researchers have a definitive vocabulary with which they can unambiguously refer to these two spaces and the membrane that separates them. We will first offer new terms that are descriptive and have little or no historical baggage in the nematodology literature and then follow each with selected examples of conflations and misapplications of the older terms from the historical literature as justification for their replacement (see Figure 2 for reference).

We recommend Permeability Barrier Membrane for the membrane separating the two concentric compartments. Stein and Golden (2018) [103] had suggested that it should be formerly called a “Permeability Barrier Layer,” but we replaced the depositional noun “layer” in its name with the more descriptive noun “membrane” to unambiguously indicate that the term is (1) referring to a supple, foldable, membranous *structure* that provides that function, rather than just to the *concept*, and (2) to indicate that the term does NOT refer to the Electron-dense Parietal Coating (Layer #3a) since most previous attempts to assign the function of permeability barrier to some structure have attributed that function to the Electron-dense Parietal Coating. The term “permeability barrier” has been used conceptually to refer to a hypothetical barrier-to-diffusion thought to be located somewhere between the chitin and the embryo/larva. While the classical literature has usually attributed the function of permeability barrier to the Lipid-rich Layer (Layer #3a; Variant 1 in Figure 1), some researchers of the 20th Century have also referred to Layer #5 as the permeability barrier [38] (Variant 2 in Figure 1).

We also offer a pair of related Latin terms for the spaces on either side of the Permeability Barrier Membrane. We chose names that do not appear in nematode literature prior to this writing. *Spatio Externum* (*SpEx*) is recommended for the compartment external to the Permeability Barrier Membrane (the space between Layer #3 and Layer #5), and *Spatio Internum* (*SpIn*) for the space internal to the Permeability Barrier Membrane (between Layer #5 and the embryo/larva). For reasons that should become clear below, *Spatio Externum* replaces the “perivitelline space” of Olson *et al.* (2012) [83] and the “extra embryonic matrix” of Stein and Golden (2018) [103], while the *Spatio Internum* replaces the term “peri-embryonic space” used in both papers.

#### 4.5.1 Layer #4: The *Spatio Externum* (*SpEx*)

The outer and inner compartments between the Rigid Eggshell Wall and the embryo/larva and which are separated by the Permeability Barrier Membrane are often seen in TEMs to have obviously differing textures (figs. 2, 4 of Appleton and White (1989) [6], pl. 1c and 1d of Bird and McClure (1976) [17], fig. 9 of Preston and Jenkins (1985) [89], figs. 6, 9 of Wharton and Jenkins (1978) [120]) suggesting that they are composed of quite different materials. The outer *Spatio Externum* compartment appears to be filled with a readily deformable fluid or gel in some cases [53], and other times consistent in thickness and stuffed with circularly oriented fibers as in fig. 9 of Wharton and Jenkins (1978) [120] (labeled Lipid-rich Layer; see also Figure 23). Note how in a transverse section near the equator of a *Trichuris suis* egg in fig. 2 of Wharton and Jenkins (1978) [120] (traced in Figure 22), the *Spatio Externum* accounts for the majority of the Unoccupied Parietal Space and contains circularly oriented fibers. In their fig. 6, an axial section of the polar region (see tracing in Section 4.6.3), the fibers of the *Spatio Externum* seem to surround the inwardly bulging polar plug as a ring and appear as dots in cross section. Perhaps these fibers represent a band-like CPG Layer similar to that shown in fig. 1 of Olson *et al.* (2012) [83]?

#### 4.5.2 Layer #5: The Permeability Barrier Membrane

The source of the legendary desiccation resistance of nematode eggs has finally been definitively attributed, at least in *C. elegans*, to the thin and malleable membrane that separates the *Spatio Externum* from the *Spatio Internum*, and which forms at anaphase II of meiosis [83].

While Olson *et al.* (2012) [83] were the first to provide a demonstration of the protective properties of the Permeability Barrier Membrane at position #5 in live action, they were not the first to attribute these remarkable impermeability properties to a membrane surrounding the embryo that could only have been Layer #5, the Permeability Barrier Membrane. Fairbairn and Passey (1955) [38] experimentally removed (via digestion) the “hard shell” of *Ascaris* eggs and observed that the larvae remained alive and motile inside a “membrane” in which they could survive exposure to hydrochloric acid (2 N), nitric acid (2 N), sodium hydroxide (2 N), ammonium hydroxide (0.5 N), formaldehyde (3.3 N) for 24 h. No TEMs were provided of these eggs, but after reading their prose (Rogers (1956) [93], for instance), it seems that some authors of the early 20th Century had inadvertently assembled a variant of the trilaminar framework that conflated Layer #3a of the hexalaminar framework with Layer #5 of the hexalaminar framework into a composite third layer of their trilaminar anatomical model that sometimes included lipids suspended in Layer #4, the *Spatio Externum*. They seem to have been referring *in their TEM imagery* to an electron dense “coating” or “lining” on the parietal surface of the Chitinous Layer (which would be Layer #3a, the Electron-dense Parietal Coating; Figure 2), but were referring *in their experiments* to an inner “vitelline membrane” (which would be Layer #5, the Permeability Barrier Membrane; Figure 2). The ruggedly persistent membranous structure that Fairbairn and Passey (1955) [38] indicated had survived the removal of the “hard shell” could not have been the Electron-dense Parietal Coating because the latter surely would not have remained intact as a “membrane” following the destruction of the “hard shell” to which it would have been firmly attached (see rifts in Figure 18). Therefore, the “membrane,” that protected the enclosed embryo from the array of noxious and corrosive experimental fluids after the hard shell was destroyed, had to have been the Permeability Barrier Membrane at Layer #5, not the Electron-dense Parietal Coating at Layer #3a. Indeed, the latter would surely have crumbled and disintegrated by the further “shaking … for 48 h” after the hard shell had been digested. So, it seems that Fairbairn and Passey (1955) [38] should receive some credit for their discoveries regarding Layer #5 as the permeability barrier even though they may have attributed their experimental findings to the much more visible Layer #3 that they were seeing in their intact eggs. Actually, a very close inspection of fig. 12 of Rogers (1956) [93] will reveal a faint trace of a membrane about 1/3 the distance between the more obvious Electron-dense Parietal Coating lining the Chitinous Layer and the embryo, and this is probably a remnant of the membrane (Layer #5) that provided protection for the embryos of Fairbairn and Passey (1955) [38] in their experiments. They had missed seeing it because they did not know to look for it. In contrast, the Electron-dense Parietal Coating presents as a bold coating of irregular thickness on the parietal surface of the Chitinous Layer in TEMs of *Ascaris* (or any other species), but does not present as something that microscopists would refer to as a “membrane,” and does not appear as a structure that would have tensile strength sufficient to remain intact as the only thing preventing a motile larva from straightening out and escaping after the rigid outer shell had been removed.

Although convincing proof that Layer #5 is the permeability barrier has only been demonstrated for *C. elegans* (by Olson *et al.* (2012) [83]) and for *Ascaris lumbricoides* (by Fairbairn and Passey (1955) [38]), similar appearing membranes can be seen in the same position between two spaces in TEMs of other species: *Meloidogyne* (Tylenchida) pl. 1D and *Pratylenchus* (Tylenchida) pl. 1C of Bird and McClure (1976) [17]; *Calodium hepaticum* (Trichinellida) fig. 9 of Inatomi (1962) [52]; *Haemonchus* (Rhabditida) fig. 1 of Mansfield *et al.* (1992) [66]; *Heterodera* (Tylenchida) fig. 1d of Perry and Trett (1986) [86]; *Hammerschmidtiella* (Oxyurida) pl. 1B of Wharton (1979) [113]; *Syphacia* (Oxyurida) figs. 1B, 1C, 2C, and 3C of Wharton (1979) [114]; *Trichuris* (Trichinellida) figs. 6, 9 of Wharton and Jenkins (1978) [120] and others. It should be noted that some other published egg images of these same orders and genera do not show any indication of a malleable membrane suspended in a space between the Rigid Eggshell Wall and the zygote. The most likely explanation for this is that some published TEMs of nematode eggs are of premitotic eggs before these layers have formed.

Ideally, high magnification TEMs of eggshell structure prepared for publication should include an inset drawing showing the approximate anatomical context for the image, and should include, when possible, everything between the zygote/embryo/larva and the *Pellicula Ovi*. Images should ideally be labeled as either embryonated/larvated (=mitotic), or unembryonated (premeiotic or meiotic) when this is known.

The Permeability Barrier Membrane is the only component of the *C. elegans* eggshell that prevents the passage of large molecules and slows the passage of even very small molecules, including water [83]. In the imagery we have seen showing the Permeability Barrier Membrane in other species, it appears to be quite malleable, and in *Trichuris muris* collapses into a crumpled mass in the otherwise empty parietal chamber of the Rigid Eggshell Wall after the larva penetrates the Permeability Barrier Membrane, the polar plug material, and the *Pellicula Ovi* with its stylus and escapes (fig. 10 of Panesar and Croll (1981) [84]). Indeed, it has been demonstrated [84] that the internal volume of the Rigid Eggshell Wall of *T. muris* expands by 12% due to internal pressure increase just before eclosion, and then elastically returns to its original volume after the larva escapes. It is difficult to imagine the thin, flexible membrane of the Permeability Barrier Membrane having sufficient tensile strength to contain the osmotic pressure that later explosively ejects the larva of *T. muris* half way out of the eggshell [84]. Perhaps as pressure builds just prior to eclosion, the fluids in the *Spatio Externum* are forced out through the chitin and the Permeability Barrier Membrane expands against the parietal surface of the Rigid Eggshell Wall to stretch the Chitinous Layer outward. Incidentally, fig. 1C of Macchioni *et al.* (2013) [63] is a fortuitous SEM that provides a view into the empty polar canal of a recently evacuated eggshell of *C. hepaticum*. It shows a ripped and ragged membrane lining the internal half of the canal that was not mentioned by the authors. Surely this must be the remnant of the Permeability Barrier Membrane that the larva had punctured and then escaped through and dragged into the tight canal on the way out.

The formation of the *Spatio Externum* is initiated at anaphase I when the cortical granules fuse with the oolemma and discharge their contents between the oolemma and the Chitinous Layer. Part of this cargo apparently contributes to the Electron-dense Parietal Coating (see fig. 15 of Foor (1967) [39] and our Figure 15), and the rest apparently contributes to the fluid component of the *Spatio Externum*. The *Spatio Externum* of *C. elegans* (and probably most other species) receives the polar body of meiosis I [103].

The question of how Layer #5 is formed has not been visually established in any imagery we could find. Olson *et al.* (2012) [83] speculated that it might come from a second round of cargos from a different population of cortical granules in the oocyte, and Cao *et al.* (2022) [26] provided evidence that it is formed from lipid droplets containing ascarosides produced by the endoplasmic reticulum of the oocyte and segregated into functionally different populations by some sort of labeling process that depends upon the protein seipin. Layer #5 is first seen soon after anaphase II in *C. elegans* and constrains the 2nd polar body to within the *Spatio Internum* [83].

#### 4.5.3 Layer #6: The *Spatio Internum*; formerly the “peri-embryonic space”

The contents of the innermost compartment (the *Spatio Internum*) appear to be the most fluid-like of all “layers,” usually with relatively few inclusions discernible by TEM. This is the fluid that is displaced most readily as the larval worm moves around. The fluid accumulating here as development proceeds appears to be the residual fluid expressed by the developing embryo as it decreases in volume, rather than a definitive eggshell layer deposited by the embryo for some specific purpose. The second polar body (from meiosis II) is found in this space in *C. elegans* [83].

### 4.6 The hexalaminar framework as applied to the polar regions of the trichinelloid eggshell

As has been demonstrated thus far herein, available TEM imagery of the equatorial regions of trichinelloid eggshells, including our own *Huffmanela* work, retrospectively appears to correspond directly (except for the lack of an obvious CPG Layer) with our adaptation of the *C. elegans* hexalaminar anatomical model. However, *C. elegans* has no definitive polar plugs, and so we must use an additional level of inference to reinterpret the structure of the most intriguing and obvious features of trichinelloid eggshells – the polar plugs and canals.

The ultrastructure of the polar regions of several economically important trichinelloid nematodes received considerable attention during the latter half of the 20th Century. Unfortunately, several interpretive mistakes were made early on while attempting to apply the classical trilaminar framework, and errors in these papers have influenced subsequent interpretations of polar imagery, even well into the 21st Century.

The polar regions of trichinelloids for which we have seen TEMs are all variations on a common theme. Thus, we will refer to our interpretive tracing (Figure 21) of TEM imagery from the polar region of *Trichuris trichiura* adapted from fig. 4 of Appleton and White (1989) [6] and will reinterpret and relabel the structures in that image from the perspective of the new hexalaminar framework for embryonated eggs (Figure 2). The serious reader is advised to acquire a copy of that paper and examine the fine structure in the TEM image using our tracing in Figure 21 as a guide.

#### 4.6.1 The *Pellicula Ovi* of the polar region

**Figure 24:**
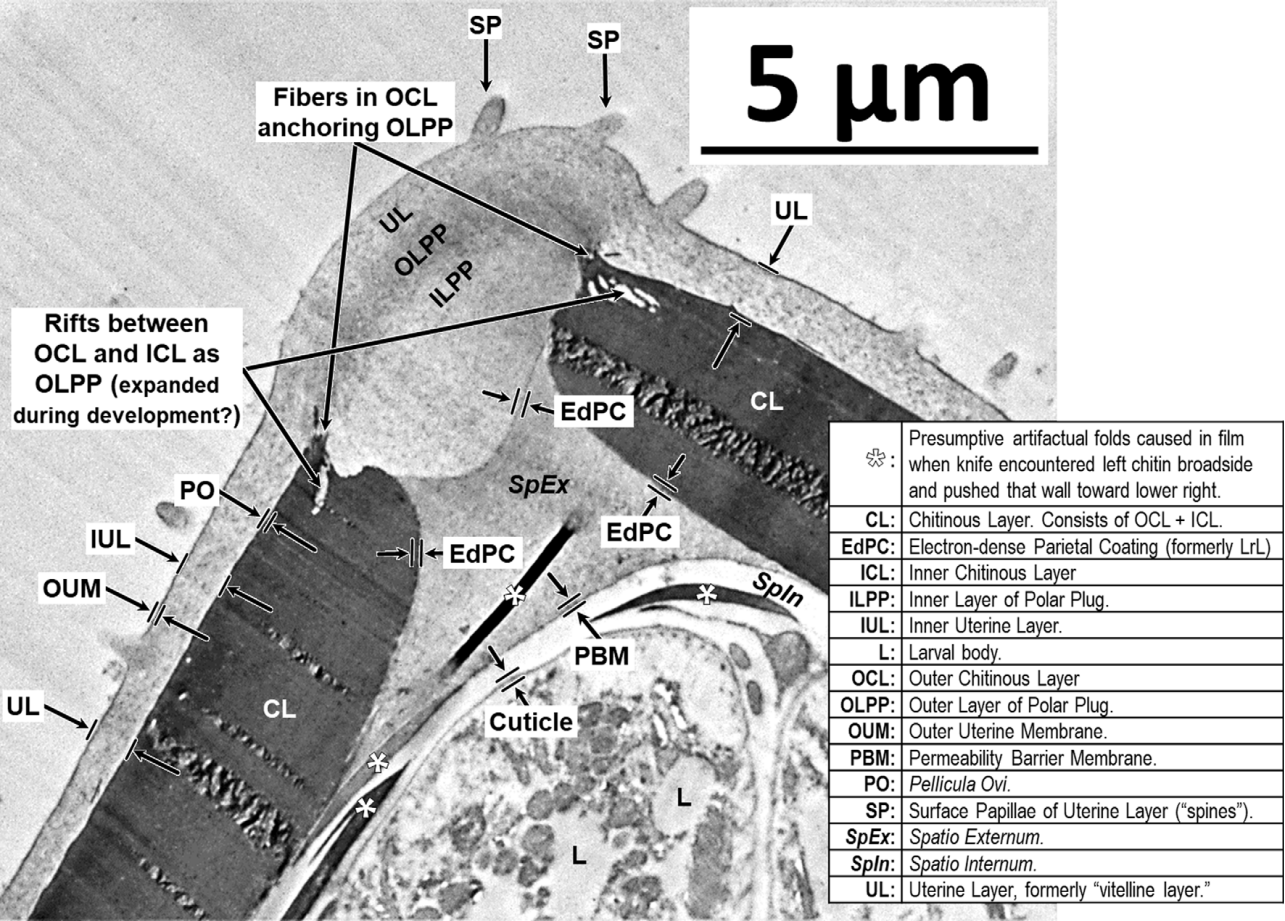
Mid-axial TEM section through polar region of *H. huffmani* egg with intact Uterine Layer. Note artifactually induced asymmetry of plug caused by knife pushing left wall toward lower right.

**Figure 25:**
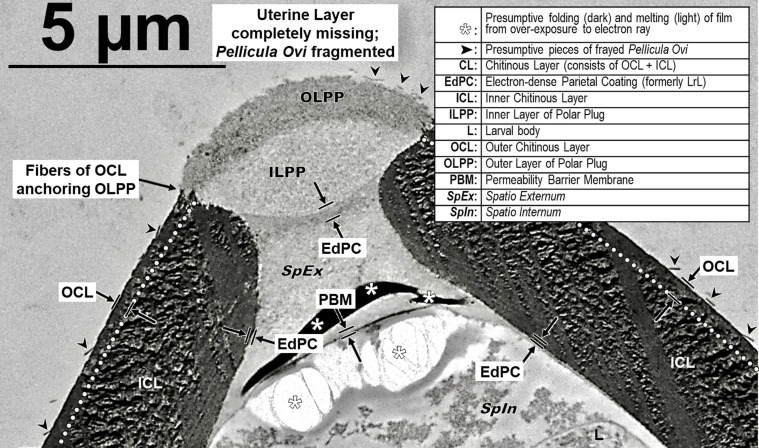
Paraxial TEM section *H. huffmani* polar plug region of an egg that has lost the Uterine Layer. *Note*: (1) the bowl-like boundary between the Inner Layer of Polar Plug and the Electron-dense Parietal Coating; (2) how innermost lamellae of Inner Chitinous Layer terminate before reaching polar canal; (3) complete absence of flaring among lamellae as they approach canal in contrast to *Trichuris*; (4) absence of any suggestion of a collar.

The *Pellicula Ovi* continues uninterrupted over the plug in trichurids and capillariids, as it does in other trichinelloids for which we have seen clear TEM imagery of the polar region. One curious aspect of the *Pellicula Ovi* in *H. huffmani*, is that, at first glance, it appears to be lacking over the polar plug in much of our TEM imagery (even when the overlying Uterine Layer is completely intact) and also in the TEM imagery of Žďárská *et al.* (2001) [125]. However, the *Pellicula Ovi* is actually there, as can be seen in Figure 24, but becomes less electron dense as it passes over the plug, and so it is easily overlooked. In Figure 25, the *Pellicula Ovi* has been fragmented into short pieces (arrow heads) by the knife during sectioning, both along the Chitinous Layer where it is dark and then when it changes to a lighter density as it passes over the Outer Layer of Polar Plug. This “bleaching” effect does not happen in the other trichinelloid imagery we have seen, and this may be due to chemical changes associated with plug formation that are unique to *H. huffmani*. Unfortunately, the TEM imagery of the *Huffmanela* sp. in Attia *et al.* (2021) [7] does not include sagittal views of the polar plug, so comparisons with *H. huffmani* polar plugs are not yet possible. In general, the *Pellicula Ovi* is much more easily discerned in the eggs of *Trichuris* spp. since they lack the uterine layer that *H. huffmani* possesses, and since the Outer Chitinous Layer of *Trichuris* is much less electron dense relative to the *Pellicula Ovi* than is the darker Outer Chitinous Layer of *H. huffmani*.

**Figure 26:**
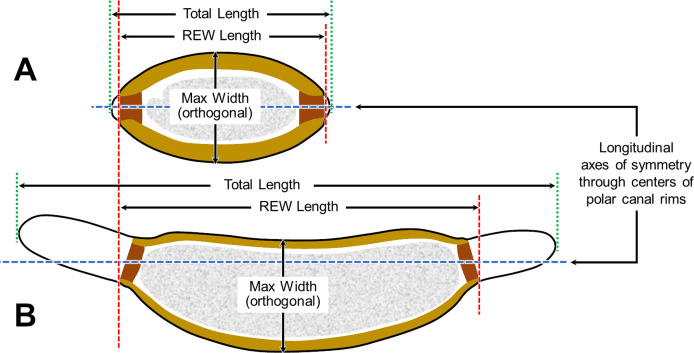
Interpretive tracing of egg drawings from: (A) egg of *Huffmanela huffmani* adapted from fig. 2E of Huffman and Moravec (1988) [47], showing the normal, barely extended polar plugs; and (B) an intrauterine egg of *Crocodylocapillaria longiovata* traced from drawing in fig. 1C of Moravec and Spratt (1998) [79] showing polar plugs that are normally extended so far as to account for a record 20–30% of total egg length. Horizontal dashed lines represent proper yaw alignment for ocular micrometer, while vertical dashed lines represent recommended rim-to-rim length of Rigid Eggshell Wall and vertical dotted lines represent the much less reliable total length from plug tip to plug tip.

In species with extended polar plugs, such as *Crocodylocapillaria longiovata* [79], the *Pellicula Ovi*, which is apparently the only layer between the plug material and the environment in trichinelloids, appears to have been stretched tight by hydraulic swelling of the Outer Layer of Polar Plug material (Figure 26B). Indeed, a series of photomicrographs of *T. muris* eggs in increasing readiness for eclosion are shown in figs. 3–6 of Panesar and Croll (1981) [84], and clearly indicate that the polar plugs increase substantially in volume, bulging radially and extending axially as the egg ripens.

#### 4.6.2 The Uterine Layer of the polar region

On eggs of *H. huffmani* where there is an intact Uterine Layer, it extends unaltered over the *Pellicula Ovi* of the plug as in Figures 6B and 24, and pl. III.2 of Huffman and Moravec (1988) [47].

#### 4.6.3 The Polar Plug and Canal

Like the equatorial region of the trichinelloid Rigid Eggshell Wall, the polar plug is also composed of a mix of chitin with protein but is much less electron dense than the chitinous wall of the polar canal. Monné and Hönig (1954) [72] give a detailed account of the differences between the composition of the Polar Plugs of trichinelloids relative to the rest of the Rigid Eggshell Wall. They provide evidence that the progressive change in color of the trichinelloid eggshell, from transparent to amber to dark brown and sometimes to black as the shell ages, is due to a predominance of quinone-tanned proteins with cross-linked cystine bridges. The Polar Plugs, which do not tan with age even though produced by a similar process at the same time, contain little protein but an abundance of polysaccharides. The wall of the Rigid Eggshell Wall tests negative for polysaccharides [72], probably because the chitin fibrils are apparently completely encased in protein.

The plug material of trichinelloids examined with TEM consists of two layers, the more electron dense and thinner Outer Layer of Polar Plug and the less electron dense and thicker Inner Layer of Polar Plug, with each apparently being derived from the corresponding sublayers of the Chitinous Layer (Outer Chitinous Layer → Outer Layer of Polar Plug and Inner Chitinous Layer → Inner Layer of Polar Plug). While Žďárská *et al.* (2001) [125] were the first to recognize two layers with differing electron densities in the polar plug of a *Huffmanela* species, Inatomi (1960) [51] reported that the outer (≅10%) of the plug of *Calodium hepaticum* is darker and more granular than the rest. (Incidentally, note that the figure captions and text in Inatomi (1960) [50] pertain to the figures in Inatomi (1960) [51] and *vice versa*). Fig. 4 of Grigonis and Solomon (1976) [43] (traced in Figure 11) confirmed Inatomi’s two-layered pattern for the plug. There have since been other TEM confirmations of the two-layered structure in polar plugs of *Trichuris*.

The eggs of *Syphacia obvelata* have a single operculum of a very different construction than those of trichinelloids. The margin of the operculum is defined by a quasi-circular groove in the Uterine Layer about 40 μm across near one end on the curved surface of the egg (pl. 3C of Wharton (1979) [114] that exposes a thin (about 50 nm wide) strip of the *Pellicula Ovi*. Beneath the narrow grove, there is a bead-like ridge in the Chitinous Layer about 2.5 μm wide and 1 μm thick. The ridge consists of electron-dense granular material quite similar in TEM texture to the Outer Layer of Polar Plug material seen in trichinelloid plugs, suggesting that this material may assist in loosening the plug, in preparation for eclosion in both groups.

In all TEM imagery of trichinelloid eggshells having laminated chitin that we have viewed, the alternating light and dark lamellated appearance of the Inner Chitinous Layer appears to end abruptly at its boundary with the material of the Inner Layer of Polar Plug. However, Wharton and Jenkins (1978) [120] indicated (in their fig. 8) that chitin fibers from the Inner Chitinous Layer in *T. suis* enter the matrix of the Inner Layer of Polar Plug. Indeed, a slight tweaking of the luminance curves in their fig. 6 reveals that the lamellae of the Inner Chitinous Layer actually continue across the plug. Panesar and Croll (1981) [84] also noted faint evidence of laminations in the plugs of *T. muris*. These observations suggest that the helical Bouligand arrangement of chitin deposition (see Section 4.3.4) continues across the plug, but with substantially less variation in opacity to an electron beam within lamella and also suggest that the plug material is formed from chitin the same way and at the same time the chitin wall is formed. We have seen no indication that the Inner Layer of Polar Plug of *H. huffmani* contains extensions of lamellae from the Inner Chitinous Layer. Monné and Hönig (1954) [72] indicated that the appearance of lamellar bands across the plug material of trichurids and capillariids may fade with age. Note that caution should be used when attempting to generalize from the latter paper, since the two study genera have very different chitin layers and the authors are not specific about which genus has which features.

**Figure 27:**
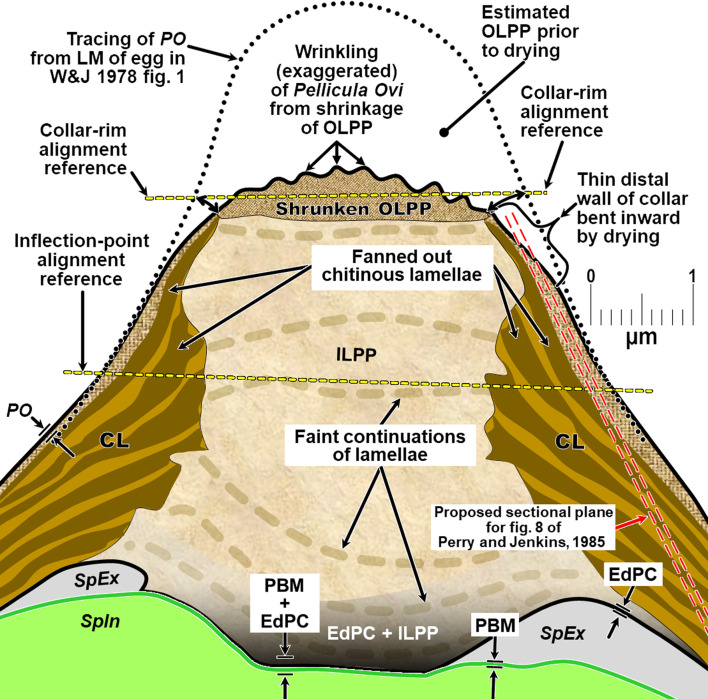
Interpretive tracing of a TEM sagittal of the polar plug of *Trichuris suis* from fig. 6 of Wharton and Jenkins (1978) [120], overlaid with an outline tracing (square dots) of a photomicrograph of an egg from their fig. 1, isometrically scaled to the same size. See Figure 2 for abbreviations and Section 4.7.3 for details on the double-dashed line.

In some trichinelloid genera, the Outer Chitinous Layer is thin equatorially but appears to be much thicker around the collar of the polar canal causing the appearance of a lucid, refractile ring around the outside of the canal wall (*i.e.*, fig. 6 of Wharton and Jenkins (1978) [120]). In an optical mid-sagittal section of the polar region, this expanded thickness of the Outer Chitinous Layer in the polar region is apparently responsible for the two ear-like bright spots sometimes seen in LM imagery (fig. 3 of Dill *et al.* (2016) [34], figs. 6 and 12 of Ruiz and Bullard (2013) [95]) and drawn as hyaline regions on either side of the neck of the polar canal. One can see this thickened Outer Chitinous Layer clearly in an oblique TEM section through the collar region of *Trichuris muris* in fig. 14 of Panesar and Croll (1981) [84]. The Outer Chitinous Layer seems to shrink substantially (along with the Outer Layer of Polar Plug) during the extreme drying protocols of TEM preparation, and the Outer Chitinous Layer in fig. 6 of Wharton and Jenkins (1978) [120] (traced in our Figure 27) is probably much thicker in fresh mounts than is shown in their fig. 6.

If one were to follow *Trichuris* lamellae from the equatorial region to the polar region (Figure 27), the inner half of the lamellae continue on an ellipsoid trajectory as they close in toward the axis of the canal while the outer half abruptly depart from the ellipsoidal trajectory at the collar and flare outward as they near the canal, with the outer-most lamella becoming almost parallel to the long axis and forming a thin-walled collar extending the polar canal distally.

We observed SEM images of *H. huffmani* eggs that had lost all the Uterine Layer except for a remnant forming a ragged corona around only one canal rim, the other rim most often having no such corona (Figure 6C). Since trichinelloid larvae have been observed being successfully ejected from only one of the two poles [84], perhaps the end that has the corona of Uterine Layer (or the one without) is associated with the eclosion of the larva. Unfortunately, none of our TEMs happened to include such coronas, but a close examination of the sagittal section in fig. 9 of Žďárská *et al.* (2001) [125] shows an intimate connection between the Outer Layer of Polar Plug and the Outer Uterine Membrane on both sides of the plug right where the fibers of the Inner Chitinous Layer connect to the Outer Layer of Polar Plug.

#### 4.6.4 The Electron-dense Parietal Coating (formerly Lipid-rich Layer) of the polar region

The Electron-dense Parietal Coating, which coats the parietal surface of the Rigid Eggshell Wall, continues across the proximal margin of the Inner Layer of Polar Plug; however, the material of the Electron-dense Parietal Coating seems to intermingle with the material of the Inner Layer of Polar Plug much more extensively than where it is applied to the rest of the Chitinous Layer. This extensive intermingling of the Electron-dense Parietal Coating with the Inner Layer of Polar Plug, in most TEMs of polar plugs, forms a diffuse centrifugal gradient from darker to lighter electron density in contrast to the equatorial portion of the Rigid Eggshell Wall, where the intermingling of the Electron-dense Parietal Coating and Inner Chitinous Layer is usually much less extensive. This contrast seems to be universal across the trichinelloids and may indicate that the material of the Inner Layer of Polar Plug is softer and/or more porous than the adjacent Inner Chitinous Layer that forms the collar.

#### 4.6.5 The Unoccupied Parietal Space of the polar region

Most trichinelloid eggs are shaped by the Rigid Eggshell Wall into something like a truncated prolate spheroid. The embryo/larva occupies most of the central 80% of the parietal cavity, thus forcing any displaceable fluid in the cavity into the spaces toward the narrower poles, where it accumulates just internal to each of the polar plugs. Both the *Spatio Externum* and *Spatio Internum* are well represented in the polar region of the trichinelloid Unoccupied Parietal Space between the Inner Layer of Polar Plug and the embryo/larva, and the malleability of the Permeability Barrier Membrane is best observed in this open space (see fig. 4 of Appleton and White (1989) [6]). Sometimes the Permeability Barrier Membrane appears wispy and diffuse in TEMs of this region, but that might be due to solvents used in TEM processing, or perhaps due to the knife intercepting a fold in the membrane.

Panesar and Croll (1981) [84] estimated that the total volume inside the Rigid Eggshell Wall of *T. muris* (the Unoccupied Parietal Space) was about 15.5 × 10^3^ μm^3^. The amount of volume occupied by the embryo decreases slightly as the larva develops and fluid is added to the *Spatio Internum* until the fully developed larva occupies about 70%, leaving about 30% of the total volume for wiggle room. Just before they were ready for eclosion, the larvae swelled, apparently by swallowing fluid, to displace almost the entire space just before being ejected [84].

### 4.7 Artifactual “features” associated with the polar plug

A consistent problem we have encountered in reviewing prior studies of trichinelloid eggshells and the polar regions of *Huffmanela* eggs is that authors rarely address the fact that TEM and SEM images of the plug region are not consistent with each other nor with LM imagery of wholemount eggs from the same collection in the same paper. While SEM and TEM studies are helpful for determining surface fine structure and compositional boundaries, respectfully, the polar region is dramatically affected by the requisite drying processes, and the egg proportions are often so distorted as to render the egg metrics almost unrecognizable. Thus, some artifactual egg “features” described for the polar regions of trichinelloid eggs are based on unexpected technique-induced artifacts associated with extreme drying of SEM and TEM protocols and interpreted from a confused understanding of normal eggshell anatomy and development. In the following sections, we address some of these problems, their hypothetical causes, and recommended precautions and solutions.

#### 4.7.1 “Plugs extended” (or not)

LM imagery of fresh or formalin-fixed trichinelloid eggs show a hyaline and refractile plug region that may extend barely or well beyond the distal rim of the polar canal. Sometimes an inappropriate degree of taxonomic attention is given to the degree of plug extension; or egg-length measurements might include the bulging tips of the polar plugs as seen in LM whole mounts. While this seems to be reasonable for intact eggs in orthogonal view, there are two caveats to consider. Firstly, the uterine layer of *Huffmanela*, if present, should not be included in diagnostic metrics because it is quite labile and subject to substantial alteration by age [125], handling, and technique. Secondly, the farther the plug of a trichinelloid species normally extends beyond the collar, the higher will be the coefficient of variation for total length, reducing diagnostic utility. For descriptive studies, we recommend reporting the length of the Rigid Eggshell Wall from extremes of the chitinous rims of the polar canals as seen in Figure 26, which can usually be accurately resolved in clear LM imagery and will have a much lower coefficient of variation than total-length metrics that include the plug extensions. Thirdly, some authors studying whole mounts under LM have attempted to report the lengths of polar plugs by measuring from the distal tip to what appears to be proximal margins of the polar canal. This is invariably misleading, since the material of both the Outer Layer of Polar Plug and Inner Layer of Polar Plug are usually completely transparent to the visible spectrum, and while the distal end extending beyond the eggshell is usually obvious, its proximal extension centripetally can rarely be accurately discerned by LM. Additionally, the *Spatio Externum* of some eggs seems to be gel-like and may be confused with the base of the plug (see Bullard *et al.* (2012) [24]; figs. 1D, G for examples). Fourthly, the dark bands commonly drawn across the canals based on LM studies of wholemounts (Figure 26) are not really structures or layers, but simply represent the lateral, distal, and proximal margins of the canal itself; perhaps worth reporting as a visual character but having nothing to do with length of the polar plug. Indeed, the proximal (centripetal) margin of the plug sometimes bulges centripetally beyond this band as in fig. 6 of Wharton and Jenkins (1978) [120] and our Figure 27, or ends somewhere within the canal (Figures 24 and 25). Plug boundaries might be discernible by brightfield LM of thin paraffin sections or by viewing wholemounts by DIC, but rarely by brightfield LM of wholemounts.

#### 4.7.2 Canal details

Another product of artistic license that often shows up in drawings of the polar region is a few divergent longitudinal lines near the inner opening of the canal as in fig. 1 of Moravec and Campbell (1991) [76]. Although we have seen such lines in LM wholemounts, they appear not to be representative of any structure in the canal. However, lines sometimes drawn *across* the canal, as in fig. 1 of Beer (1973) [11], especially in *Trichuris* spp. where the lamellae are thick and bold in the polar region, represent the helical spiral of fibers physically projecting into the canal as can be seen in freeze-fractured views of a *T. trichiura* canal from which the plug has been lost (fig. 6 of Meng *et al.* (1986) [70]) and can also be seen as “ringed thickenings” in LM, as in fig. 6 of *Trichuris vulpis* in Traversa *et al.* (2011) [106].

#### 4.7.3 Electron microscopy and the polar region

Several problems arise when attempting to correlate LM observations of trichinelloid eggs with imagery from SEM and TEM. The boundaries between the two plug sublayers are sometimes detectable in LM (our Figure 3B, and figs. 9, 10, 12, 13 of Ruiz *et al.* (2013) [96]), but measurements and drawings should not include such boundaries without TEM validation.

**Figure 28:**
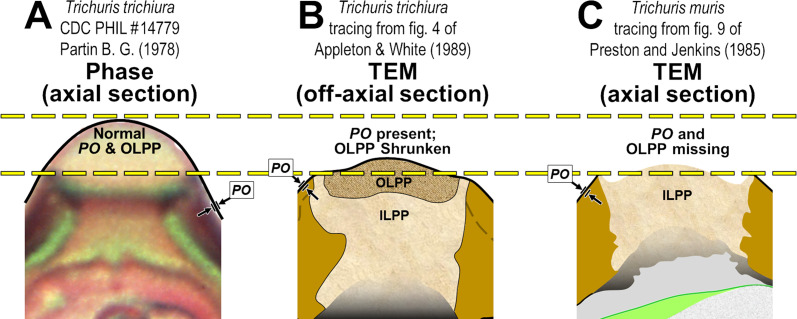
Effects of various techniques on polar plug appearance: (A) LM [85] showing normal plug; (B) tracing of TEM showing plug shrunken from dehydration (see also Figure 21); (C) tracing of TEM showing how the Outer Layer of Polar Plug and *Pellicula Ovi* have separated and been lost in processing, exposing the Inner Layer of Polar Plug (see also Figure 29).

The hydration states of the two sublayers apparently differ substantially in viable eggs, and these differences may be responsible for some of the puzzling morphometric changes caused by drying protocols commonly used SEM and TEM. Notice how in Figure 28A the bulging plug seems to be filled with clear fluid under pressure, thus stretching the *Pellicula Ovi* tight, but in Figure 28B it has shrunken due to drying protocols in preparation for TEM.

We tested for unbound water in the plug material of viable *H. huffmani* eggs by submerging some in a hyperosmotic saline solution while others from the same batch soaked in deionized water. We did not see a significant effect on mean total lengths of the two batches after 24 h of exposure, suggesting that there is probably not enough unbound water in the plug material to account for the shrinkage caused by the drying protocols of electron microscopy. We have also seen fresh eggs with dislodged plug caps (Outer Layer of Polar Plug) that had maintained the original shape and refractile properties (Figure 5), all this suggesting that the material of the Outer Layer of Polar Plug must be a semisolid, but probably does not contain much unbound water. On the other hand, there must be an abundance of bound water in the plug material because the extreme drying protocols required for EM preparation result in substantial reductions in plug extension and also in the diameter of the distal polar-canal opening (Figure 27).

**Figure 29:**
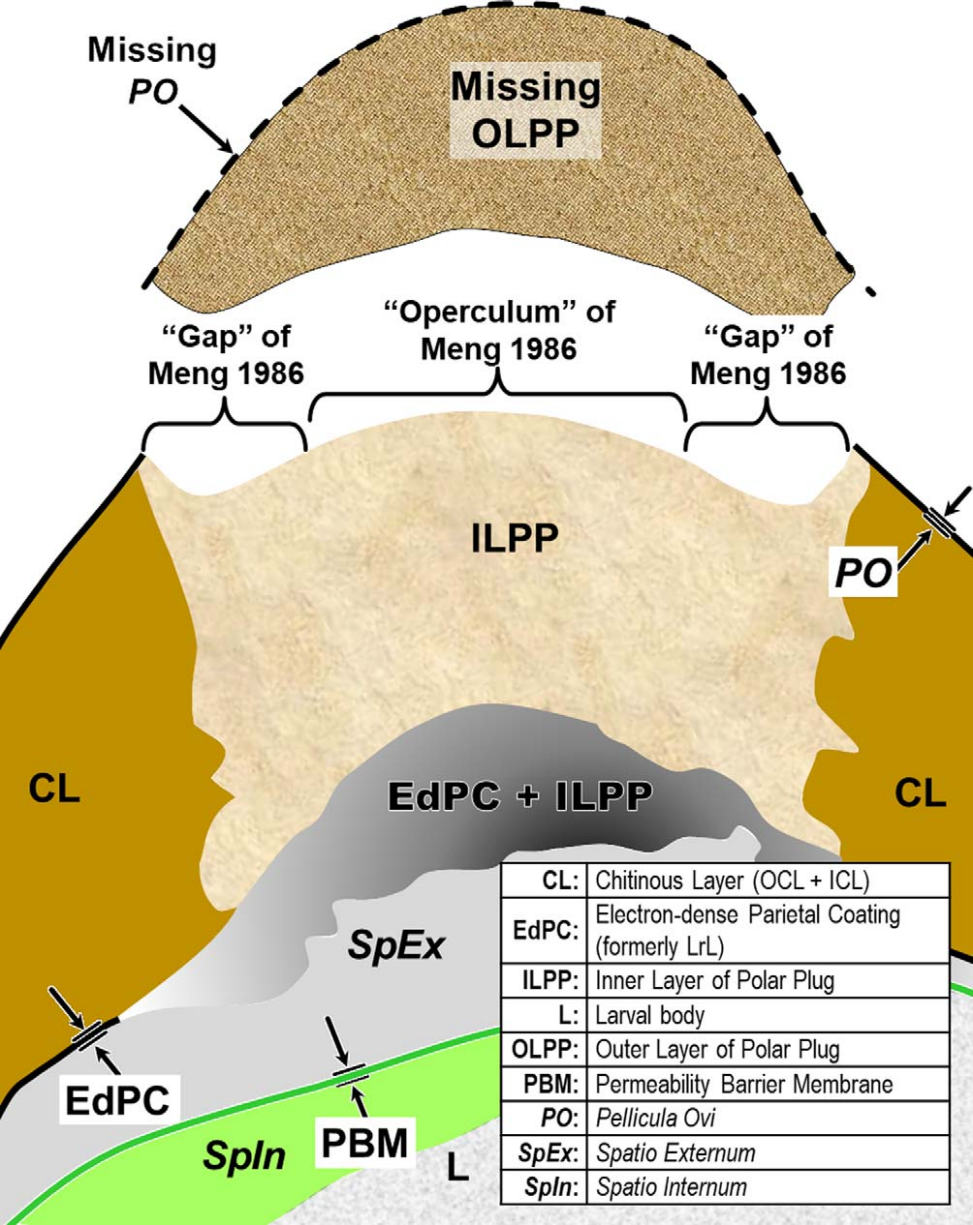
Interpretive tracing of a TEM of the Polar Plug region of *Trichuris muris* (adapted from fig. 9 of Preston and Jenkins (1985) [89]) showing the estimated dimensions of the missing Outer Layer of Polar Plug and *Pellicula Ovi*, both scaled to match photomicrographs of wholemounts in their fig. 1. Curly brackets represent terms introduced by Meng *et al.* (1986) [70] to describe what was seen in SEMs *T. trichiura*, where the Outer Layer of Polar Plug had apparently not been lost but had shrunken away from the collar wall leaving it surrounded by a moat-like trough.

In some SEMs of *Huffmanela*, the plug material had withdrawn back into the canal without breaking the *Pellicula Ovi*, forming a smooth parabolic depression as in fig. 16 of Ruiz and Bullard (2013) [95]. Other times the drying procedures shrank the Outer Layer of Polar Plug back centripetally so much that the plug cap apparently tore away from the Inner Layer of Polar Plug and fell out during handling, taking the attached *Pellicula Ovi* with it. These modifications happen surprisingly often (fig. 3 of Meng *et al.* (1986) [70]; fig. 11 of Panesar and Croll (1981) [84]; fig. 9 of Preston and Jenkins (1985) [89], the latter traced and re-interpreted in our Figures 28C and 29) and has led to some confusing commentary from puzzled authors. Indeed, Preston and Jenkins (1985) [89] said of the exposed ragged surface of the Inner Layer of Polar Plug that it was covered by *Pellicula Ovi* (when it clearly was not) and referred to the image as representing the “fine structure of the fully formed polar plug” despite their fig. 1 showing complete eggs with protruding plugs. In still other cases, the Outer Layer of Polar Plug may shrink back into the canal so far that it causes the *Pellicula Ovi* covering the tip of the Outer Layer of Polar Plug to tear away from the rest of the membrane leaving the wrinkled remnant of the *Pellicula Ovi* connected to the rim of the polar canal, providing a spectacular view of a dome-shaped pleated membrane with a hole in the center (fig. 3 of Appleton and White (1989) [6]) like a stadium roof with its iris open.

Notice in fig. 3 of Appleton and White (1989) [6] how the ring of *Pellicula Ovi* surrounding the crater seems to be sutured securely to the underlying rim at its base. We have seen evidence of this attachment ring in other images, and it is apparently to preserve the shorter radius of curvature of the protruding plug when it is hydrated and turgid, as in fig. 6 of Panesar and Croll (1981) [84].

Incidentally, in fig. 8 of Preston and Jenkins (1985) [89] (a *T. muris* egg), the material labeled “PL” and described as being a section through the plug material, cannot possibly be a section through the plug material because it is uniform in composition and bordered on three sides by the uterine contents and by Inner Chitinous Layer on the fourth. See the red double dashed line in our Figure 27 (*T. suis*) for an estimated plane of a section that could reproduce fig. 8 of Preston and Jenkins (1985) [89]. The material labeled “PL” in their fig. 8 is a tangential/oblique section through the Outer Chitinous Layer of the outer wall of the collar and also intercepts the lamellated Inner Chitinous Layer area that surrounds the base of the collar as indicated by the strongly arched lamellae. The “fine interwoven network of chitin-protein microfibrils” surrounding the “PL” label and attributed to the plug material consists of randomly oriented fibers of the Outer Chitinous Layer of the collar wall as discussed in Section 4.3.4.

Perhaps the best example of the most common type of inconsistencies that can result from TEM studies of trichinelloid eggs is demonstrated in the Wharton and Jenkins (1978) [120] study of *Trichuris suis* eggs. Fortunately, the authors included clear photomicrographs of whole mounts of several eggs in their fig. 1. We isometrically enlarged the image of one of those eggs in their photomicrograph and carefully traced the outline of one polar region. We then isometrically scaled that outline to the same scale as the sagittal TEM in their fig. 6. Proper scale was determined by aligning the distal rims of the collars in the wholemount with where the collar would probably have been without shrinkage (see double-arrowed arcs in Figure 27) and then by isometrically adjusting the size of the outline such that the points where the curvature was most concave in the two outlines are superimposed at the base of the collar (Figure 27). The discrepancy between the black solid line (PO) and dashed outlines in Figure 27 is an estimate of the artifactual shrinkage caused by TEM drying protocols. Clearly, polar plug metrics should not be based on TEM or SEM studies that use severe drying protocols. On the other hand, high-pressure freezing TEM might revolutionize the study of trichinelloid polar plugs.

Apparently, the Outer Layer of Polar Plug is solely responsible for the extension of the polar plug beyond the rim of the collar, producing the refractile and hyaline polar “cap” of wholemount eggs as seen in Figures 3C and 3D. Note also in fig. 6 of Wharton and Jenkins (1978) [120] (see our Figure 27) how the extreme distal part of the collar wall in *T. suis* is made of thin chitin (composed mostly of Outer Chitinous Layer and the outermost lamella of the Inner Chitinous Layer) and is normally parallel with the axis of the collar, but has caved in towards the axis in (Figure 27) because the plug material shrank inwardly during preparatory drying. This has caused the *Pellicula Ovi* over the cap to become wrinkled as can be seen in the micro-crenulated margin of the associated *Pellicula Ovi* in the original TEM where it passes over the plug compared to the smoother *Pellicula Ovi* covering the base of the collar. As far as we are aware, this disproportionate shrinkage of the trichinelloid plug (relative to the rest of the Rigid Eggshell Wall) was first correctly alluded to by Appleton and White (1989) [6] but has since been recognized by others. What has apparently not been recognized is that most of the shrinkage, by far, is in the Outer Layer of Polar Plug, and thus the dense, granular texture of the Outer Layer of Polar Plug under TEM may not appear so coarse prior to the extreme dehydration. It would be difficult to explain how the material that is so uniformly transparent under LM is actually as granular *in vivo* as it appears to be under TEM. High-pressure-freezing TEM could help resolve this issue.

Another conclusion that can be drawn from these observations is that the *Pellicula Ovi*, at least in trichinelloids, appears to have considerable tensile strength, but is also quite elastic. Consider the egg shown in Figure 26b with its record long polar extension; when the *Pellicula Ovi* was first formed, its shape was that of a prolate spheroid. When the plug material began to swell out axially, the *Pellicula Ovi* had to stretch balloon-like (apparently without adding material) to a surface area probably 10× greater than when it was initially formed around the zygote, and still maintain its characteristic shape. When a trichurid egg approaches hatching and the egg begins to swell noticeably, the *Pellicula Ovi* over the Outer Layer of Polar Plug stretches out like a balloon but resists the mounting pressure until the Permeability Barrier Membrane is punctured by the larval stylet just before eclosion (see progression in figs. 3–6 of Panesar and Croll (1981) [84]).

The first thing that grabs one’s attention when viewing a sagittal TEM of the polar region of an embryonated *Trichuris* egg is the flaring of the lamellae at the poles. While we have not seen a study documenting the sequence that results in this flaring, the final result suggests that the plug has grown by taking on water *after* all the lamellae in the adjacent wall were completely formed. This is especially suspect in fig. 6 of the Wharton and Jenkins (1978) [120] study of *T. suis* (see also our interpretive tracing in Figure 27). We have estimated that the Inner Layer of Polar Plug of *T. suis* is at least 5× times as thick axially as the radial thickness of the equatorial Inner Chitinous Layer, and the Outer Layer of Polar Plug (before dehydration) is about 22× as thick axially as the equatorial Outer Chitinous Layer is radially. Furthermore, since the shell is assembled in centripetal sequence [83], and the outermost lamellae of Figure 27 are flared outwardly while the innermost lamellae (including their traces in the Inner Layer of Polar Plug) are flared inwardly, it follows that the expansion of the plug probably does not start until after all the lamellae of the Inner Chitinous Layer have been assembled and all the material of the plug has been deposited. Indeed, the Inner Chitinous Layer near the canal often appears to be torn apart by the longitudinal expansion of the plug material as is suggested by a sagittal freeze fracture of the canal of a *T. trichiura* egg in fig. 6 of Meng *et al.* (1986) [70] and some TEMs we have seen.

**Figure 30:**
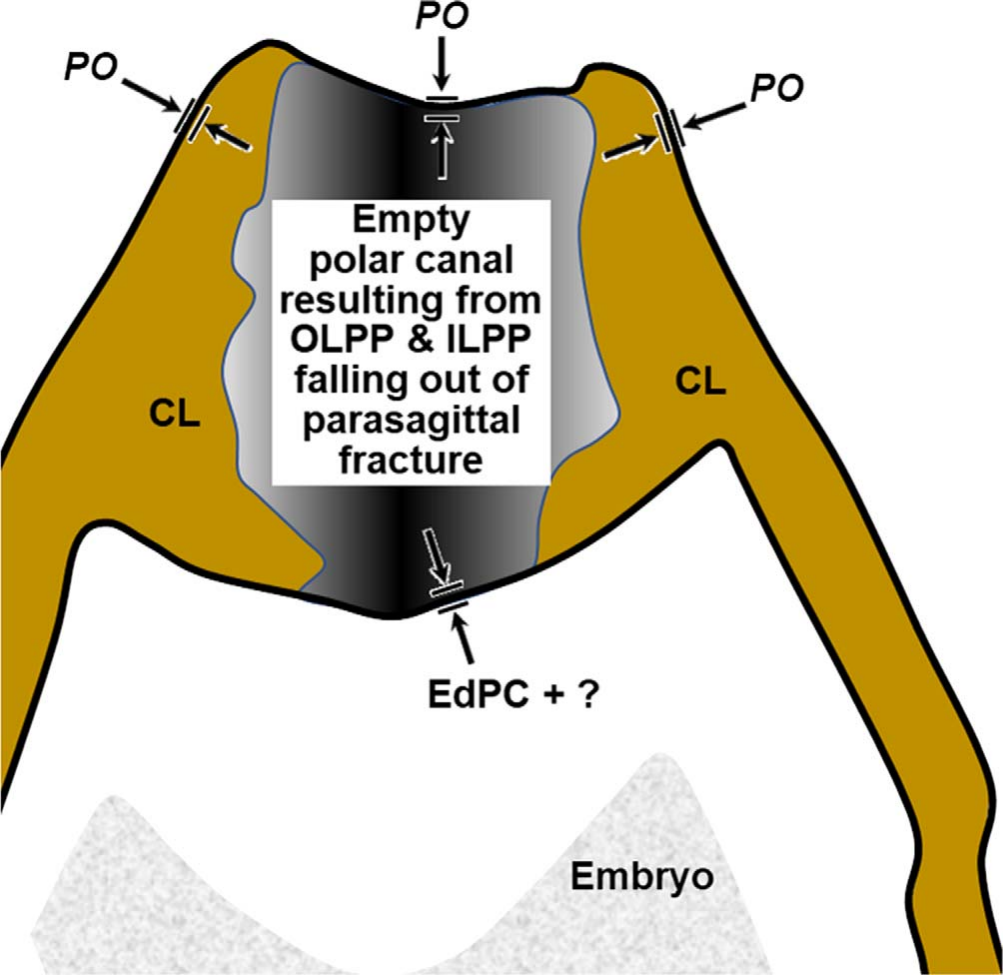
Interpretive tracing from a freeze-fracture image from fig. 6 of Meng *et al.* (1986) [70] showing a more accurate interpretation of the roof (*Pellicula Ovi*) and floor (Electron-dense Parietal Coating) of the evacuated polar canal of *T. trichiura*.

Some other illuminating applications of the new hexalaminar framework to the trichinelloid polar region can be drawn from freeze fractures and broken specimens. Fig. 5 of Appleton and White (1989) [6] is an SEM showing where a longitudinal “banana peel” section of the Rigid Eggshell Wall fell out from a fractured specimen of an egg of *Trichuris trichiura*. Several structures that are visible here can clarify some misinterpretations. At the end of that egg, the Outer Layer of Polar Plug has shrunken back to form a recessed dome surrounded by a moat-like “gap” *sensu* fig. 3 of Meng *et al.* (1986) [70]. Beneath the Outer Layer of Polar Plug (arrow) is a cavity formed in the polar canal previously occupied by the Inner Layer of Polar Plug. In this case, the floor of the chamber, which was formed when the Electron-dense Parietal Coating was laid down on the bottom of the Inner Layer of Polar Plug, is missing. Not so with a similar situation provided by fig. 6 of Meng *et al.* (1986) [70]. This image is a freeze fracture exposing the completely empty polar canal of a *T. trichiura* egg with its roof and floor intact, as shown by the interpretive tracing in our Figure 30. Note that the Electron-dense Parietal Coating could not be expected to form an integral floor like this unless there are fibrous molecules (CPG backbones?) imbedded in the lipid. Though it is not obvious from the freeze-etch imagery, the *Pellicula Ovi* forms the roof of the plug chamber, as can be seen in the *T. trichiura* eggs in fig. 4 of Appleton and White (1989) [6] (see tracing in our Figure 21) and also in the *T. suis* eggs in fig. 6 of Wharton and Jenkins (1978) [120] (see tracing in our Figure 27). Electrical charge artifacts apparently caused by chitin/protein fibrils projecting into the empty polar canal of freeze-fractured specimens are sometimes interpreted as a membranous lining as in fig. 6 of Meng *et al.* (1986) [70], but clearly from TEMs of other authors, there are no such linings.

We can find no visual account of the sequence of events in the trichinelloid egg from the early determination of polarity through the extension of the polar plug beyond the canal rim. The Preston and Jenkins (1985) [89] study shows what appears to be an expanded plug in their fig. 6 prior to occurrence of lamellae in the chitin, but there appear to be several other interpretive errors in this paper and it’s difficult to see what is being portrayed in the image. Robertson *et al.* (2022) [92] provided some imagery in their fig. 4 of the internal ultrastructure of *T. muris* using serial block face-scanning electron microscopy (SBF-SEM) with microwave-assisted sample processing and was able to reveal the 3-dimensional architecture of the polar-plug region. This seems to be a very promising technology for solving some of the pressing questions regarding nematode eggshells. Since the record-long extended plug of *Crocodylocapillaria longiovata* is formed entirely *in utero* [79] and the species is relatively abundant in cultured crocs, it seems like a natural subject for a study of polar plug development.

### 4.8 Some peculiarities of *Huffmanela* as a trichinelloid

#### 4.8.1 The Uterine Layer

*Huffmanela huffmani* appears to be the first clade in the superfamily Trichinelloidea declared to have a Uterine Layer. Other clades of *Huffmanela* have been described with “outer envelopes” that now appear to be Uterine Layers. Other non-trichinelloid genera with uterine layers have regularly arranged spaces in the Inner Uterine Layer connected to pores in the Outer Uterine Membrane, while the Inner Uterine Layer of *H. huffmani* has no such spaces or pores. Some other *Huffmanela* species appear to have Superficial Projections on the Outer Uterine Membrane like those of *H. huffmani*, while others appear to be smooth, and still others ornate in various other ways. Thus far among *Huffmanela* clades, only *H. japonica* [78] (see fig. 3C–E) has been reported to have sculpturing of the Uterine Layer similar to what is commonly found on eggshells of other orders having a Uterine Layer.

Eggs of some species of *Huffmanela* are described as having no outer “envelope” (referring to what we now know is the Uterine Layer), but we have seen collections of *H. huffmani* eggs in which most eggs in the collection have lost the Uterine Layer. Since all known species of *Huffmanela* are histozoic and the Uterine Layer is in intimate contact with the host tissue [125], the process of mechanically separating the eggs from the tissue during necropsy can easily tear the egg away from its Uterine Layer, leaving the Uterine Layer behind undetected in the tissue, especially when eggs have Superficial Projections on the Uterine Layer. The Uterine Layer of *Huffmanela* species appear to be much more delicate (Figure 5) than the Uterine Layers of other orders that have the layer; so, in order to confidently claim that the eggs of a *Huffmanela* clade lack a Uterine Layer, larvated eggs should be examined in tissue (ideally, sectioned). One caution is that in older infections, a host-reaction cyst around individual eggs can look like a ragged Uterine Layer.

#### 4.8.2 Rigid Eggshell Wall

Other than for the presence of a Uterine Layer, the equatorial portion of the *H. huffmani* eggshell appears representative of what we see in other trichinelloids, although the Inner Chitinous Layer of the only other *Huffmanela* clade to be studied via TEM [7] appears not to be lamellated.

In some cases in which eggs of *Huffmanela* spp. are declared to have no outer envelope, there appears to be an outer membrane of some sort surrounding the eggs in published imagery that was missed by the authors. The egg images of *Huffmanela* cf. *carcharhini* ([62]) [74] from sandbar shark near Hawai’i are particularly intriguing. Bullard *et al.* (2012) [24] reported that the eggs of the species had “no obvious shell envelope” (referring to the eggs having no Uterine Layer). However, a close look at the SEM of an egg in their fig. 3B clearly shows an extremely thin membrane with a patch of it torn away in the upper right quadrant of the image, exposing what appears to be a spongy matrix beneath, and there appear to be other abrasions of this envelope in the lower half. The paradox this image presents is that we have never seen the Outer Uterine Membrane of any species torn away from the Inner Uterine Layer, but we have seen the *Pellicula Ovi* covering an open mesh-like matrix of anastomosing chitinous strands in the Outer Chitinous Layer of capillariid eggs that has worn away from the chitin in places causing similar SEM images (see Section 4.3.2). Also, the membrane in question in Bullard *et al.* (2012) [24] is extremely thin, much thinner than we have ever seen for any Uterine Layer and, therefore, is more likely the *Pellicula Ovi* of those eggs that has torn away from the Outer Chitinous Layer. Please compare the images in figs. 1D, G and 2B of Bullard *et al.* (2012) [24] *vs*. the longitudinal and cross sections (respectively) of eggs of *Calodium hepaticum* in Anonymous (2019) [5]. Note how the outer layer of the eggshell in both species ends at the rim of the polar canal and does not cover the plug. This means that the outer layer of the eggs of both species is probably the modified Outer Chitinous Layer, and not a thick uterine layer. However, the Outer Chitinous Layer of *C. hepaticum* is not quasi-radial fibers as in the shark forms of *Huffmanela* (see also fig. 6B of Attia *et al.* (2021) [7]), but a somewhat consistently repeating geometric pattern of “pillars” and “beams” (fig. 2 of Grigonis and Solomon (1976) [43]). Even though the eggshells of *Huffmanela* clades from sharks are thus categorically different from those from teleost hosts, recent *18S* sequence analysis of a few *Huffmanela* clades [23] suggests that the freshwater clades of *Huffmanela* from teleost hosts are much different from at least some of those from selachian hosts. More sequences are required from marine teleosts for a decision, and this work is underway.

#### 4.8.3 Unoccupied Parietal Space

What appears to be the *Spatio Externum* and *Spatio Internum* separated by a flexible Permeability Barrier Membrane is visible in almost all our TEM imagery of embryonated *H. huffmani* eggs. However, we have not seen any evidence of fibrous inclusions in the *Spatio Externum* of *H. huffmani* like those present in the same space in some *Trichuris* TEMs (see fig. 2 of Wharton and Jenkins (1978) [120], traced in our Figure 22).

#### 4.8.4 Polar region

In *Trichuris*, the Inner Chitinous Layer flares dramatically as it approaches the polar canal (see example in Figure 27). In contrast to *Trichuris*, the lamellae in the Inner Chitinous Layer of *H. huffmani* do not thicken or flare as they approach the polar canal (Figures 24 and 25). Also, the Inner Layer of Polar Plug of *H. huffmani* is consistently lenticular in shape with a conical proximal margin, appears to be attached only to the outer 1/3 of the lamellae of the Inner Chitinous Layer, and does not fill the remainder of the canal as it does in *Trichuris*. Indeed, the lamellated layer of the *H. huffmani* Inner Chitinous Layer ends abruptly at the canal, with the inner 2/3 of the canal appearing to be filled with gel-like material of the *Spatio Externum* instead of the Inner Layer of Polar Plug. The innermost few lamellae of the equatorial Inner Chitinous Layer terminate before they reach the canal, causing the Inner Chitinous Layer to taper in thickness as it approaches the canal. This tapering pattern seems also to be characteristic of *Calodium hepaticum* (Figure 11). Boundaries between the Outer Layer of Polar Plug, Inner Layer of Polar Plug, *Spatio Externum*, Permeability Barrier Membrane, and *Spatio Internum* in the polar region of *Huffmanela* spp. are sometimes fortuitously discernible using brightfield LM (Figure 9). However, it is important to point out that we know where to look for boundaries only because TEM showed us where they are. Any nematodologist tracing the LM image in Figure 9 without consulting TEM sections as a guide would probably put some of the boundaries elsewhere.

In some of our TEMs of *H. huffmani* polar regions, and also fig. 9 of Žďárská *et al.* (2001) [125], random fibers from the Outer Chitinous Layer reach into the Outer Layer of Polar Plug and appear to anchor it in place (Figure 24). Note also in Figure 24 how the apparent expansion of the Outer Layer of Polar Plug subsequent to formation of the Rigid Eggshell Wall has torn the Outer Layer of Polar Plug away from the Inner Layer of Polar Plug, creating gaps between the layers.

We are perplexed by the LM imagery of the polar regions of *Huffmanela* eggs from sharks, especially fig. 1D of Bullard *et al.* (2012) [24], fig. 3 of Dill *et al.* (2016) [34], and fig. 12 of Ruiz and Bullard (2013) [95] which in general shape and appearance seem inconsistent with our TEMs of *H. huffmani* polar regions and clear LM imagery of eggs of other *Huffmanela* populations, and also differ from the TEMs we have studied from Capillariidae and Trichuridae. Another intriguing mystery arises from the *Huffmanela* sp. of Attia *et al.* (2021) [7] (also from sharks). Their figs. 5A, 6C, D show a thin layer about 100 nm thick of somewhat electron-lucid material lining the parietal surface of the Inner Chitinous Layer (labeled “lp”) that is completely lacking in their other images (6A & 6B). We do not know what this layer represents; however, it has an appearance and texture similar to that of the CPG Layer in the *C. elegans* imagery of Olson *et al.* (2012) [83]. Could it be that there is a transient phase in the formation of nematode eggshells wherein the CPGs form a discernible layer that disappears shortly thereafter? Could this perhaps explain why the distinct CPG Layer clearly present in the *C. elegans* eggshell imagery of Olson *et al.* (2012) [83] is not discernible in most other TEMs of the *C. elegans* eggshell? These questions and others expressed herein regarding the CPG Layer must be addressed before nematodologists outside the *C. elegans* Research Community will seriously consider the CPG Layer as a valid member of any generally applicable anatomical model of the nematode eggshell.

Unfortunately, none of the TEM sections of the *Huffmanela* sp. in Attia *et al.* (2021) [7] intercepted the polar canal, so we do not yet know what to expect for the polar regions of species from sharks. One curious issue we would like to see resolved is the apparent eosinophilic ring surrounding the rim of the polar canal and appearing to be connected to the *Pellicula Ovi* in the shark form displayed in fig. 3 of Dill *et al.* (2016) [34].

Both the *Spatio Externum* and *Spatio Internum* are voluminous in the polar regions of most trichinelloid imagery, but the two layers differ in electron density (Figure 24) and texture under LM (Figure 9). When watching a late larva moving around in an egg of *H. huffmani*, the larva seems less able to deform the *Spatio Externum* than the *Spatio Internum*, suggesting that the *Spatio Externum* is composed of material that congeals into a viscous colloid or soft gel. The accumulation of *Spatio Externum* in the polar regions of the eggs looks like part of a large Polar Plug in most *Huffmanela* images from both TEM (Figure 24) and LM (Figure 9). This illusion is accentuated by the relatively shallow plug of *H. huffmani*, with a conical proximal margin extending only about half way down the canal, rather than bulging into the Unoccupied Parietal Space as it often does in trichurids (Figures 21, 27, and 30).

### 4.9 Some challenges to the universality of the hexalaminar eggshell framework

#### 4.9.1 The hexalaminar anatomical model

All six major eggshell layers, as defined in our adaptation of Olson’s (2012) [83] *C. elegans* hexalaminar anatomical model, are readily discernible in published TEMs of most nematode groups. Indeed, while *Caenorhabditis* and *Trichuris* are near opposite ends of the recent nematode phylogenies we have seen [33, 46, 59, 109], both genera exhibit all six layers of the hexalaminar anatomical model in TEMs of mitotic eggs. However, one group seems to be somewhat exceptional – the Tylenchida, eggshell ultrastructure of which has been studied rather extensively. While we have found representations of all six layers in some published TEMs of mitotic tylenchid eggshells we have examined, some of the layers have exceptional features in some images. Specifically, one or more bilaminar or multilaminar membranes often appear packed together just internal to the Rigid Eggshell Wall of tylenchid eggs and sometimes several membranes are loosely associated with the *Spatio Externum* and what would otherwise be labeled as the Electron-dense Parietal Coating (fig. 8 of Bird (1976) [15], figs. 2A, B of Bird and McClure (1976) [17], fig. 2 of Burgwyn *et al.* (2003) [25], fig. 1C of Perry and Trett (1986) [86], figs. 2, 5 of Perry *et al.* (1982) [87]). If the eggs of other more obscure nematode groups are studied with TEM for compliance with the new hexalaminar framework, other peculiar adaptations may be discovered.

At this point, one thing seems abundantly clear: the classical trilaminar anatomical model must be replaced with the anatomical model of the new hexalaminar framework. However, the most serious challenges to the universality of the hexalaminar framework that must be addressed come not from the anatomical relationships or presenting features of those six layers, but from competing claims regarding the compositional and functional aspects of those layers, especially Layer #3.

#### 4.9.2 The composition of Layer #3

There seems to be substantial corroborative support for most of the hexalaminar anatomical model among published TEM images we have seen from diverse nematode taxa, including that Layer #3a is appressed onto the parietal surface of the Inner Chitinous Layer and is produced from cortical granule cargos from the oocyte. However, the composition of Layer #3 remains a point of serious contention. While there is strong and varied prior support for Layer #3 being rich in lipids, Olson *et al.* (2012) [83] and Stein and Golden (2018) [103] implied that Layer #3 instead contains CPGs (see direct quote this section) and therefore Layer #5 should be considered the Lipid-rich Layer of the nematode eggshell instead of Layer #3. Unfortunately, that conclusion appears to be based on an incomplete interpretation of the POD-2 experiment on p. 739 of Olson *et al.* (2012) [83]. It now appears that there are two layers at position #3 in *C. elegans*; a thin and faintly stained lipid layer (Layer #3a) and an obvious CPG layer (Layer #3b), at least in some imagery (see TEM references in Section 4.4.2). The failure of the POD-2 treatment to interfere with the formation of Layer #3b does not mean that Layer #3a does not contain lipids, and the Olson team did not realize that when they developed their anatomical model of the *C. elegans* eggshell, they had *added* a CPG sublayer to an *existing* Lipid-rich Layer at position #3 (a.k.a. “inner eggshell layer”), but had not *replaced* the Lipid-rich Layer at position #3 with the CPG Layer. Additionally, (1) many clear TEMs of the *C. elegans* eggshell in the literature do not show an obvious CPG Layer like the obvious layer in the Olson [83] paper, and (2) we have yet to see evidence that any nematode eggs other than *C. elegans* have a CPG Layer at position #3.

Olson *et al.* (2012) [83] and Stein and Golden (2018) [103] explain that the reason previous investigators had concluded that Layer #3 contained lipids was because of lipid extraction experiments performed prior to 1950 [29]. However, lipid extraction is only a small part of the story behind the earlier Electron-dense Parietal Coating (Lipid-rich Layer #3) theories, and much more persuasive evidence is given in later reports referring to histochemical indications that Layer #3a (the Electron-dense Parietal Coating) is, indeed, “rich” in lipids. Most of the substantial lipid claims for Layer #3a were not made from an extraction or hypothetical gene/enzyme/interference conjecture, but from the staining reaction of Sudan Black B (SBB), a stain that has been trusted by histochemists for decades as an indicator that lipids are present in a tissue. Table 1 lists four papers providing histochemical evidence that Layer #3 contains lipids in four species from three different nematode orders. We searched recent literature for any indications that SBB might have been found to have an affinity for any major biochemical class besides lipids, including CPGs, but could find none. Not all advocates of SBB as a reliable indicator of lipids claim that it will indicate all lipids (there are documented situations where SBB has missed certain chemicals classified as lipids, *i.e.*, false negatives), but we have seen no reason to expect measurable false-positive rates from strong reactions to SBB. SBB has also served as a widely trusted indicator of lipids in histopathology laboratories for a variety of disease states where false positives could have severe health and legal consequences. One might argue that the authors of the papers in Table 1 were using LM in their histochemistry studies and might have confused the Permeability Barrier Membrane with the Lipid-rich Layer and mistakenly assigned to Layer #3 a strong SSB reaction that should have been attributed to Layer #5. However, David Wharton is an author on all four of these papers and has been a proponent of Variant 1 of the trilaminar model (Figure 1), which assigns the permeability barrier function and the “Lipid-rich Layer” moniker to the electron-dense coating of the parietal surface of chitin (Electron-dense Parietal Coating). Also, in the majority of cases where TEMs of various species show both Layer #3 and Layer #5, Layer #3 is always many times thicker and usually much more electron dense than is the membranous Layer #5, and so in cases where Layers #3 and #5 might be combined in the SBB histochemistry assessments for lipids, Layer #5 must only be a minor contributor to the lipid-intensity ratings in Table 1 being “intense” or “very intense.”

**Table 1:** Some papers showing that Layer #3 exhibits moderate to very intense reaction to Sudan Black B, suggesting that Layer #3a does contain lipid, contradicting recent suggestions that the “inner eggshell layer” (of the classical trilaminar framework) “is composed [instead] of proteoglycans” [83, 103].

Group and species [authority]	Sudan Black B reaction^1^ of the Electron-dense Parietal Coating	References
Tylenchida	+ + +	Perry *et al.* (1982) [87]
*Globodera rostochiensis* [122]		
Oxyurida	+ +	Wharton (1979) [113]
*Hammerschmidtiella diesingi* [44]		
Oxyurida	+ + + + +	Wharton (1979) [115]
*Aspiculuris tetraptera* [101]		
Trichinelloidea	+ + + +	Wharton and Jenkins (1978) [120]
*Trichuris suis* [100]		

1 + +, moderate; + + +, strong; + + + +, intense; + + + + +, very intense reactions.

Another challenge to the conclusion that Layer #3 is not a lipid-rich layer is that osmium tetroxide (OsO_4_) was used as a fixative in all TEM sections where we checked the methods, and this chemical has been used historically as a lipid-indicating stain for decades and seems to be specific to unsaturated fats [12]. Layer #3a (Electron-dense Parietal Coating) almost always stains heavily with OsO_4_ in eggshell TEMs we have seen, and also stains, at least lightly, the inner boundary of the Chitinous Layer of most *C. elegans* TEMs (see references in Section 4.4.2). It remains to be determined definitively which lipids are involved in limiting diffusion into the nematode egg, but ascarosides are often implicated, and some ascarosides are unsaturated and would probably stain with OsO_4_. Also, OsO_4_ is normally applied early in TEM prep, and would stain lipid-rich tissues before they could be exposed to any lipid solvents associated with normal TEM protocol. Belazi *et al.* (2009) [12] demonstrated that even when solvents remove lipids following OsO4 fixation, some osmium stain stays behind as testimony of the former presence of lipid. Additionally, fig. 2A of Mansfield *et al.* (1992) [66] is a TEM section of a normal eggshell of *Haemonchus contortus* showing what appears to be the same osmophilic Layer #3a as most other species, and then shows in fig. 2E a similar view of an eggshell after having been first treated with a lipid solvent (chloroform/methanol) prior to OsO_4_ fixation, and almost all of the osmophilic material in Layer #3 was evidently removed by the solvent. However, almost as much was also removed by Proteinase K treatment, but this could be because the protein associated with the chitin, to which the lipids are bound, had been altered.

Although the authors of both papers on the Olson version of the hexalaminar framework carefully avoided stating explicitly that there was no such thing as a lipid-rich layer at position #3, they did imply as such in their wording; for example (from the section in Stein and Golden (2018) [103] entitled “5. Layer three: the CPG layer,” with emphasis (italics) ours:

The *third layer of the trilaminar* outer eggshell *had been thought to be a lipid-rich layer* for the past 100 years or more. The reason *this layer was thought to be lipid-rich* was based on chemical extraction and analysis (Chitwood, 1938…). … these new findings strongly suggest that *this third layer of the trilaminar outer eggshell is composed of proteoglycans*.

Given this language, which questions earlier research findings of proponents of a Lipid-rich Layer at position #3 and using definitive terms (“trilaminar outer eggshell,” “this third layer,” etc.) from the classical literature of the era of the phylum-wide application of the trilaminar eggshell framework, how can subsequent readers be faulted for concluding that there is no Lipid-rich Layer at position #3 of the generalized nematode eggshell? Evidence of the wide-spread interpretation of this quote to imply that Layer #3 should no longer be considered a Lipid-rich Layer can be found by a simple literature search for papers since 2012 that cite [83] or [103] as authorities for the author’s claim that Layer #5 is now the Lipid-rich Layer (instead of Layer #3). Also, none of the schematic eggshell models provided in papers authored by members of the *C. elegans* Research Community since about 2014 include a lipid layer at Position #3, but almost all prior to about 2012 either display or discuss the presence of lipids at Layer #3.

Also, in their schematic representations of the new model (fig. 9 of Olson *et al.* (2012) [83] and fig. 1 of Stein and Golden (2018) [103]), they substituted the CPG Layer into the position of Layer #3, and essentially moved the Lipid-rich Layer label (for the Electron-dense Parietal Coating) from position #3 to position #5 (the Permeability Barrier Membrane). In addition to [28], investigative literature later in the 20th Century provides other diverse and rigorous challenges against the claims of the *C. elegans* team that there are CPGs instead of lipids at layer #3, but neither the Olson *et al.* (2012) [83] nor Stein and Golden (2018) [103] papers even addresses those challenges, despite the scope and context of the related prose of these sections both papers being very clearly pertaining not just to *C. elegans*, but to the broader scope of the of trilaminar framework – the phylum at large.

So, it is one thing to say that Layer #3 contains CPG and the Permeability Barrier Membrane contains lipids, but to imply that it is wrong to refer to Layer #3 as a Lipid-rich Layer is challenged by several studies we have seen and is contradicted by the presence of an osmophilic layer between the Chitinous Layer and the CPG Layer in *C. elegans*. The burden of proof is now on the *C. elegans* Research Community to explain the consistent affinity of Layer #3a for SBB and OsO_4_ (even in *C. elegans*; fig. 4C of Benenati *et al.* (2009) [14]) and its dissolution with lipid solvents in Mansfield *et al.* (1992) [66] before any more suggestions are made that the Permeability Barrier Membrane (Layer #5) is *THE* Lipid-rich Layer of the generalized nematode eggshell.

A note of clarification – please understand that we do not want to diminish the contributions of the Olson model to nematodology; indeed, we consider it to be the most significant single advance in understanding of the nematode eggshell ever; truly a major milestone that will be cited heavily for many decades. However, the language quoted above has definitely misled the rest of the *C. elegans* community and some clarification should be brought to bear on this important issue.

#### 4.9.3 The function of Layer #3

Experiments performed by some authors also seem consistent with Layer #3a (the Electron-dense Parietal Coating) being the main barrier to diffusion, at least to some molecules in some species. Wharton (1980) [118] provided images suggesting that lead nitrate readily penetrated into the eggshell of *Hammerschmidtiella diesingi* all the way to the Electron-dense Parietal Coating and accumulated there but could not be found internal to the Electron-dense Parietal Coating at Layer #3. This was interpreted to mean that the Electron-dense Parietal Coating is the permeability barrier that inhibits water loss and prevents the passage of larger molecules. While these findings do suggest that the Electron-dense Parietal Coating is a barrier to passage of lead ions, they are not as persuasive that it inhibits the free passage of water. Indeed, Wharton’s imagery portrays a gradient of increasing lead concentration centripetally across the Chitinous Layer, with the ions piling up in the Electron-dense Parietal Coating (pl. 1A, Wharton (1980) [118]). This is not the pattern one would expect from a diffusion at equilibrium external to the Electron-dense Parietal Coating, but is exactly what one would expect if the Electron-dense Parietal Coating were allowing the free passage of water into the egg but had not allowed the passage of the lead ions. Another explanation for the increasing concentration at the Electron-dense Parietal Coating might be that some component of the Electron-dense Parietal Coating is serving as a diffusion sink for the lead ions and reducing osmotic pressure at that point by bonding with the ions and taking them out of solution.

Mansfield *et al.* (1992) [66] studied the bold and dark Layer #3a of *Haemonchus contortus* quite extensively, and while lipid solvents and Proteinase K both substantially reduced the electron opacity of the structure but neither entirely removed it, it is noteworthy that natural hatching more completely depleted both components than did either treatment.

#### 4.9.4 Summary of challenges

Almost the entire community of nematodologists from all disciplines (outside the *C. elegans* Research Community) has been faithfully using some variant of the classical trilaminar framework (and continues to use it in 2022) to label and explain the functional morphology of nematode eggshells. Until the aforementioned challenges from the historical literature are addressed, acceptance of the much more parsimonious hexalaminar framework will be unnecessarily but understandably resisted.

Several persuasive arguments have been made in support of the Electron-dense Parietal Coating of most species containing a substantial amount of lipids of some sort; for instance, the prominent Electron-dense Parietal Coating of *Haemonchus contortus* consisting of about half lipids and half proteins [66]; thus, Layer #3a just might be serving as the functional permeability barrier in some of those cases. On the contrary, equally persuasive arguments have been offered in support of Layer #3a having little or no lipids and not serving as the functional permeability barrier; for instance, the Wharton (1979) [114] study of the *Syphacia obvelata* eggshell, in which Wharton revealed that the eggshell exhibited “only doubtful positive histochemical reactions for lipids” and that the “lipid layer [Layer #3a, the Electron-dense Parietal Coating] does not form a significant permeability barrier.” Thus, it should not be surprising that we may be dealing with evolutionarily malleable compositions and functions among the layers internal to the Chitinous Layer.

### 4.10 A new hexalaminar anatomical model for *Huffmanela* egg studies

**Figure 31:**
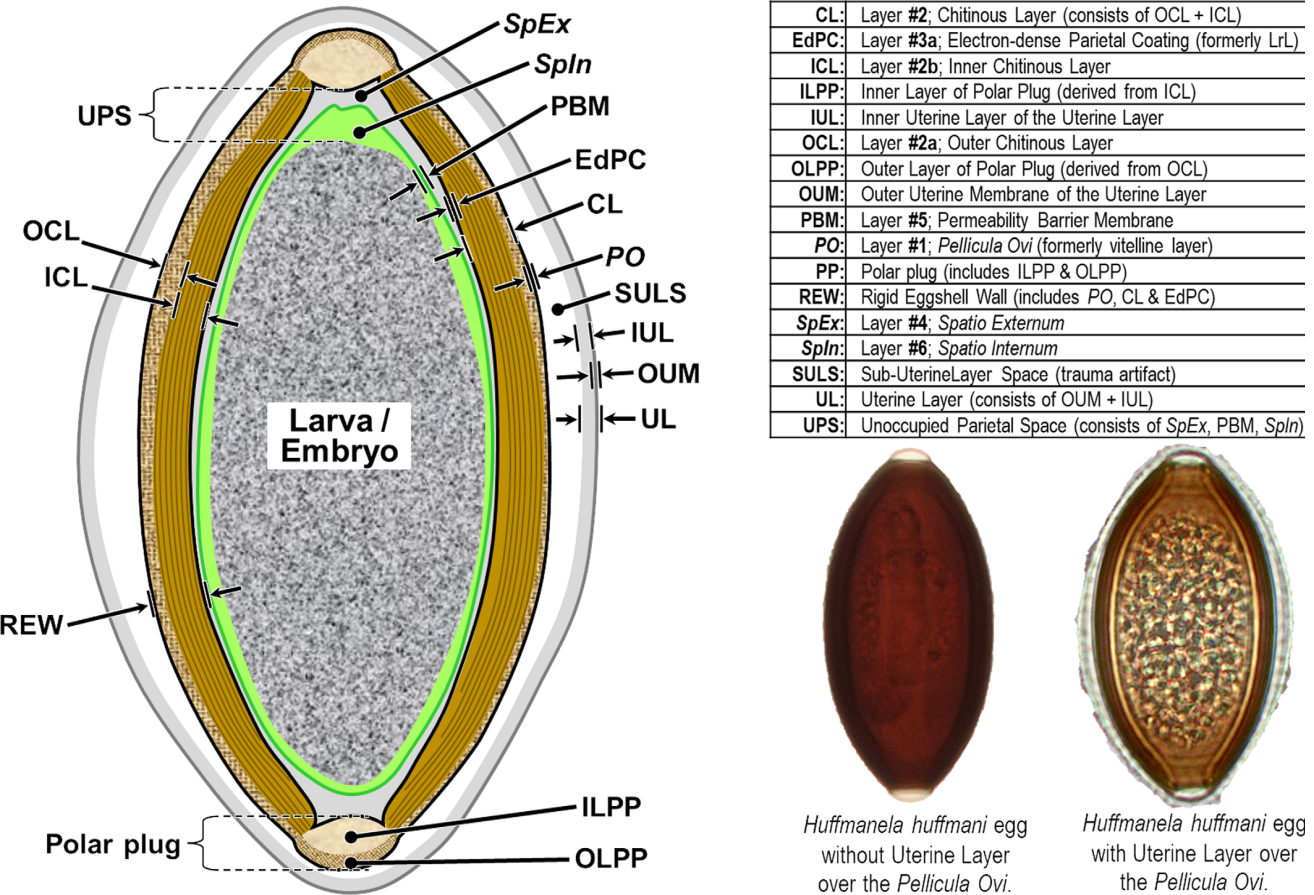
Drawing of *Huffmanela huffmani* eggshell with other details: a composite of several TEM images superimposed onto a tracing of a wholemount photographed with a light microscope (Surface Papillae not drawn on Uterine Layer).

Only two species of *Huffmanela* have been studied by TEM prior to this writing, the spring-dependent freshwater *H. huffmani* by Žďárská *et al.* (2001) [125], and an innominate marine clade from sharks by Attia *et al.* (2021) [7]. There is much variation in LM-discernible eggshell structure and features among various bipolar eggs that have been assigned to the genus, and it would be dangerous to speculate any more than we already have about the nature of the unusual features among the eggs of other *Huffmanela* species until more TEM studies are completed. Interestingly, the Inner Chitinous Layer of the Attia [7] clade in sharks appears to lack the Bouligand architecture that produces alternating dark and light lamellae, an unusual condition among thick-shelled trichinelloids that have been imaged. At this point, we can only make a strong case for the eggshell features of *H. huffmani* and hope that the hexalaminar framework we provide will stimulate subsequent TEM work of eggs preserved in curated collections for comparison with our findings. We provide in Figure 31 a new anatomical model of the *H. huffmani* eggshell to use as a standard for comparison. It is based on the Olson *et al.* (2012) [83] hexalaminar framework for *C. elegans* as modified by us and presented in Figure 2.

## 5 Conclusions

As we began to search for suitable terminological standards to help us understand the structures we were seeing in our TEMs of *Huffmanela huffmani*, we soon discovered a disappointing level of disregard for terminological integrity, not only in *Huffmanela* literature, but also the literature of other trichinelloid families and the phylum at large. Once we realized that the outermost layer of the *H. huffmani* eggshell is actually a uterine layer, the hexalaminar anatomical model set forth by Olson *et al.* (2012) [83] and modified by Stein and Golden (2018) [103] appeared to apply quite well to the egg structure of *H. huffmani* as revealed by TEM. It explained what we were finding in *Huffmanela* eggs much better than the classical trilaminar anatomical model. Similarly, other trichinelloid eggs (all of which imaged thus far lack a uterine membrane), as well as eggs from other orders (with and without uterine layers), also appeared to conform consistently to the new hexalaminar anatomical model. However, while the first draft [83] of the hexalaminar framework for *C. elegans* explained many mysteries about structures that previous workers had either ignored or struggled with in ways that made matters worse, the terminological scheme recommended by Stein and Golden (2018) [103] in the second draft of hexalaminar framework retained several terms from the dark ages of nematodology that are encumbered with decades of conflation and misapplication. It also focused on the ontogenetic program of *C. elegans* using modifiers pertaining to transient developmental functions in the prezygotic and early post-zygotic egg. Many of these terms are mnemonically disengaged from practical research associated with the more mature embryonated/larvated eggs that most nematodologists must communicate about on a routine basis. To make matters worse, the two papers revealing the new Olson framework were written expressly for an audience of molecular geneticists and were difficult for general nematodologists to understand. Thus, the new hexalaminar framework has flourished within the *C. elegans* Research Community since 2012, but even the anatomical component has been almost completely ignored by nematodologists and parasitology reference works. This has been the pattern that continues through today and will not be broken until a consistent framework of descriptively and mnemonically appropriate terminology is offered that can be used to harmonize the findings of the *C. elegans* Research Community with important findings from the classical nematode literature. Herein we have offered a third draft of the Olson (2012) [83] hexalaminar framework for reporting research findings involving nematode eggshells. We have expanded the *C. elegans* hexalaminar framework to include the polar regions of trichinelloids, and we have developed a new descriptively mnemonic terminological scheme that is unencumbered by historical ambiguity and is more appropriate for embryonated/larvated eggs. Example applications of the draft framework to important imagery in the classical nematodology literature have been provided to guide reinterpretation of the classical literature and to facilitate adoption by general nematodologists outside the *C. elegans* Research Community.

### 5.1 Some suggestions for future eggshell work

#### 5.1.1 Terminology

Here are some guidelines for alternative terms to replace historically ambiguous egg-anatomy terms in new reports from nematode eggshell studies:

- Avoid use of “trilaminar” to refer to the outer three layers because it is ambiguous (see Figure 1). The term “Rigid Eggshell Wall” (Figure 2) refers to the same three layers that make up the “true shell” and is unambiguous and mnemonic.
- “Peri-vitelline space,” “peri-embryonic space,” and “extra-embryonic matrix” are encumbered with historical and anatomical ambiguity and should not be used in reference to any part of an embryonated/larvated nematode egg. If there is no evidence of a Permeability Barrier Membrane (Figure 2) in an image of a premitotic eggshell, the entire space interior to the Chitinous Layer is the *Spatio Externum*. If there is a Permeability Barrier Membrane in the space, use all three labels included in the Unoccupied Parietal Space in Figure 2. In generic cases where the labels would be superfluous, the space between the Chitinous Layer and embryo/larva can be referred to unambiguously as the Unoccupied Parietal Space (Figure 2).
- Use of the terms “lipid layer” or “Lipid-rich Layer.” Some authors have appropriately used these terms to refer to an electron dense layer immediately internal to the chitin (now the Electron-dense Parietal Coating; Figure 2); however, that layer, while probably containing lipids, has not been proven to be *THE* lipid layer. When authors of earlier works are referring indirectly to the functional barrier protecting the embryo from osmotic stress, they are talking about a function that probably belongs to Layer #5, the Permeability Barrier Membrane [103]. This function has been definitively demonstrated for Layer #5 of *C. elegans* [83] and also of *Ascaris lumbricoides* [38], and Layer #5 appears to be almost universally present in TEMs of mitotic nematode eggs (Section 4.5.2). However, Layer #3a (Electron-dense Parietal Coating; formerly Lipid-rich Layer) is very faint in *C. elegans*, while bold and prominent in other species like *Haemonchus contortus*, and some of the burden of barrier-to-permeability may be shifted from Layer #5 to Layer #3a in such species.
- To minimize the likelihood of ambiguous interpretation of one’s findings, don’t use the terms “vitelline layer,” “vitelline membrane,” “vitelline coat” or “vitelline- [anything else]” in reference to any structure in a nematode egg.
- When referring directly to the function of the layer that appears almost immediately after sperm penetration and temporarily serves as the first barrier protecting the vitellus from polyspermy, use of the term ‘fast block to polyspermy” might be more appropriate.
- When referring to the uniformly electron dense and uniformly thin membranous layer on the outer surface of the Chitinous Layer, use the term *Pellicula Ovi*.
- When referring to the apparently deformable membrane suspended in the fluid internal to the Chitinous Layer (sometimes previously called the “inner vitelline membrane” or “embryonic membrane”), use the term Permeability Barrier Membrane (Layer #5).

#### 5.1.2 New *Huffmanela* populations

All occurrences of *Huffmanela* eggs from new fish families or substantially new localities should be reported, even if adults are not found and TEM is not possible. Note that the vastness of the Indian Ocean, except for its northwestern most extensions, is entirely unrepresented by *Huffmanela* reports, while the lagoons surrounding the main island of New Caledonia have yielded no less than seven nominal species of the genus, only because one scientist knew how to look for a *Huffmanela* infection [55–58] surveyed fish there. That pattern of discovery could probably be replicated at locations around the Indian Ocean. Anyone interested in surveying fish for helminths should familiarize themselves with the gross appearance of typical lesions caused by *Huffmanela* spp. infection by perusal of imagery provided in many papers [3, 7, 8, 24, 34, 37, 57, 64, 90, 91, 95, 96].

We provide in Figure 31 a drawing of an *H. huffmani* egg labeled according to the new framework.

## 6 Glossary and anatomical abbreviations

- **Chitinous Layer** (**CL**; Layer **#2**; Figure 2): Responsible for rigidity of the Rigid Eggshell Wall. Usually consists of a usually thinner outer layer (Outer Chitinous Layer) and a usually thicker inner layer (Inner Chitinous Layer).
- **Cortical granules (CG)**: Formerly refringent or refractile granules. This is a generic term that refers to various species of vesicles in the cortical cytoplasm of the oocyte or zygote that, when triggered to do so, fuse with the oolemma and discharge cargos. The cargos of various species of cortical granules produce most layers of the eggshell, among other things.
- **CPG Layer** (**CPGL**; Layer **#3b**; Figure 2): At this writing, only applies to the eggshell of *Caenorhabditis elegans*. It is found in the *C. elegans* eggshell just internal to the Electron-dense Parietal Coating (Layer #3b) and was first described by [83].
- **CPG**: In the context of the nematode eggshell, this acronym refers to either of two species (CPG-1 or CPG-2) out of nine novel chondroitin proteoglycan (glycosylated protein) molecules discovered in *Caenorhabditis elegans* by Olson *et al.* (2006) [82], the first two of which are components of the CPG Layer of *C. elegans* (discovered by Olson *et al.* (2012) [83]).
- **Dense granules** (**DG**): Oocyte inclusions that stain darkly in TEMs. These are sometimes found as inclusions in cortical granules, and some of them appear to contribute to the formation of Layers #3 and #4.
- **DIC**: Differential Interference Contrast (or Nomarski) microscopy. Light-based microscopy technology recommended over bright-field LM and phase contrast for study of nematode eggshells, especially thick-shelled trichinelloid eggs.
- **Electron-dense Parietal Coating** (**EdPC**; Layer **#3a**; Figure 2): Electron dense coating applied to the parietal surface of the eggshell immediately after the Chitinous Layer is completed, and discernible on almost all standard TEMs of zygotic nematode eggs that include a view of the parietal surface of the Chitinous Layer. Formerly referred to as lipid (-rich) layer (ll or LrL).
- **Fast block to polyspermy**: The functional name that can be used to refer to the *Pellicula Ovi* after the first sperm has successfully entered the oocyte cytoplasm while it is still preventing other sperm from penetrating the plasma lemma of the oocyte. This is a functional term – the anatomical name of the physical structure that provides this ephemeral service to the nematode oocyte is *Pellicula Ovi*.
- **Inner Chitinous Layer** (**ICL**; Layer **#3b**; Figure 2): In many nematode eggshells, Layer #2, the Chitinous Layer, consists of two layers having different electron densities and/or fibrillar arrangements, the Outer Chitinous Layer and the Inner Chitinous Layer. The latter is usually laminated in Trichinelloidea.
- **Inner Layer of Polar Plug** (**ILPP**; Figure 2): Applies only to trichinelloid polar plugs, derived from the Inner Chitinous Layer (Layer **#2b**) but usually less electron dense.
- **Inner Uterine Layer** (**IUL**; not numbered): Usually much thicker than the Outer Uterine Membrane.
- **Lipid-rich Layer** (**LrL**; see Electron-dense Parietal Coating): Although usually applied (in literature prior to 2012) to Layer #3a (the Electron-dense Parietal Coating; Figure 2), it has also been applied to several other layers of the nematode eggshell and is no longer recommended for use as a noun naming any structure of the nematode eggshell because of historic ambiguity.
- **LM:** Light Microscopy. Use of this abbreviation in this report is usually referring to simple bright-field LM and sometimes includes phase contrast microscopy.
- **Outer Chitinous Layer** (**OCL**; Layer **#2a**; Figure 2): The outermost layers of chitin deposited onto the parietal surface of the *Pellicula Ovi* immediately after fertilization and serving as the “slow block to polyspermy.” Not always distinguishable from the Inner Chitinous Layer in TEMs. Sometimes occurs as a mosaic of what appears to be an open weave of fibers or open spaces that are sometimes geometrically complex in capillariids and perhaps others.
- **Outer Layer of Polar Plug** (**OLPP**; Figure 2): Applies primarily to trichinelloid eggs; apparently derived from Layer #2a, the Outer Chitinous Layer.
- **Outer Uterine Membrane** (**OUM**; unnumbered): A thin, electron-dense membrane, sometimes resembling a lipid bilayer, lining the outer surface of the Uterine Layer of some species that have a Uterine Layer.
- **Parietal surface**: The inner surface of a container wall or layer surrounding an enclosed cavity and its contents, as in the parietal peritoneum being the lining of the abdominal coelom of a mammal.
- ***Pellicula Ovi*** (**PO**; Layer **#1**; Figure 2): The outermost layer of a fertilized oocyte, and afterward of the eggshell until hatching. Formerly called the vitelline membrane, among many other things.
- **Permeability Barrier Membrane** (**PBM**; Layer **#5**; Figure 2): A deformable membrane that separates the *Spatio Externum* from the *Spatio Internum* and serves as the true barrier to molecular permeability, at least in *C. elegans* [83] and *Ascaris lumbricoides* [38]. Sometimes the Electron-dense Parietal Coating is referred to as the permeability barrier and may be providing that function, at least partially, in some species.
- **Polar canal**: Refers to the nearly cylindrical hole remaining in the end of a trichinelloid eggshell Rigid Eggshell Wall after the polar plug has been removed.
- **Rigid Eggshell Wall** (**REW**): Consists of Layers #1–3. It’s the term that should be used to refer to the “hard shell,” “true shell,” or “wall” of the nematode egg.
- **SBB**: Sudan Black B. A histochemical stain specific to staining lipids black.
- **SEM**: Scanning Electron Microscopy.
- **Slow block to Polyspermy**: A reference to the early function of the Chitinous Layer in nematode eggs, which inherits the role of a backup barrier-to-polyspermy from the *Pellicula Ovi* immediately after the first thin layers of the Chitinous Layer completely coat the parietal surface of the *Pellicula Ovi*.
- ***Spatio Externum*** (**SpEx**; Layer **#4**; Figure 2): The deformable, usually fluid-filled space between Layer #3 (which coats the parietal surface of the Chitinous Layer) and the Permeability Barrier Membrane (Layer #5). Formed after anaphase I; receives polar body #1.
- ***Spatio Internum*** (**SpIn**; Layer **#6**; Figure 2): The deformable, usually fluid-filled space between the Permeability Barrier Membrane (Layer #5) and the oolemma of the zygote/embryo (or larval cuticle). Formed after anaphase II; receives polar body #2.
- **Sub Uterine-Layer Space** (**SULS**): An artifactual space sometimes created between the Inner Uterine Layer and the *Pellicula Ovi* after eggs are physically disturbed.
- **Surface Papillae** (**SP**): These are circular bumps to papilla-like projections formed by the uterine layer of *Huffmanela* eggs.
- **TEM**: Transmission Electron Microscopy.
- **Unoccupied Parietal Space** (**UPS**): The space in the cavity formed by the Rigid Eggshell Wall (the cavity internal to Layer #3) that is not occupied by the embryo/larva. Consists of Layers #4–6.
- **Uterine Layer** (**UL**): An unnumbered exogenous layer of the eggshell not obviously present in many nematode groups. Deposited onto the outer surface of the *Pellicula Ovi* by the uterine lining. Often (but not always) consists of the membranous Outer Uterine Membrane and the much thicker Inner Uterine Layer.
- **VL**: Vitelline Layer. A term formerly applied mainly to Layer #1 (the *Pellicula Ovi*), but also to Layer #5 and the Uterine Layer. Encumbered with historical ambiguity and no longer recommended for any structure of any nematode eggshell.
